# *Sarm1* knockout prevents type 1 diabetic bone disease in females independent of neuropathy

**DOI:** 10.1172/jci.insight.175159

**Published:** 2024-01-04

**Authors:** Jennifer M. Brazill, Ivana R. Shen, Clarissa S. Craft, Kristann L. Magee, Jay S. Park, Madelyn Lorenz, Amy Strickland, Natalie K. Wee, Xiao Zhang, Alec T. Beeve, Gretchen A. Meyer, Jeffrey Milbrandt, Aaron DiAntonio, Erica L. Scheller

**Affiliations:** 1 Division of Bone and Mineral Diseases, Department of Medicine, and; 2 Department of Genetics, Washington University School of Medicine, St. Louis, Missouri, USA.; 3 Department of Biomedical Engineering, McKelvey School of Engineering, Washington University, St. Louis, Missouri, USA.; 4 Program in Physical Therapy,; 5 Department of Developmental Biology, and; 6 Department of Cell Biology and Physiology, Washington University School of Medicine, St. Louis, Missouri, USA.

**Keywords:** Bone Biology, Endocrinology, Bone disease, Diabetes, Neurodegeneration

## Abstract

Patients with diabetes have a high risk of developing skeletal diseases accompanied by diabetic peripheral neuropathy (DPN). In this study, we isolated the role of DPN in skeletal disease with global and conditional knockout models of sterile-α and TIR-motif-containing protein-1 (*Sarm1*). SARM1, an NADase highly expressed in the nervous system, regulates axon degeneration upon a range of insults, including DPN. Global knockout of *Sarm1* prevented DPN, but not skeletal disease, in male mice with type 1 diabetes (T1D). Female wild-type mice also developed diabetic bone disease but without DPN. Unexpectedly, global *Sarm1* knockout completely protected female mice from T1D-associated bone suppression and skeletal fragility despite comparable muscle atrophy and hyperglycemia. Global *Sarm1* knockout rescued bone health through sustained osteoblast function with abrogation of local oxidative stress responses. This was independent of the neural actions of SARM1, as beneficial effects on bone were lost with neural conditional *Sarm1* knockout. This study demonstrates that the onset of skeletal disease occurs rapidly in both male and female mice with T1D completely independently of DPN. In addition, this reveals that clinical SARM1 inhibitors, currently being developed for treatment of neuropathy, may also have benefits for diabetic bone through actions outside of the nervous system.

## Abstract 2

In murine and diabetes and metabolic disease, inhibition of SARM1 prevents diabetic peripheral neuropathy and, in female mice, improves skeletal health.

## Graphical abstract

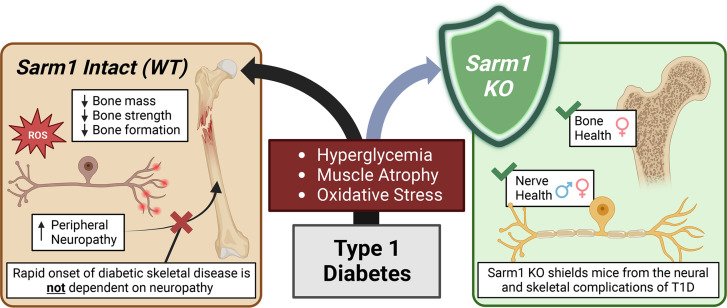

## Introduction

Type 1 diabetes (T1D) is characterized by the immune-mediated destruction of pancreatic β cells that culminates in insulin deficiency and hyperglycemia (1). Diagnosis of T1D is most common during youth and adolescence, and a recently released Type 1 Diabetes Index model predicts that the prevalence of worldwide T1D will double by 2040 (1, 2). Unfortunately, the metabolic derangements of T1D predispose to the onset of diabetic complications that can affect most tissues and organs throughout the body, leaving patients at risk for chronic disability and increased mortality (1, 3). These widespread complications share underlying mechanisms of onset, including cellular bioenergetic stress, oxidative damage, and inflammation (4). Identification of common target points for intervention that will lead to improvements across multiple systems is key to improving both health and life span of patients with T1D.

It is becoming increasingly recognized that diabetic complications manifest early in the course of disease (3, 5). Consistent with this, 32% of adolescents with T1D and 72% with T2D already have 1 reported complication (3). In addition, bone fracture risk in patients with T1D is elevated in the youngest 0- to 19-year-old age group (6). Similarly, 5.8% of adolescent patients with 5 to 10 years of T1D duration have clinical diabetic peripheral neuropathy (DPN); this increases to 14.7% with durations longer than 10 years in patients under 21 years of age based on results from the SEARCH for Diabetes in Youth Study (7). Asymptomatic, subclinical neuropathy may be even more prevalent in this age group, with estimates ranging from approximately 20% to 65% (8–10).

The presence and severity of DPN in T1D has been independently associated with reduced bone mineral density, diminished bone quality, or fracture in 10/14 clinical studies to date (11–13). There are 2 main paths that connect the function of the peripheral nervous system with skeletal health in diabetes (reviewed in ref. 11). First, neuropathic changes in DPN are well established to increase the risk of falls in patients with T1D, consequently elevating the potential for fracture. Second, changes in nerve density and function due to DPN may increase fracture risk due to direct impairment of bone cell function and the skeletal vascular supply. This is in line with recent studies that have highlighted the potential relevance of neuroskeletal function to bone health in settings of development, homeostasis, and regeneration (reviewed in refs. 14, 15). Together, this has prompted the hypothesis that neural dysfunction, through the onset of DPN, may be responsible for the onset and progression of diabetic skeletal disease — representing a possible unified target point for treatment of both disorders.

In this study, we used global and conditional knockout models of sterile-α and TIR-motif-containing protein-1 (*Sarm1*) to isolate the role of DPN in the onset of T1D skeletal disease. SARM1 is the central executioner of nerve axon degeneration (16, 17). SARM1 is also a founding member of the TIR-domain class of NADases that is highly expressed in the nervous system (18–21). The SARM1 enzyme serves as a metabolic biosensor (22) that is activated in response to injury, inflammation, and oxidative stress (23–26). *Sarm1* knockout is sufficient to protect nerve axon structure and function in animal models of traumatic axonal injury (27); chemotherapy-induced, inflammatory, and CMT2A models of peripheral neuropathy (28–30); as well as mouse models of both T1D- and T2D-associated DPN (31, 32). In addition, SARM1 inhibitors are currently under clinical development for treatment of neurodegenerative conditions, including multiple sclerosis, amyotrophic lateral sclerosis, Parkinson’s disease, optic neuropathies, DPN, and chemotherapy-induced peripheral neuropathy (33, 34). Thus, in addition to clarifying the mechanistic relationship between DPN and skeletal disease in vivo, we believe our results can inform the future use of SARM1 inhibitors as a novel strategy to promote diabetic neuroskeletal health.

## Results

### Co-occurrence of skeletal and neural complications requires severe T1D.

**Figure 1:**
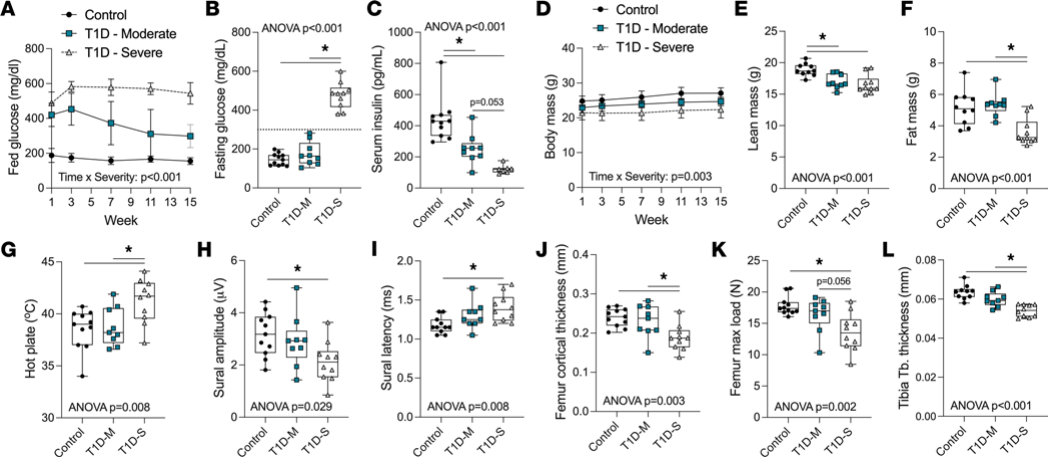
Changes in nerve function and skeletal health co-occur in male mice with severe, but not moderate, T1D. Male C57BL/6J mice at 12 to 13 weeks of age were injected twice with 100 mg/kg STZ to induce T1D; controls received 0.9% saline. All STZ-treated mice met standard criteria for T1D (2 serial fed glucose readings > 300 mg/dL within 1 week after injection). Mice were subsequently monitored from 1 to 15 weeks. (**A**) Longitudinal fed glucose. (**B**) Fasting glucose at week 14 (age 26 to 27 weeks). (**C**) Serum insulin. Blood was collected 30 minutes after glucose bolus IP and measured using the insulin Singulex assay (core service). (**D**) Body mass. (**E** and **F**) Lean and fat mass measured by DXA scan at week 14. (**G**) Ramping hot plate. (**H** and **I**) Electrophysiology (VikingQuest). Amplitude measured from baseline to the first peak (total fiber recruitment); latency represents time to first peak (a measure of myelination/axon response). (**J**) Femur mid-diaphyseal cortical thickness (Scanco μCT-40). (**K**) Maximum load to fracture, biomechanical testing of femurs using a servohydraulic testing machine (8841 Dynamite, Instron). (**L**) Tibia proximal metaphysis trabecular thickness (Scanco μCT-40). (**A** and **D**) Two-way ANOVA for severity × time. All others 1-way ANOVA. *P < 0.05. Graphed as mean ± SD (**A** and **D**) or box-and-whisker plots, where box plots show the interquartile range, median (line), and minimum and maximum (whiskers). (**B** and **C**, **E**–**L**). *n* = 9–11/group. DXA, dual x-ray absorptiometry; T1D-M, moderate T1D (fasting glucose < 300 mg/dL); T1D-S, severe (fasting glucose > 300 mg/dL).

Streptozotocin-induced (STZ-induced) T1D is well established as a model of both diabetic bone disease (35–37) and DPN (reviewed in ref. 38). However, there is substantial variability in the literature regarding the timing, prevalence, and severity of these pathologies. In addition, these complications have never been examined concurrently in the same cohort of animals to our knowledge. To address this, we characterized 3 groups of male mice at 7 months of age, 14 weeks after induction of T1D with our standard 2-dose regimen of STZ: control nondiabetic, moderate T1D (fasting glucose < 300 mg/dL), and severe T1D (fasting glucose > 300 mg/dL) (Figure 1, A and B). Maximal suppression of circulating insulin occurred in mice with severe, but not moderate, T1D (Figure 1C). Consistent with stratification of fasting glycemia, body mass was reduced to a greater extent in mice with severe T1D (Figure 1D). This was due to loss of muscle only in the moderate T1D group but loss of both muscle and adipose tissue in the severe group (Figure 1, E and F).

Next, we performed rotarod and inverted screen, measures of motor function and general well-being. There were no significant differences between groups (Supplemental Table 1; supplemental material available online with this article; https://doi.org/10.1172/jci.insight.175159DS1), providing a positive assessment of the overall health of the animals. We then quantified the response to ramping hot plate, a measure of activation of cutaneous nociceptors that is well suited to diabetic rodents. Responses were blunted in the group with severe, but not moderate, T1D (Figure 1G). Similarly, electrophysiology of the sural nerve revealed a 33% decrease in amplitude and 19% increase in latency of peripheral impulses in the severe T1D group only (Figure 1, H and I) — indicating decreased axon function. Similar results were observed by electrophysiology of the sciatic nerve (Supplemental Table 1). We did not observe gross structural changes in cutaneous nerve fibers at this time point based on immunostaining for calcitonin gene-related peptide and protein gene product 9.5 in the hind paw skin (Supplemental Table 1). Overall, this demonstrates the onset of functional DPN in mice with severe, but not moderate, T1D after 14 weeks of disease.

We also quantified bone morphology and strength. Tibia length was relatively preserved (17.8 ± 0.2 mm control versus 17.4 ± 0.4 mm severe T1D, Supplemental Table 1), verifying a limited impact on longitudinal growth. As with neuropathy, mice with severe T1D experienced significant decreases in cortical bone area (–20% femur, –22% tibia, *P* < 0.001, Supplemental Table 1) and cortical thickness (–22% femur, *P* < 0.002; –21% tibia, *P* < 0.001; Figure 1J and Supplemental Table 1) and associated increases in fragility (–24% max load, *P* < 0.002; Figure 1K). Cancellous bone in the proximal tibial metaphysis demonstrated a trending decrease in bone volume fraction in the severe T1D group (–18%, *P* < 0.213) that was driven by underlying decreases in trabecular thickness (–15%, *P* < 0.001, Figure 1L). Additional bone morphology and biomechanical testing outcomes are available in Supplemental Table 1.

Taken together, these results demonstrate that stratification based on fasting glucose more accurately defines mice with maximally suppressed circulating insulin. In addition, within this severe T1D group, both bone loss/fragility and development of DPN co-occur. Based on this, an inclusion criterion of sustained fasting glucose greater than 300 mg/dL for T1D animals was set for all subsequent experiments.

### Healthy Sarm1^KO^ mice phenocopy control littermates.

To test the relationship between nerve function and bone health, we selected the *Sarm1*-knockout (Sarm1^KO^) mouse model. SARM1 is a pro-neurodegenerative NADase, and Sarm1^KO^ mice have been previously reported to resist the onset of neuropathy in settings of T1D and T2D (31, 32). Consistent with prior reports (31, 39), male and female Sarm1^KO^ mice were phenotypically indistinguishable from wild-type (WT) control littermates. Specifically, WT and Sarm1^KO^ mice at 8 weeks of age had comparable blood glucose, body mass, body composition, nerve function, and bone morphology apart from a slight increase in trabecular number in Sarm1^KO^ animals (+18% in males, +13% in females; Supplemental Table 2). No changes were noted in cortical bone.

### WT and Sarm1^KO^ mice develop comparable T1D-associated metabolic disease, suppression of IGF-1, and muscle atrophy.

**Figure 2:**
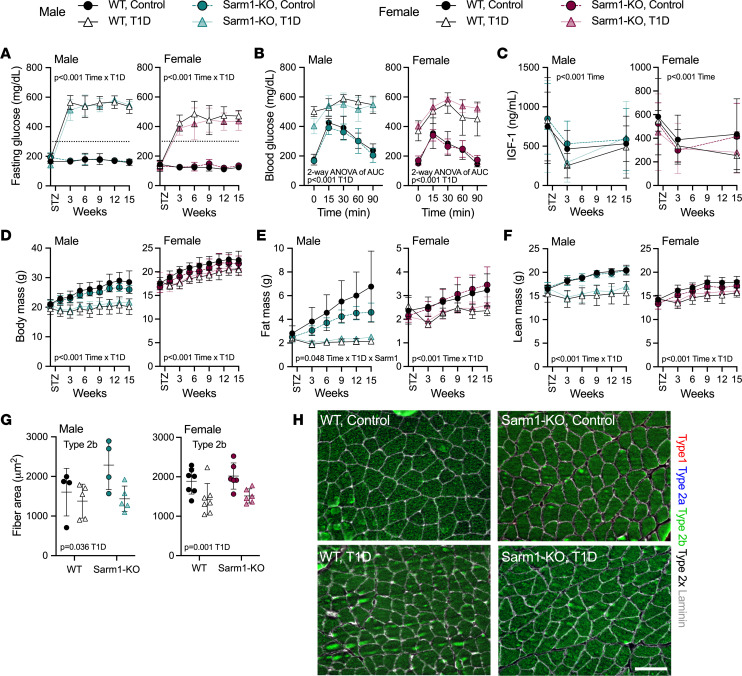
STZ-induced T1D impairs metabolism, body composition, and muscle architecture of WT control and Sarm1^KO^ mice, independent of genotype. WT control and Sarm1^KO^ mice at 8 weeks of age were treated with STZ to induce T1D; controls received 0.9% saline. Mice were monitored longitudinally for 15 weeks after treatment. (**A**) Fasting blood glucose. (**B**) Glucose tolerance test at 3 weeks after induction of T1D. (**C**) Plasma IGF-1 by ELISA. (**D**) Body mass. (**E** and **F**) Fat and lean mass as measured by EchoMRI. (**G**) Gastrocnemius muscle fiber cross-sectional area at 15 weeks after induction of T1D. (**H**) Representative original magnification, 20×, images of type 1 (red), type 2a (blue), and type 2b (green) muscle fibers by immunostaining for myosin heavy chain isoforms with unstained fibers designated as type 2x (black). Laminin immunostaining (gray) labeled the fiber perimeter and facilitated quantification. Scale bar = 100 μm. (**A**, **C**, **D**, **E**, and **F**) Three-way ANOVA for genotype × T1D × time. (**B**) Two-way ANOVA for genotype × T1D of area under the curve. (**G**) Two-way ANOVA for genotype × T1D. Graphed as mean ± SD. *n* = 4–7/group.

After induction of severe T1D with STZ at 8 weeks of age (inclusion criterion of sustained fasting glucose > 300 mg/dL), both WT and Sarm1^KO^ mice developed equivalent hyperglycemia that persisted throughout disease duration (Figure 2A). Onset of T1D-associated hyperglycemia in Sarm1^KO^ mice occurred in both males and females, though T1D males had a higher average fasting glucose than females, independent of genotype (541 ± 43 versus 443 ± 53 mg/dL, *P* < 0.001, Figure 2A). Glucose intolerance by glucose tolerance test (GTT) was also apparent at 3 weeks following T1D induction in both WT and Sarm1^KO^ mice (Figure 2B). Within each sex grouping, WT and Sarm1^KO^ mice with T1D also had comparable changes in circulating IGF-1, a hormone produced by the liver in response to insulin levels that is vital for postnatal growth (Figure 2C). This coincided with T1D-associated decreases in body mass in both males and females that could be attributed to lower muscle and fat mass in these animals, independent of genotype (Figure 2, D–F). Mice with T1D also had decreased cross-sectional area of type 1, 2a, 2b, and 2x muscle fibers in females (plantaris, soleus, and gastrocnemius muscles) and type 2b fibers in males (gastrocnemius muscle only), independent of genotype (Figure 2, G and H, and Supplemental Figure 1). *Sarm1* gene expression in the dorsal root ganglia (DRGs) was largely unaltered by the onset of T1D (Supplemental Figure 2). Together, these results show that T1D-associated changes in metabolism, body composition, and muscle were identical in WT and Sarm1^KO^ mice. These phenotypes also manifested similarly in both males and females apart from some sex-specific differences in the overall magnitude of the effects.

### Male Sarm1^KO^ mice resist the onset of DPN; female mice with T1D do not develop DPN during the 15-week experimental period.

**Figure 3:**
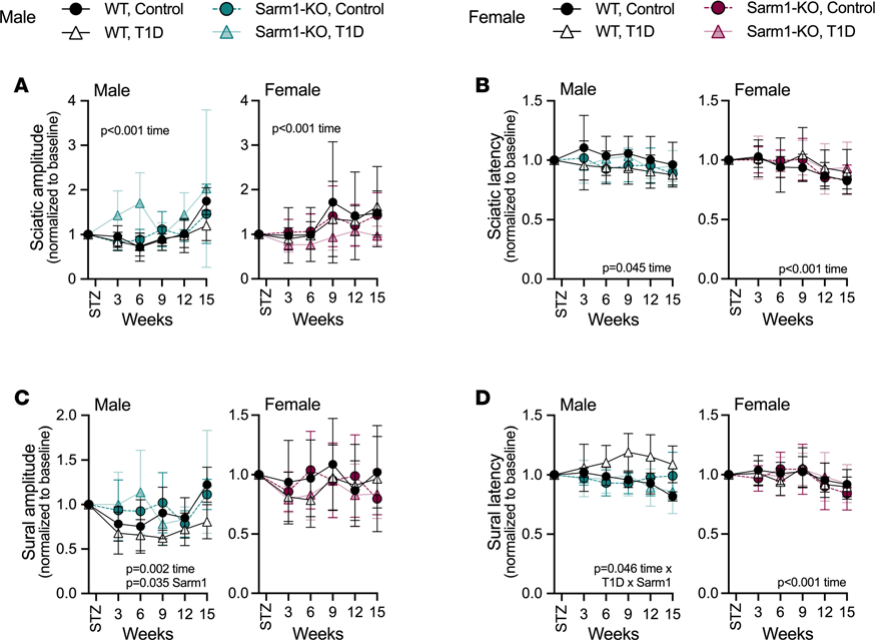
Sarm1^KO^ prevents the onset of length-dependent neuropathy in male mice with T1D; females do not develop T1D-associated nerve dysfunction during the 15-week experimental period regardless of genotype. WT control and Sarm1^KO^ mice at 8 weeks of age were treated with STZ to induce T1D; controls received 0.9% saline. Electrophysiological compound muscle action potentials (CMAPs) of both male and female mice were measured at baseline and longitudinally for 15 weeks after treatment. (**A** and **B**) CMAP amplitude (**A**) and latency (**B**) measured at the rear paw following stimulation at the sciatic notch. (**C** and **D**) CMAP amplitude (**C**) and latency (**D**) measured at the rear paw following stimulation at the ankle. Three-way ANOVA/mixed model for genotype × T1D × time. Each individual normalized to baseline; normalized data were plotted as mean ± SD. *n* = 4–7/group.

We next used longitudinal electrophysiology to assess the onset and severity of DPN in WT and Sarm1^KO^ mice over 15 weeks, from 8 to 23 weeks of age. This included measurement of the compound muscle action potential amplitude and latency as in Figure 1, which reflects axon number and conduction speed within the sciatic and sural nerves. Male WT mice showed functional signs of length-dependent neuropathy following induction of T1D (Figure 3), particularly deficits in distal nerve conduction speed (Figure 3D). Knockout of Sarm1 protected male mice against the development of DPN through the full 15 weeks of T1D. Unlike males, WT and Sarm1^KO^ female mice did not develop T1D-associated nerve dysfunction by electrophysiological measures throughout the duration of the experiment despite sustained severe hyperglycemia (Figure 3).

### Rescue of neuropathy in Sarm1^KO^ males does not prevent the onset and progression of diabetic skeletal disease.

**Figure 4:**
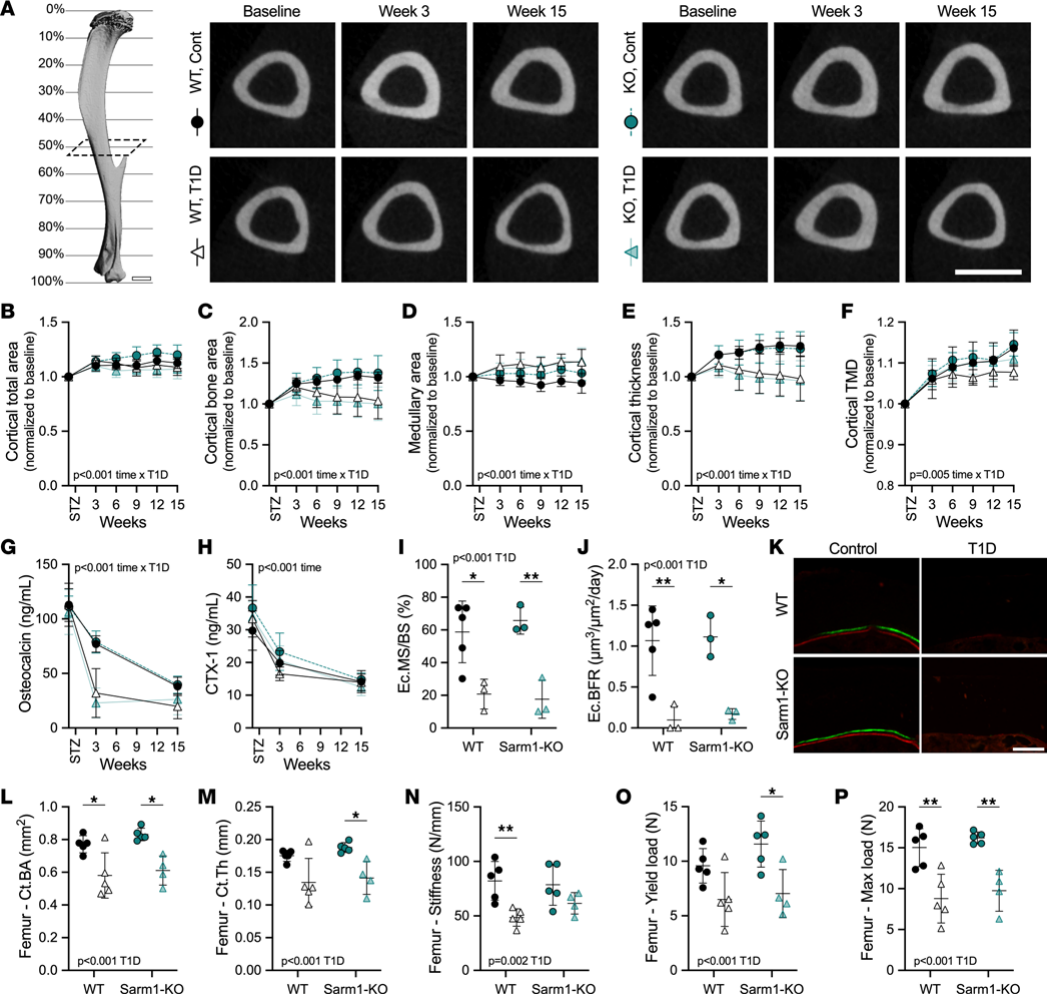
Male WT and Sarm1^KO^ mice develop comparable T1D bone disease. WT control and Sarm1^KO^ mice at 8 weeks of age were treated with STZ to induce T1D; controls received 0.9% saline. (**A**) Longitudinal in vivo microCT scans of the tibia were taken at the mid-diaphysis (~50% site). Representative 2D transaxial projections at baseline and 3 and 15 weeks of T1D. Scale bar = 1 mm. Tibia cortical total area (**B**), bone area (**C**), marrow area (**D**), cortical thickness (**E**), and cortical tissue mineral density (TMD, **F**) analyzed by in vivo microCT. (**G** and **H**) Plasma bone biomarkers osteocalcin (**G**) and type I collagen cross-linked C-telopeptide (CTX-1, **H**) measured by ELISA. (**I** and **J**) Endocortical mineralizing surface normalized to bone surface (**I**) and bone formation rate normalized to bone surface (**J**) analyzed from calcein (green)/alizarin (red) incorporation in the mid-diaphysis (~50% site) of the tibia at 3 weeks of T1D with representative micrographs of the medial surfaces (**K**). Scale bar = 100 μm. Femoral cortical bone area (**L**) and cortical thickness (**M**) analyzed by ex vivo microCT at 15-week endpoint. (**N**–**P**) Stiffness, yield load, and max load of the femur upon 3-point bend test. (**B**–**H**) Three-way ANOVA/mixed model for genotype × T1D × time. (**B**–**F**) Each individual normalized to baseline. (**I**–**P**) Two-way ANOVA for genotype × T1D with Tukey’s multiple comparisons test. All data plotted as mean ± SD. *n* = 3–5/group. *P < 0.05, **P < 0.01.

To summarize the results in males, both WT and Sarm1^KO^ mice developed comparable T1D-associated metabolic disease and muscle atrophy during the 15-week analysis period. By contrast, male WT mice with T1D developed DPN, and this was prevented in male Sarm1^KO^ animals. This provided an opportunity to subsequently isolate the role of Sarm1-dependent neuropathy in the onset and progression of diabetic skeletal disease (WT T1D with DPN versus Sarm1^KO^ T1D without DPN). To evaluate the onset and severity of diabetic bone disease, we initially assessed morphological properties of the tibia longitudinally by in vivo micro-computed tomography (microCT). In male WT and Sarm1^KO^ mice with T1D, cortical bone accrual was suppressed (Figure 4, A–F), resulting in thinner, less dense cortical bone as early as 3 weeks after the induction of T1D. Additional T1D-associated deterioration of cortical bone was noted from 3 to 15 weeks of disease (Figure 4, A–F). Accrual of trabecular thickness was similarly suppressed with T1D in male WT and Sarm1^KO^ mice (Supplemental Figure 3). With age, this would be expected to result in substantial trabecular bone loss.

To assess bone cell function, circulating biomarkers of bone metabolism were measured at baseline, acutely after 3 weeks of T1D, and at the 15-week study endpoint (Figure 4, G and H). Osteocalcin was markedly reduced after only 3 weeks in WT T1D (–58%) and Sarm1^KO^ T1D mice (–71%), indicating that osteoblast activity was repressed early in the course of disease (Figure 4G). Plasma type 1 collagen cross-linked c-telopeptide (CTX-1), a biomarker of osteoclast function, was significantly decreased over time in all groups but was not significantly altered by T1D in our data set (Figure 4H). Suppression of osteoblast function was further investigated with dynamic histomorphometry at the 3-week time point. Consistent with the longitudinal microCT and bone biomarker data, endocortical mineralizing surface and bone formation rate were reduced by 69% and 88% on average in mice with T1D, independent of genotype (Figure 4, I–K).

Last, at the 15-week endpoint, we performed microCT scans and biomechanical testing of the femur. Cortical morphological deficits observed in the tibia were also present in the femur (Figure 4, L and M, and Supplemental Figure 4). In addition, 3-point bending revealed diminished force to fracture and compromised biomechanical properties in both groups with T1D (Figure 4, N–P, and Supplemental Figure 4). Together, this shows that the protection from neuropathy afforded by Sarm1^KO^ in male mice did not prevent or in any way mitigate the rapid development and progression of diabetic skeletal disease in males.

### Global KO of Sarm1 prevents the onset of T1D bone disease in females, independent of neuropathy and the neural actions of SARM1.

**Figure 5:**
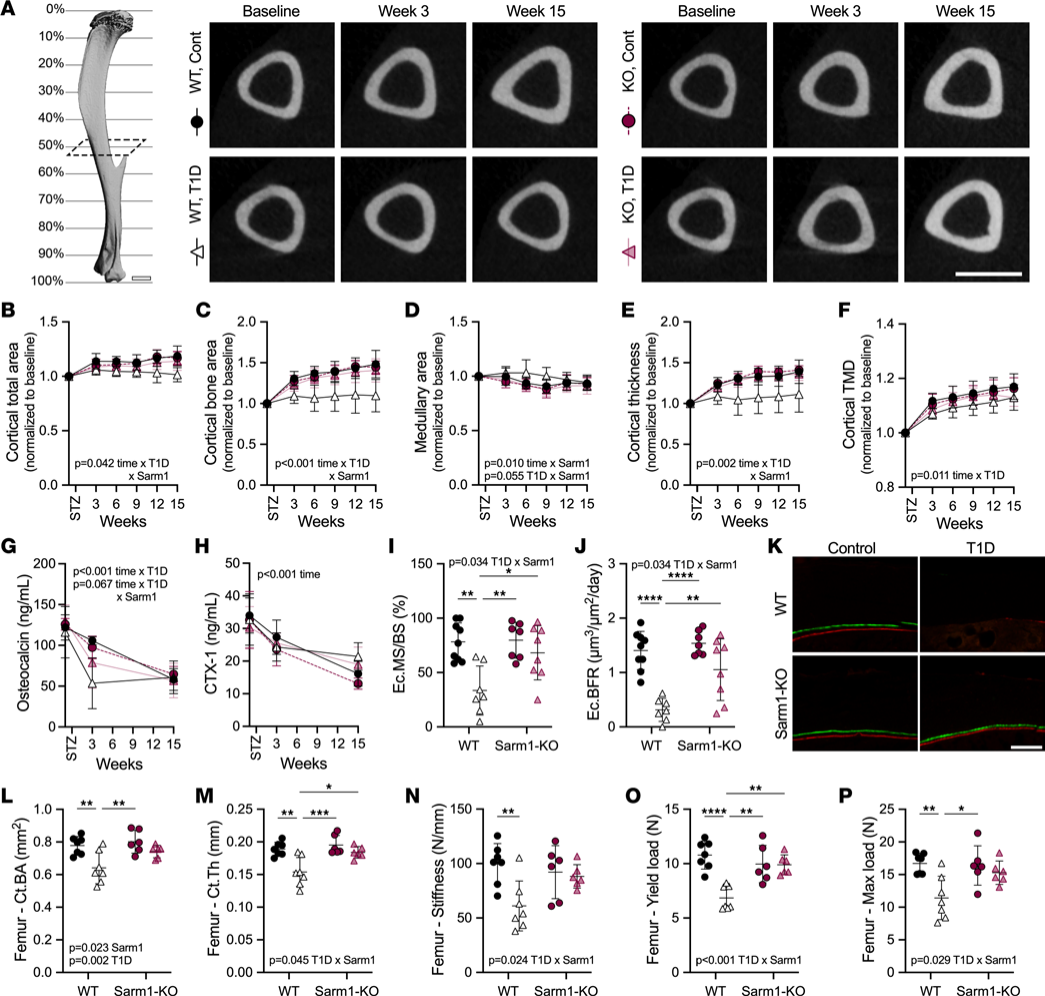
Female Sarm1^KO^ mice resist the onset of T1D bone disease. WT control and Sarm1^KO^ mice at 8 weeks of age were treated with STZ to induce T1D; controls received 0.9% saline. (**A**) Longitudinal in vivo microCT scans of the tibia were taken at the mid-diaphysis (~50% site). Representative 2D transaxial projections at baseline and 3 and 15 weeks of T1D. Scale bar = 1 mm. Tibia cortical total area (**B**), bone area (**C**), marrow area (**D**), cortical thickness (**E**), and cortical tissue mineral density (TMD, **F**) analyzed by in vivo microCT. (**G**–**H**) Plasma bone biomarkers osteocalcin (**G**) and type I collagen cross-linked C-telopeptide (CTX-1, **H**) measured by ELISA. (**I**–**K**) Endocortical mineralizing surface normalized to bone surface (**I**) and bone formation rate normalized to bone surface (**J**) analyzed from calcein/alizarin incorporation in the mid-diaphysis (~50% site) of the tibia at 3 weeks of T1D with representative micrographs of the medial surfaces (**K**). Scale bar = 100 μm. Femoral cortical bone area (**L**) and cortical thickness (**M**) analyzed by ex vivo microCT at 15-week endpoint. (**N**–**P**) Stiffness, yield load, and max load of the femur upon three-point bend test. (**B**–**H**) Three-way ANOVA/mixed model for genotype × T1D × time. (**B**–**F**) Each individual normalized to baseline. (**I**–**P**) Two-way ANOVA for genotype × T1D with Tukey’s multiple comparisons test. All data plotted as mean ± SD. *n* = 6–9/group. *P < 0.05, **P < 0.01, ***P < 0.001, ****P < 0.0001.

Unlike males, female WT and Sarm1^KO^ mice with T1D did not develop evidence of DPN within the 15-week time course of the study. Despite this, diabetic skeletal disease in WT females mirrored our results in males, including rapid suppression of cortical and trabecular bone accrual by longitudinal in vivo microCT beginning at 3 weeks of T1D (Figure 5, A–F, and Supplemental Figure 5), decreased circulating osteocalcin (–49% at week 3, Figure 5, G and H), suppression of bone formation by dynamic histomorphometry (–78% bone formation rate at week 3, Figure 5, I–K), and impaired morphology with decreased strength of the femur (–32% max load, Figure 5, L–P, and Supplemental Figure 6). This reinforces the finding that onset of diabetic skeletal disease is independent of DPN.

Unexpectedly, unlike WT mice, Sarm1^KO^ female animals were almost completely protected against the onset of the skeletal complications of T1D. This included total restoration of cortical bone accrual by longitudinal microCT (Figure 5, A–F), blunting of osteocalcin suppression (Figure 5, G and H), restoration of bone formation by dynamic histomorphometry (Figure 5, I–K), and improvement of femur bone morphology and strength to the level of the nondiabetic controls (Figure 5, L–P, and Supplemental Figure 6). Trabecular bone accrual was also sustained by *Sarm1* knockout up to approximately week 9 of T1D (Supplemental Figure 5). Together, these results reveal that global KO of Sarm1 in females facilitates the maintenance of osteoblast number and function even in a setting of severe T1D-associated metabolic disease and muscle atrophy.

**Figure 6:**
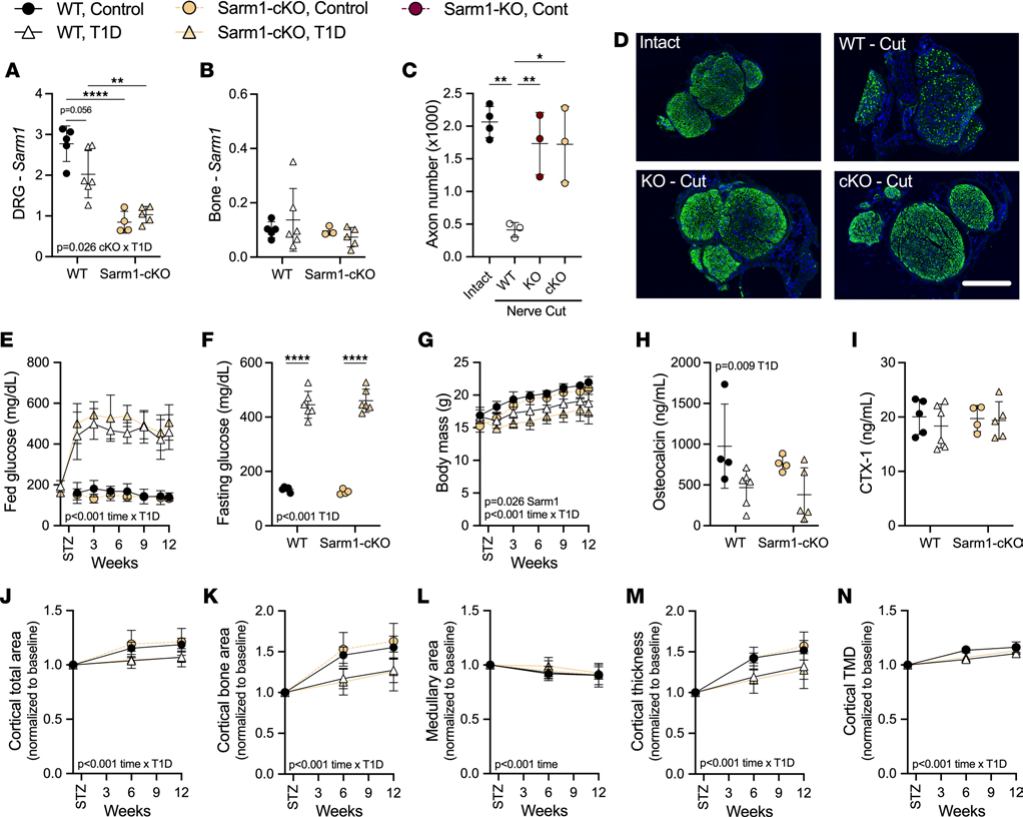
Neural conditional knockout of *Sarm1* does not prevent diabetic bone disease in female mice. Mice expressing the pan-neuronal promoter *Baf53b-Cre* were bred with *Sarm1^fl/fl^* animals to generate *Baf53b-Cre^+^ Sarm1^fl/fl^* experimental mice (cKO) and *Baf53b-Cre– Sarm1^fl/fl^* controls (WT). Female mice at 8 weeks of age were treated with STZ to induce T1D; controls received 0.9% saline. (**A** and **B**) Relative Sarm1 gene expression by qPCR in the neuroskeletal system including the DRG (**A**) and flushed bone (**B**) at 12-week endpoint. (**C** and **D**) Nerve fiber quantifications and representative images based on immunostaining for neurofilament H (green) in distal sciatic nerve sections 5 days after proximal sciatic nerve transection. Scale bar = 200 μm. (**E**) Longitudinal fed blood glucose. (**F**) Fasting blood glucose at 12 weeks. (**G**) Longitudinal body mass. (**H** and **I**) Plasma bone biomarkers osteocalcin (**H**) and type I collagen cross-linked C-telopeptide (CTX-1, **I**) measured by ELISA at week 12. (**J**–**N**) Tibia cortical total area (**J**), bone area (**K**), marrow area (**L**), cortical thickness (**M**), and cortical tissue mineral density (TMD, **N**) analyzed by in vivo microCT. (**A**–**C**, **F**, **H**, and **I**) Two-way ANOVA for genotype × T1D with Tukey’s multiple comparisons test. (**E**, **G**, **J**–**N**) Three-way ANOVA/mixed model for genotype × T1D × time. (**J**–**N**) Each individual normalized to baseline. All data plotted as mean ± SD. *n* = 3–6/group. *P < 0.05, **P < 0.01, ****P < 0.0001.

To determine if restoration of bone in global Sarm1^KO^ females was due to the suppression of the neural actions of SARM1, we generated a neural conditional *Sarm1-*knockout mouse. To achieve this, we bred mice expressing the established pan-neuronal promoter *Baf53b-Cre* with *Sarm1^fl/fl^* animals to generate *Baf53b-Cre^+^* *Sarm1^fl/fl^* experimental progeny (Sarm1^Baf53b-cKO^). The mice were initially validated in 2 ways. First, quantitative PCR (qPCR) verified decreased *Sarm1* expression in the DRGs in the Sarm1^Baf53b-cKO^ mice relative to controls, with no difference in expression in bone or bone marrow (Figure 6, A and B). Second, we found that Sarm1^Baf53b-cKO^ mice resisted axon degeneration after sciatic nerve cut to a level that was identical to global Sarm1^KO^ animals (Figure 6, C and D).

After validation, female Sarm1^Baf53b-cKO^ mice and controls were treated with STZ to induce severe T1D as in our other longitudinal cohorts (inclusion criteria fasting glucose > 300 mg/dL). As before, hyperglycemia and reduced body mass were present in mice with T1D, independent of genotype (Figure 6, E–G). However, unlike global Sarm1^KO^ females, conditional Sarm1^Baf53b-cKO^ females did not display evidence of bone protection. Specifically, osteocalcin was similarly reduced in mice with T1D (–52% WT versus –50% cKO), with no noted alterations in CTX-1 (Figure 6, H and I). In addition, T1D-associated suppression of cortical bone accrual was observed by longitudinal microCT regardless of genotype (Figure 6, J–N). This shows that suppression of the neural actions of SARM1 is not sufficient to recapitulate the bone protection phenotype observed in the global Sarm1^KO^ female mice.

### Sarm1 is highly expressed in neurons but may also be present at low levels in skeletal and hematopoietic cells.

The neural expression and actions of SARM1 are well established. Consistent with this, we found high *Sarm1* expression in neural tissues, such as the DRGs (Figure 6A and Supplemental Figure 7A). By contrast, *Sarm1* expression by qPCR was >95% lower in bone and bone marrow (Figure 6B and Supplemental Figure 7A). Though low, this expression was still significantly reduced in Sarm1^KO^ bone relative to WT bone, with trending reductions also noted in Sarm1^KO^ bone marrow (*P* < 0.067). We also explored SARM1 expression at the protein level using anti-FLAG immunostaining of mouse tissues expressing 2× FLAG-tagged SARM1 (Supplemental Figure 7B). Direct immunostaining with anti-SARM1 was not possible because of poor specificity of available commercial antibodies. However, anti-FLAG immunostaining detected robust expression of FLAG-SARM1 in both DRG sensory neurons and sympathetic neurons in the aorticorenal ganglia (Supplemental Figure 7B). By contrast, FLAG-SARM1 was not detected in bone or its associated tissues using paired development procedures (analyzed cross sections of mouse bone included osteoblasts, osteoclasts, osteocytes, periosteum, adipocytes, stroma, vasculature, and bone marrow cells). This does not rule out low-level expression of SARM1 in these cells, but it reinforces prior data showing high relative expression of SARM1 in neurons (39, 40). Last, we analyzed *Sarm1* expression by qPCR in cultured bone marrow stromal cells (BMSCs), pre-osteoblasts, bone marrow–derived macrophages (BMMs) with/without LPS, and pre-osteoclasts with/without LPS from WT and Sarm1^KO^ mice (Supplemental Figure 7, C–E). Comparable to the bulk tissue analyses, we found that low-level *Sarm1* expression was significantly reduced by Sarm1^KO^ in all cell types and conditions. Future generation of extraneural *Sarm1*-cKO models will be needed to define the biological relevance of this low-level signal in vivo and to localize the source of the Sarm1^KO^-dependent skeletal protection in settings of T1D.

### Bone protection in Sarm1^KO^ females highlights critical biological processes for mitigating diabetic bone disease.

To leverage our finding in the global Sarm1^KO^ females, we performed RNA-Seq with pattern analysis to identify differentially expressed genes (DEGs) that were altered in WT with T1D but unchanged in Sarm1^KO^. We hypothesize that these “rescued” genes represent those that are associated with the unique maintenance of bone health in the Sarm1^KO^ females. Furthermore, this may identify novel mechanisms by which global loss of *Sarm1* promotes bone formation even in settings of severe metabolic disease and muscle atrophy.

**Figure 7:**
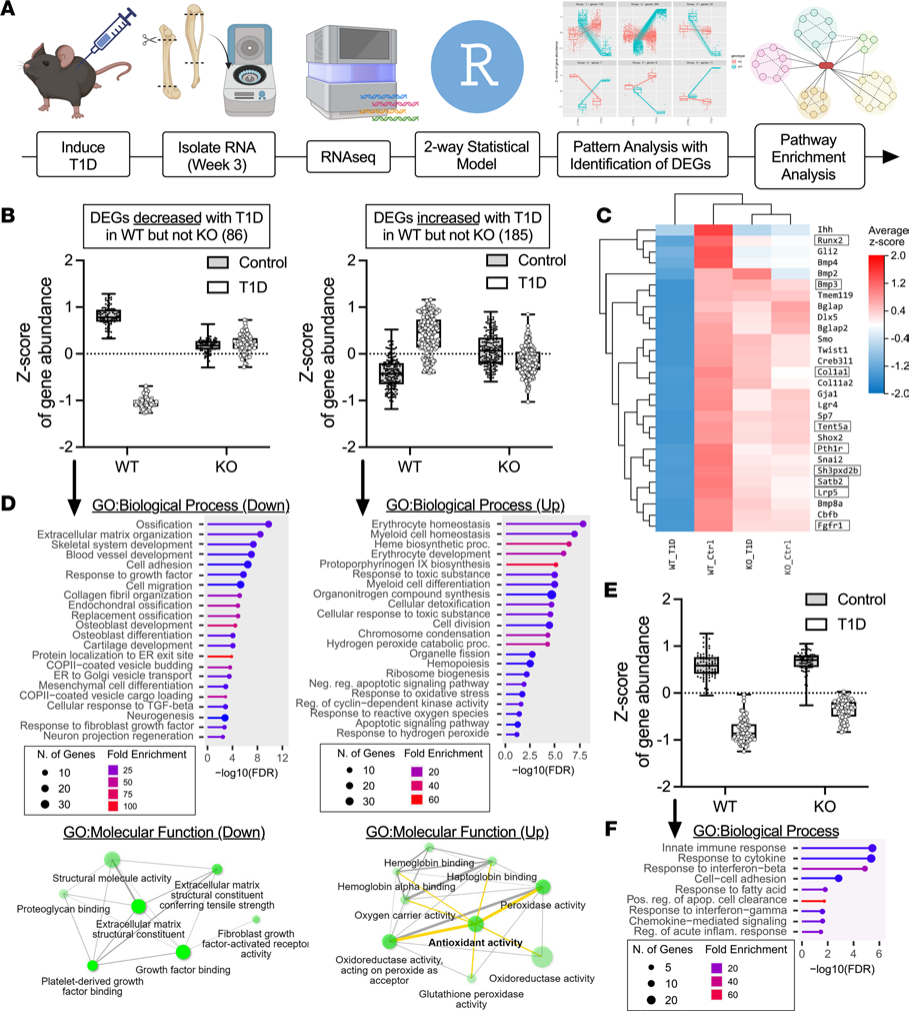
RNA-Seq with pattern analysis highlights critical biological processes for mitigating diabetic bone disease in Sarm1^KO^ females. WT control and Sarm1^KO^ female mice at 8-weeks of age were treated with STZ to induce T1D; controls received 0.9% saline. RNA extraction and analysis was performed after 3 weeks of confirmed T1D (age 11 weeks). (**A**) RNA preparation, RNA-Seq, and pattern analysis pipeline. RNA-Seq with pattern analysis was used to identify differentially expressed genes (DEGs) that were altered in WT with T1D but unchanged in female Sarm1^KO^ samples (“rescued”). Based on the significance of the interaction term, we identified 6 primary clusters (actual image above “pattern analysis with identification of DEGs”). (**B**) The subset of DEGs with log_2_ fold-change >|0.35| (>1.27-fold) was retained for pathway enrichment analysis (86 down and 185 up; full details in Supplemental Table 3). Each dot represents an individual gene, graphed as the *z* score of gene abundance. (**C**) Heatmap of osteoblast differentiation-related genes. Boxes = identified by pattern analysis. (**D**) ShinyGO pathway enrichment analysis based on the DEGs in **B**. Full results available in Supplemental Table 4. (**E**) Subset of “dispensable” DEGs that were altered with T1D, regardless of genotype (80 down presented as *z* score of gene abundance, and 12 up not shown; full list available in Supplemental Table 5. (**F**) ShinyGO pathway enrichment analysis based on the DEGs in **E**. *n* = 5–6/group. Panel **A** created with BioRender.

This analysis was performed using RNA extracted from marrow-depleted femur and tibia bone from WT and global Sarm1^KO^ females after 3 weeks of T1D, at 11 weeks of age (Figure 7A). This time point was selected to capture gene changes early in the course of disease, around the time that changes in bone by microCT began to emerge. To isolate the effect of genotype on changes in DEGs with T1D, we fit a full 2-way statistical model for pattern analysis (diabetes, genotype, and diabetes × genotype). Statistical pattern analysis based on the significance of the interaction term identified 6 primary clusters (Figure 7A). Biologically, this represented 2 groups with 173 downregulated DEGs (clusters 1, 3, 5) and 316 upregulated DEGs (clusters 2, 4, 6) that were altered in WT T1D but remained unchanged with T1D in Sarm1^KO^ mice (Supplemental Table 3). The subset of DEGs with log_2_ fold-change >|0.35| (>1.27-fold) was retained for pathway enrichment analysis (86 down and 185 up; Figure 7B and Supplemental Table 3).

Consistent with restoration of bone health in our other skeletal analyses, DEGs that were downregulated in WT mice with T1D and “rescued” by Sarm1^KO^ included key genes related to osteoblast differentiation and function, such as runt related transcription factor 2 (*Runx2*), FGF receptor 2 (*Fgfr2*), parathyroid hormone 1 receptor (*Pth1r*), and collagen type I alpha 1 (*Col1a1*), among others (Figure 7C). Analysis of downregulated DEGs also highlighted deficiencies in bone formation–related biologic processes including ossification, angiogenesis, collagen synthesis, and extracellular matrix (ECM) organization (Figure 7D). Disrupted pathways included integrin and ECM/receptor interactions, PI3K/Akt signaling, PDGF binding, regulation of IGF transport, and FGFR activity (Supplemental Table 4). Gene-level evidence for deficiencies in protein synthesis, packaging, and secretion in COPII-coated vesicles were also noted. Overall, this demonstrated that female Sarm1^KO^ mice were protected against T1D-associated declines in genes related to osteoblast function and bone formation.

Conversely, upregulated DEGs in WT T1D that were unchanged with diabetes in Sarm1^KO^ mice centered on pathways involved with catabolism of reactive oxidative species, erythrocyte and myeloid cell development, cell division and apoptosis, and responses to oxidative stress (Figure 7D and Supplemental Table 5). This analysis reveals that cells within the WT T1D skeleton exhibit high levels of adaptive responses to oxidative stress. By contrast, genetic markers of oxidative stress responses were not present with T1D in the Sarm1^KO^ bones.

Last, we wanted to identify “dispensable” pathways that were altered with T1D, regardless of genotype. To do this, we first identified genes with a log_2_ fold-change >|0.35| (>1.27-fold) with T1D in both WT and Sarm1^KO^ female mice relative to controls (643 genes). We then used a 2-way biostatistical model to calculate *P* values for genotype, diabetes, and diabetes × genotype. Genes with an interaction term of *P* < 0.05 were removed. Of the 562 remaining genes, those with *P* < 0.01 for the individual term diabetes were retained for downstream analysis (92 total). This subanalysis pinpointed 80 DEGs that were downregulated and 12 DEGs that were upregulated with T1D in both WT and Sarm1^KO^ mice (Figure 7E and Supplemental Table 6). The most highly expressed upregulated genes were carbonic anhydrase 1 (*Car1*), adipsin (*Cfd*), and tachykinin 2 (*Tac2*). However, there were not enough genes in the upregulated category to perform pathway analysis. Pathway enrichment analysis of the 80 downregulated DEGs revealed shared changes in the innate immune response, cytokine response, cell-cell adhesion, chemokine signaling, and acute inflammation in both WT and Sarm1^KO^ female mice with T1D, despite rescue of bone in Sarm1^KO^ animals (Figure 7F and Supplemental Table 7). This included changes in the predicted response to IFN-β and IFN-γ. The “dispensable” nature of this pathway for the pathogenesis of diabetic bone disease aligns with prior work showing that IFN-γ deficiency does not prevent bone loss in T1D (41).

### Human GWAS identify SARM1 as a candidate causal gene for bone mineral density.

Our work in mice reveals that knockout of *Sarm1* promotes bone health in females with T1D, in addition to providing established protection from the onset of DPN. However, the relevance of this finding to humans remains unclear. To begin to understand this, we interrogated the known relationship between *SARM1* gene variants and bone health using the Open Targets database version 22.10 (42). Five GWAS considering over 0.5 million individuals of mixed age and sex have identified variants near *SARM1* as associated with bone mineral density based on data from the UK Biobank and the Genetic Factors for Osteoporosis Consortium. This includes 1 intergenic variant and 1 upstream variant, both of which are associated with potential for changes in *SARM1* gene splicing and product level (43–47) (Supplemental Table 8). This further supports the consideration of bone quality and bone density as potential outcomes for future clinical trials of SARM1 inhibitors in settings of diabetes and beyond.

## Discussion

### Rapid onset and progression of bone disease in T1D is independent of DPN.

In this study, we used mouse models of T1D and SARM1-dependent neuropathy to isolate the functional relationship between DPN and the onset and progression of diabetic skeletal disease. This has been an important open question in the field based on clinical literature showing associations between neuropathy and bone health (11–13). We found that suppression of osteoblast function in T1D occurs rapidly in both males and females. This included an 80% to 90% reduction of bone formation within only 3 weeks of the onset of severe disease that affected both cortical and trabecular bone accrual. By contrast, the onset of functional DPN was more gradual in males (evident by weeks 6 to 9) and was not detected at all in females during the 15-week duration of the study. This mirrors prior studies showing that functional neuropathy in mice occurs between 6 and 36 weeks of T1D (38). Beyond this, we found that prevention of SARM1-dependent DPN in males or the absence of DPN in WT females was not sufficient to mitigate the onset or progression of diabetic skeletal disease. This reveals that the rapid, detrimental effects of T1D on osteoblast function, bone morphology, and bone strength are not dependent on DPN.

The independent onset of diabetes-associated skeletal disease is an important point to clarify for clinical practitioners focused on the care of young persons with T1D. Bone disease is insidious and does not cause outward symptoms but can lead to fracture, loss of function, and early mortality later in life (48–50). While basic neuropathy screening is an established part of the pediatric T1D examination (51), bone health studies are not. Our work shows that screens for other complications such as DPN cannot be used as a reliable surrogate for bone. Indeed, by the time DPN is identified, substantial changes to the underlying bone have likely already occurred. Emerging clinical research on bone health in adolescents with T1D also suggests that skeletal complications need to be managed early in the course of disease (48, 52–56). Overall, this will require the development and validation of suitable screening tools for pediatric populations, including fracture history and circulating bone turnover biomarkers, and application of tools capable of detecting early shifts in bone density and microarchitecture, such as DXA and HRpQCT, respectively.

### Though not a causative factor, progressive DPN is expected to exacerbate fracture risk with age.

This study clarifies that the initial onset and progression of diabetic skeletal disease are independent of DPN. However, it is important to point out that worsening DPN that affects the motor system may amplify preexisting skeletal disease because of progressive muscle atrophy, a feature that is inextricably linked to bone health. In addition, progressive motor, autonomic, and sensory changes in DPN can modify aspects of balance and proprioception, leading to increased risks for both falls and fracture (reviewed in ref. 11).

### Global Sarm1^KO^ protects bone health in females, but not males, with T1D.

**Figure 8:**
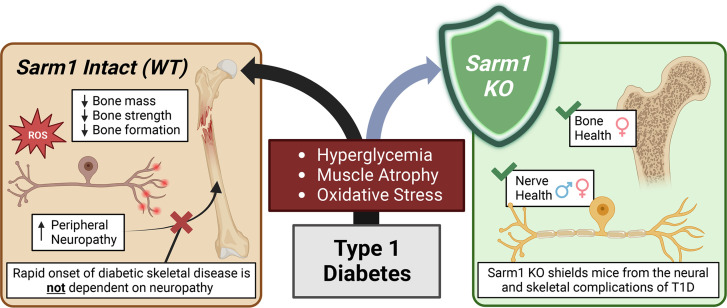
*Sarm1* knockout shields female mice from the onset of diabetic bone disease: model figure. T1D is characterized by the immune-mediated destruction of pancreatic β cells that culminates in insulin deficiency and hyperglycemia. Metabolic derangements in T1D contribute to the development of complications, including DPN and diabetic skeletal disease. In this study, we found that the onset of skeletal disease occurs rapidly in both male and female mice with T1D. This was completely independent of DPN. In addition, consistent with prior reports, we found that knockout of *Sarm1* prevents the onset of DPN. Last, we believe we have identified *Sarm1* knockout as a novel mechanism to shield female mice from the deleterious skeletal effects of T1D. Rescue of bone health by global *Sarm1* knockout was due to sustained osteoblast function with abrogation of local oxidative stress responses. This was independent of the neural actions of SARM1, as beneficial effects on bone were lost with neural conditional *Sarm1* knockout. Figure created with BioRender.

A key finding of our work is the sex-specific skeletal protection afforded by Sarm1^KO^, providing a shield against osteoblast suppression in female mice even in a setting of severe T1D-associated metabolic disease and muscle atrophy (Figure 8). To begin to understand this result, we can first consider the T1D phenotypes across both males and females. Females are often thought to be unsuitable for studies of STZ-induced T1D because of poor conversion. However, by refining our induction protocol, we were able to overcome this limitation. This facilitated the induction of severe T1D-associated metabolic disease in both sexes (inclusion criterion of sustained fasting glucose > 300 mg/dL). Though all mice in the study were classified as severe, female mice still had consistently lower levels of circulating glucose than males (443 ± 53 mg/dL versus 541 ± 43 mg/dL, *P* < 0.001). We also observed differences in the onset of neuropathy. Specifically, females did not develop functional neuropathy within the 15-week time course of the study whereas this presented by weeks 6 to 9 in WT males. The accrual of body mass from week 0 to week 15 of disease was also relatively preserved in the T1D females (+14%) versus males (–7%). This is in direct contrast to the onset of diabetic skeletal disease, which occurred rapidly and to the same extent in both WT male and female mice with T1D. Decreases in the cross-sectional area of the muscle fibers were also more pronounced in females with T1D, independent of genotype.

The mechanism for the sex-specific rescue of bone by Sarm1^KO^ remains unknown and may possibly be related to differences in circulating hormones and pathways between males and females. Differential effects could also be related to the differences in hyperglycemia. For example, the sustained circulating glucose in males, approximately 22% higher than females, may have overwhelmed the protection of osteoblasts afforded by loss of *Sarm1*. If true, we may expect SARM1 inhibitors to perform well in humans regardless of sex, as diabetic skeletal disease emerges at much lower levels of glycemia in humans (48, 52–56).

### Cellular origins of skeletal protection in Sarm1^KO^ mice.

To isolate the cellular origin of skeletal protection in global Sarm1^KO^ mice, we generated a *Sarm1^fl/fl^* mouse, followed by breeding to *Baf53b-Cre*, to generate a neural conditional *Sarm1*-knockout model. This approach was based on the high expression of *Sarm1* in the nervous system and the well-established role of SARM1 as the central executioner of axon death, including in settings of DPN (31, 32). However, we found that neural *Sarm1* knockout did not rescue bone in T1D. This reinforces the independence of diabetic bone disease from DPN and suggests that the benefits of *Sarm1* knockout for bone occur via *Sarm1*-expressing cells outside of the nervous system, possibly directly via a bone cell-autonomous mechanism or indirectly via humoral signaling from another organ. At this point, it becomes difficult to pinpoint the origin of the signal as all cells that we have tested to date, including whole bone and bone marrow, and cultured BMSCs, BMMs, and pre-osteoclasts, show evidence of very low *Sarm1* expression. This is consistent with prior identification of low-level *Sarm1* expression in BMMs (57) and in diverse tissues, including liver, kidney, and beyond (57, 58). For example, a prior study addressing the controversial expression of *Sarm1* in BMMs showed that FLAG-tagged SARM1 was not detected by immunoblotting (57). However, SARM1 expression in BMMs was clearly detected by more sensitive techniques, including anti-FLAG immunoprecipitation and qPCR (57). Future resolution of this point will require the generation and careful assessment of additional *Sarm1* conditional knockout models in addition to translational and clinical studies of SARM1 function in human cells and tissues.

### Improved bone health in Sarm1^KO^ mice is linked to reduced activation of oxidative stress pathways.

There is recent controversy regarding certain inflammatory gene changes between WT and Sarm1^KO^ mice because of potential for limited carryover of 129/SvJ genetic material on chromosome 11 (57, 59). In our model (backcrossed extensively to C57BL/6N mice since 2013), we found that gene-level changes in inflammatory pathways, chemokine activity, and cytokine production were comparable in WT and Sarm1^KO^ diabetic mice, suggesting that modulation of these pathways was not responsible for improved bone health. Instead, our genetic analyses show that restoration of osteoblast function and bone formation in Sarm1^KO^ females was linked to reduced oxidative stress and local oxidative stress responses. In neurons, *Sarm1* knockout functions downstream of mitochondrial changes to protect cells from death due to prolonged exposure to ROS (60). By contrast, emerging in vitro evidence suggests that SARM1 clustering contributes directly to the induction of mitochondrial depolarization in immune cells (61, 62). Thus, in addition to downstream protection from ROS, knockout of *Sarm1* may modulate mitochondrial function with potential for modification of ROS production by some cell types (30, 62). Overall, our results identify the physiologic modulation of oxidative stress pathways and responses by *Sarm1* knockout as the most likely mechanism underlying the rescue of bone.

### Sarm1 inhibitors as a clinical management strategy for neuropathy and skeletal disease in T1D.

Diabetes and hyperglycemia increase the systemic accumulation of ROS (4, 63). When produced in excess, ROS can damage mitochondria and DNA, increase lipid and protein oxidation, and activate damage repair responses and apoptosis (64). Overall, this prevents cells and tissues from carrying out their intended functions. The modulation of oxidative stress represents a key therapeutic target for management of diabetic complications across systems, including in nerve and bone (63). Unfortunately, antioxidant therapies to date have mostly failed to provide clinical benefit. This conflict is explained by the recent identification of many positive, evolutionarily conserved roles for ROS in both signaling and metabolism. Given this, newer therapies are focused on modulating specific sources and targets of ROS, while leaving essential physiologic ROS pathways intact (63).

SARM1 is an enzyme and metabolic biosensor that is responsive to increased ROS (60). Emerging evidence suggests that SARM1 may also regulate mitochondrial function and ROS production in some cell types (30, 61, 62). SARM1 is generally inactive in healthy cells and is only activated in response to inflammation, injury, and oxidative stress (18, 22, 23, 26, 60, 65). Based on our results and previous observations (31, 39), this means that global knockout of *Sarm1* has minimal effects on development or homeostasis of healthy mice. Targeting SARM1 activation therapeutically thus has great potential to protect *Sarm1*-expressing cells from damage due to oxidative stress and other insults. Current efforts are focused on the development of SARM1 inhibitors for the prevention and treatment of neurologic disease (33, 34). Our work suggests that these benefits may also extend to bone, identifying SARM1 as a shared target to promote diabetic neuroskeletal health. We suspect that these therapies may be particularly useful for adolescents or other patients with T1D who have difficulty maintaining optimal glycemic control (66), providing added protection against the onset and progression of diabetic complications during these challenging times.

### Conclusion.

The onset of skeletal disease occurs rapidly in both male and female mice with T1D. This is completely independent of DPN, which develops later in the course of disease. Consistent with prior reports, knockout of *Sarm1* prevents the onset of DPN. In addition, knockout of *Sarm1* shields female mice from the deleterious skeletal effects of T1D. Rescue of bone health by *Sarm1* knockout is due to sustained osteoblast function and abrogated local oxidative stress responses, even in settings of severe metabolic disease. Clinically, this reveals that bone is likely one of the earliest organ systems to be affected by T1D and that screens for other complications, such as clinical neuropathy, cannot be used as a reliable surrogate. In addition, this work supports the exploration of clinical SARM1 inhibition as a promising therapeutic strategy to promote neuroskeletal health in settings of diabetes and metabolic disease.

## Methods

### Animals

Experiments were approved by the Animal Studies Committee at Washington University. Mice were group-housed in a specific pathogen–free facility at 22°C–23°C on a 12-hour light/ 12-hour dark cycle and fed standard chow (LabDiet PicoLab, 5053). Global *S*arm1^KO^ mice have been described previously and are maintained on a C57BL/6NTac background (39). WT control and Sarm1^KO^ littermate mice were derived from heterozygous breeding pairs. WT age-, sex-, and background-matched C57BL/6NTac mice were also ordered from Taconic. Two new models were generated using CRISPR electroporation of 1-cell embryos by the Genome Engineering & Stem Cell Center at Washington University. This included a mouse model expressing a FLAG-tagged SARM1 (Sarm1^2×FLAG^) and a mouse with *loxP* sites flanking exon 2 of *Sarm1* (*Sarm1^fl/fl^*) (see Supplemental Methods).

To generate mice with conditional knockout of S*arm1* in the nervous system, *Sarm1^fl/fl^* mice were bred with mice expressing *Baf53b-Cre* (The Jackson Laboratory, strain 027826). Baf53b-Cre has previously demonstrated efficient *loxP* excision in both central and peripheral neurons, including those targeting the skeleton (67). *Baf53b-Cre/^+^ Sarm1^fl/fl^* males were crossed with *Sarm1^fl/fl^* females to generate Cre^+^ conditional knockout mice (Sarm1^Baf53b-cKO^) and associated Cre^–^ controls.

### Induction of diabetes and glucose monitoring

Mice were fasted overnight prior to i.p. injection with 100 mg/kg STZ (MilliporeSigma S0130) made fresh in saline and used within 5 minutes of reconstitution. Food was returned to the cage 1 hour after STZ injection. Control mice received saline only. Fasting, injection, and refeeding were repeated on day 2. Blood glucose was measured on day 3 with a standard glucometer by using a lancet to obtain a drop of blood from the vein at the tip of the tail (Bayer Contour). STZ was administered again on day 4 following the same procedure if fed blood glucose levels did not reach 300 mg/dL. Glucose was monitored throughout the course of the experiment as described above. When indicated, fasted glucose was obtained following a 6-hour fast on aspen bedding.

### GTT and glucose-stimulated insulin secretion

Mice were fasted on aspen bedding for 6-hours. To measure glucose tolerance, 2 mg/kg dextrose was administered by i.p. injection, and blood glucose was measured at indicated time intervals from the tail with a standard glucometer. For glucose-stimulated insulin secretion, whole blood was collected from the lateral tail vein 30 minutes after 2 mg/kg dextrose injection. Plasma insulin was measured by Mouse Insulin Immunoassay (Singulex).

### Body composition

Body composition was measured by DXA (initial severity study) or EchoMRI (longitudinal and cKO studies). For DXA, mice were anesthetized with ketamine/xylazine and imaged using a Faxitron using manufacturer-recommended settings to quantify whole-body bone mineral content, fat mass, and lean mass (Faxitron Bioptics, LLC). For EchoMRI, animals were placed into a thin-walled plastic cylinder with an insert added to limit movement prior to scanning with a low-intensity (0.05 Tesla) electromagnetic field to measure fat, lean mass, free water, and total body water (EchoMRI-900, Echo Medical Systems).

### Neurobehavioral and electrophysiological assessments

#### Rotarod.

Mice were placed in individual compartments of a Rotamex-rotarod and acclimated to walking on the rotating bar for 5 minutes. Bar rotation then gradually accelerated until the mouse fell from the bar into the collection chamber, and the retention time was recorded (5 trials/mouse).

#### Inverted screen.

Mice were individually placed on wire mesh, then inverted for 120 seconds, or until their grip released (3 trials/mouse).

#### Ramping hotplate.

Mice were acclimated to a hotplate chamber for 5 minutes at 30°C (BioSeb, BIO-CHP). The temperature was gradually increased until the mouse exhibited signs of sensitivity (rear paw licking, jumping, or elevation of the rear limb).

#### Nerve conduction.

Mice were anesthetized using 2% isoflurane. Platinum subdermal recording electrodes (30-gauge, 10 mm length; Natus Neurology) were placed between the fourth and fifth digits of the rear paw, at the ankle near the sural nerve, and/or at the sciatic notch. Ground and reference electrodes were placed subcutaneously at the rear flank. Supramaximal square wave pulses of 0.1 ms duration were applied using a Viking Quest electromyography machine (Nicolet) to obtain evoked compound nerve action potentials. Responses were recorded from stimulation at both the ankle and the sciatic notch. Amplitude was measured from baseline to the first peak; conduction velocity was calculated as a function of distal latency and the distance between stimulating and recording electrodes.

### Foot pad nerve assessment

At the time of dissection, the plantar surface of the hind paw was removed and processed as described previously to analyze intraepidermal nerve fibers (29) (additional details in Supplemental Methods).

### Plasma biomarkers

Mice were fasted on aspen bedding for 6 hours prior to anesthesia with 2% isoflurane and topical proparacaine. Whole blood was collected retro-orbitally with heparin-coated glass capillary tubes. Blood was immediately extruded into EDTA-coated sample tubes, incubated on ice for 20 minutes, and centrifuged at 3,000*g* for 15 minutes at 4°C. Plasma supernatant was stored at –80°C until use. Plasma biomarkers were assessed with the Quidel Immutopics Mouse Osteocalcin ELISA Kit (catalog 60-1305), ImmunoDiagnoisticSystems RatLaps (CTX-I) EIA (catalog AC-06F1), and R&D Systems Quantikine ELISA Mouse/Rat IGF-1 kit (catalog MG100) per the manufacturers’ instructions. Plasma was diluted 1:4 for osteocalcin and 1:500 for IGF-1 per manufacturers’ recommendations.

### Dynamic histomorphometry

Calcium-binding fluorochromes were injected subcutaneously to label mineralizing bone in vivo at week 3 of T1D. Specifically, 10 mg/kg calcein (MilliporeSigma C0875) was administered 10 days prior to the 3-week endpoint, and 30 mg/kg alizarin (MilliporeSigma A3882) was administered 3 days prior to endpoint (details in Supplemental Methods).

### Bone morphology and biomechanical testing

For ex vivo micro-CT, bone lengths were measured using a digital caliper (Mitutoyo) before embedding in 2% agarose and scanning at 20 μm resolution on a Scanco μCT-40 (Scanco Medical). For in vivo micro-CT, mice were anesthetized with 1% to 2% isoflurane and placed into the scanning bed of a VivaCT40 (Scanco Medical). The right limb was scanned at 70 kVp, 114 uA, 8 W, within a 21.5 mm field of view at 21 μm voxel resolution. The cortical bone scan region was 1 mm proximal to the tibiofibular junction, and the trabecular scan region was distal to the metaphyseal growth plate. The resolution and approximately 2 mm region size were selected to minimize the radiation dose (scan time of 6.7 minutes per region). Analysis was performed using Scanco software and was restricted to 420 μm of the mid-diaphyseal cortical bone and 1 mm of the trabecular bone in the metaphysis immediately distal to the growth plate using a common threshold across samples.

For biomechanical testing, unfixed femurs were wrapped in PBS-soaked gauze and stored at −20°C. For testing, femurs were thawed in PBS and cortical bone parameters measured using microCT as above. Biomechanical 3-point bend testing was then carried out at room temperature using a servohydraulic testing machine (8841 Dynamite, Instron). Femurs were positioned on 2 supports 7 mm apart, and the central loading point was at the mid-diaphysis. Displacement was applied transverse to the long axis of the bone at a rate of 0.01 mm/s until failure. Force-displacement data were recorded at 60 Hz and analyzed to determine measures of stiffness and whole bone strength.

### Muscle fiber cross-sectional analysis

Gastrocnemius, soleus, and plantaris muscles were mounted in tragacanth gum and flash-frozen together in liquid nitrogen–cooled isopentane. Sections of 10 μm were cut from the midbelly on a Leica CM1950 cryostat. Sections were immunostained with type 1, type 2a, and type 2b myosin heavy chain isoforms (Developmental Studies Hybridoma Bank BA-F8, SC-71, and BF-F3) and laminin (Abcam 1575). Representative original magnification, 20×, images were acquired for analysis of cross-sectional area with a semiautomated ImageJ (NIH) macro as previously described (68).

### Sciatic neurectomy and nerve immunohistochemistry

Mice at 10 to 12 weeks of age were anesthetized with 1%–2% isoflurane prior to transection of the left proximal sciatic nerve. Five days after the transection, mice were anesthetized with ketamine/xylazine cocktail and perfused through the left ventricle of the heart with 10 mL PBS followed by 10 mL 10% neutral buffered formalin (NBF). A segment of the sciatic nerve was dissected from the left leg distal to the transection and from a similar position from the intact nerve from the right leg. Nerves were postfixed in 10% NBF overnight, washed in deionized H_2_O for 2 hours, and cryoprotected overnight in 30% sucrose prior to embedding in OCT mounting media (Thermo Fisher Scientific 23-730-571). Embedded nerves were cut at 10 μm on a cryostat (Leica) and mounted on Colorfrost Plus glass slides (Thermo Fisher Scientific 12-550-18). Sections were immunostained as previously described (67) with chicken anti-neurofilament, heavy chain (1:100; MilliporeSigma AB5539). Fluorescence-conjugated secondary antibody was used for visualization of axons (711-546-152; Cy3 anti-chicken, Jackson ImmunoResearch) along with DAPI to visualize nuclei (MilliporeSigma D9542). Tiled images of stained sections were taken with a 10× objective using a Nikon spinning disk confocal microscope (μm/pixel = 0.650, overlap = 10%). Images were analyzed in ImageJ/FIJI. Neurofilament staining was thresholded to select all positively stained axons in the largest bundle among the 3 in the distal sciatic nerve sections. Images were water-shed and particles larger than 25 pixels were excluded to eliminate nonaxonal components. Axon numbers were counted using the analyze particles function.

### Cell culture

Bone marrow cells were collected from the tibia and femur of WT and Sarm1^KO^ mice at 8–12 weeks of age by brief centrifugation at 1,000*g* for 1 minute at room temperature. BMSCs were isolated using MesenCult media (StemCell Technologies 05513; base media with 1% Pen/Strep, 1% l-glutamine, MesenPure additive, and 10% serum) in 10 cm plates at 37°C, 5.0% CO_2_, until reaching 70% to 80% confluence around day 5. BMSCs were passaged using 0.25% trypsin and expanded with MesenCult expansion media. At P2–P3, BMSCs were plated onto 12-well plates at a density of approximately 15,000 cells/cm^2^ in 1 mL media, feeding every 3 to 4 days. At 80% confluence around days 4 to 5, BMSCs were treated with MesenCult Osteogenic Stimulatory media with 1% Pen/Strep and 1% l-glutamine (day 0). RNA for *Sarm1* expression was collected at day 0, day 3 (pre-osteoblast), and day 11 (osteoblast). For BMM isolation, bone marrow cells were suspended in α-MEM supplemented with 1:10 CMG 14-12 supernatant (contains M-CSF, provided by the Veis Lab, Division of Bone and Mineral Diseases, Washington University in St. Louis) (49), 10% FBS, 1% Pen/Strep, and 1% l-glutamine. Cells were plated at a density of 2 bones (femur or tibia) per untreated p150 plate and incubated at 37°C, 5.0% CO_2_, for 5–8 days to reach 80% confluence. For osteoclastogenesis, BMMs were lifted by scraping and suspended in BMM maintenance media as above or osteoclastogenic media (α-MEM, 1:50 CMG, 100 ng/mL RANKL, 10% FBS, 1% Pen/Strep, and 1% l-glutamine) prior to plating into cell culture–treated 6-well plates at a density of approximately 25,000 cells/cm^2^. After differentiation, primary BMMs and day 3 pre-osteoclasts were stimulated with 0–100 ng/mL of LPS (provided by the Faccio Lab, Department of Orthopaedics, Washington University in St. Louis) for 4 hours prior to RNA collection for *Sarm1* expression.

### RNA preparation and qPCR

Cells or tissues were harvested and immediately homogenized in TRIzol reagent (Thermo Fisher Scientific 15596-026). For bone samples, tibia and femur from one hind limb were cleaned with gauze to remove soft tissue, epiphyses were removed to expose marrow cavity, and bones were placed into a 0.5 mL tube with an 18-gauge needle hole in the bottom nested in a 1.5 mL tube. Marrow was separated by centrifugation at 1,000*g* for 1 minute. Marrow was digested in 1 mL TRIzol by pulling through a 22-gauge needle. The marrow-depleted bone was placed into a RINO 1.5 mL Screw Cap tube (MidSci) with stainless steel beads and 1 mL TRIzol. Bones were snipped with dissection scissors into fragments before bullet homogenization for 5 minutes. DRGs were dissected from L1–L6, minced with a blade, and further homogenized in 500 μL TRIzol using a needle bevel against the side of the tube. Samples in TRIzol were incubated at room temperature for 5 minutes for complete digestion before extraction with chloroform and PureLink RNA Mini Kit (Invitrogen 12183025) according to manufacturer instructions. RNA was eluted in 30 μL RNase-free water, and the concentration was determined using NanoDrop. RNA samples were stored at –80°C until use.

For *Sarm1* qPCR, an equal amount of RNA (generally 400–800 ng/reaction) was used for reverse transcription with SuperScript IV VILO Master Mix with ezDNase (Invitrogen 11766050). Quantitative real-time PCR was performed with qPCRBIO SyGreen Mix Lo-Rox (PCR Biosystems PB20.11-51) according to manufacturer instructions. Primers were as follows: *Sarm1* (forward: CTTCGCCAGCTACGCTACTTGC, reverse: TTATCACGGGGTCCATCATCGT), cyclophillin A (*CycloA*; forward: CACCGTGTTCTTCGACATCA, reverse: CAGTGCTCAGACTCGAAAGT), and TATA-box-binding-protein (*Tbp*, forward: ACCTTATGCTCAGGGCTTGG, reverse: GCCGTAAGGCATCATGAC). Reactions were performed in 96-well plates (MicroAmp Fast Optical 96-well) in duplicate 20 μL reaction volume with 4 ng/μL cDNA/well on a QuantStudio3 qPCR machine. Thermal cycling consisted of a 2-minute 95°C hold, 40 cycles of 95°C for 5 seconds then 60°C for 25 seconds, and a melt curve determination stage. Comparative Ct was used to analyze a standard curve plot for each primer (dilutions 1:1, 1:10, 1:50, 1:100) with log cDNA concentration by corresponding Ct values. The average Ct value for every sample was plotted on the linear curve fit line and normalized to the geometric mean of housekeeping genes *CycloA* and *Tbp*.

### RNA-Seq and pattern analysis

Approximately 1 μg of RNA was submitted for RNA-Seq by BGI Tech Global, where mRNA was enriched by oligo dT, fragmented, and reverse-transcribed using random N6-primed RT to generate a cDNA library suitable for sequencing. The DNBSEQ platform was used to perform paired-end 100 bp sequence reads, generating an average of 4.56G Gb per sample. Sequencing data were filtered using SOAPnuke, and HISAT and Bowtie2 (69) were used to align clean reads to the *Mus musculus* reference genome version GCF_000001635.26_GRCm38.p6 with an average mapping ratio of 97.7% and 80.2%, respectively; 18,008 genes were identified.

RSEM was used to calculate the gene expression level of each sample (70). Rounded RSEM counts were loaded into DESeq2 for sequencing depth and RNA composition normalization (R/Bioconductor DESeq2; Bioconductor Open-Source Software for Bioinformatics; ref. 71). Low-expressed genes across all samples were filtered out (<1 read); 17,665 genes remained. Further filtering ensured at least 5 samples (sample size of the smallest experimental group) with a count of 10 or more; 14,364 genes remained for downstream differential expression and pattern analysis.

Differential expression analysis was performed by likelihood ratio test without independent filtering, comparing the full model (genotype + diabetes + genotype × diabetes) to a reduced model (genotype + diabetes) to isolate the interaction between *Sarm1* knockout and T1D. This generated a list of genes defined as *P* < 0.3 that was used for pattern analysis using DEGpattern in DEGreport. The subset of DEGs with log_2_ fold-change>|0.345| (>1.27-fold) was retained for pathway enrichment analysis. This data set was cross-checked by graphing the transcripts per million (TPM) values provided by BGI Tech Global.

Pathway enrichment analysis of DEGs was performed using ShinyGO version 0.77 (72) relative to a background list of the 18,008 genes identified by RNA-Seq. Analyses included the following pathway databases: Gene Ontology (GO) biological process, GO molecular function, Reactome, and Kyoto Encyclopedia of Genes and Genomes with FDR cutoff 0.05 and min-max pathway size (2 to 2,000). Results are reported as FDR, number of genes, and fold-enrichment of each pathway.

### Statistics

General biostatistical comparisons were performed in GraphPad Prism software. Changes over time between 2 groups were evaluated by 2-way ANOVA with Holm-Šídák multiple comparisons test (e.g., genotype × time). Changes over time between 4 groups were compared by 3-way ANOVA or mixed model with Tukey’s multiple comparisons test (e.g., genotype × diabetes × time). Contrasts between 3 groups at a single time point were evaluated using 1-way ANOVA with Holm-Šídák multiple comparisons test. A *P* value less than 0.05 was considered significant. For 2- and 3-way ANOVA, if no significant interaction term, significant individual effects of independent variables are presented; if the interaction is significant, this is presented in the figures.

### Study approval

All animal experiments were fully approved by the Animal Studies Committee at Washington University.

### Data availability

Values for all data points in graphs are reported in the Supporting Data Values file. Supporting R code for the pattern analysis is available by request. The full RNA-Seq data set is appended as read counts and TPM in the Supporting Data Values file and can also be explored on Gene Expression Omnibus under accession GSE252292.

## Author contributions

JMB, IRS, CSC, JM, AD, and ELS conceptualized the research aims and, with ML, AS, NKW, and GAM, developed the methodology. Investigation and validation were executed by JMB, IRS, CSC, KLM, JSP, ML, AS, NKW, XZ, ATB, and ELS. JMB, IRS, CSC, KLM, JSP, ML, AS, NKW, and ELS contributed to formal analysis and data curation. IRS implemented software code and supporting algorithms. JMB, IRS, and ELS prepared the original draft and data visualization and revised the manuscript with editing from NKW, GAM, and AD. GAM, JM, AD, and ELS provided resources and supervision, and ELS was responsible for project administration and funding acquisition.

## Supplementary Material

Supplemental data

Supporting data values

## Version 1 01/04/2024

In-Press Preview

## Version 2 02/22/2024

Electronic publication
