# Supplementary material for: Sarm1 knockout prevents type 1 diabetic bone disease in females independent of neuropathy
Source: JCI Insight. 2024 Jan 4;9(4):e175159. doi: 10.1172/jci.insight.175159 (PMC11143934; doi:10.1172/jci.insight.175159)
Supplement: Supplemental data [file jciinsight-9-175159-s114.pdf]

SUPPLEMENTAL METHODS

Generation of Sarm1^{2xFLAG} and *Sarm1-fl/fl* mouse models

The Sarm1^{2xFLAG} mouse was created using synthetic gRNA ordered from IDT with the following spacer sequence (PAM): 5’- acaggttaaggcagacccat(tgg), and ssODN 5’-c*a*ggactcctctgccggatcggataccagtttggagggagctacgccaatgggtctgcctggtggaggtggaagcGACTATAAGGATGACGACGACAAAGATTACAAGGACGACGATGATAAGtaacctgtccccagttcccgtgccctactgtgactcctgatttagttccctgcccttaa*a*g. For the *Sarm1-fl/fl* mouse, gRNAs were designed to mediate insertion of loxP sites in Introns 1 and 2, floxing the Exon 2 with 619 bp coding sequences. Synthetic gRNAs were ordered from Synthego, with intron 1 spacer sequence (PAM): 5’- agcatccatatcgagccaca(tgg), and intron 2, 5’-gtgaaacatacccaagggtg(ggg). A single-stranded olgio DNA donor (ssODN) carrying loxP in the CRISPR cleavage site flanked by short homology arms was used as a donor template with each gRNA. Sequences of ssODN for intron 1, 5’-g*a*ccaaaagagagcaaaggttccctggaactaaagtcacaggcagttgtgagccatgtATAACTTCGTATAATGTATGCTATACGAAGTTATGGATCCggctcgatatggatgctgggagctgagctctggacctcgttgccaagagcaacaagc*a*c, and for intron 2, 5’-g*a*agtgatctgtaactggaatttggagtacttgtaagaatagagtgaaacatacccaaggATAACTTCGTATAATGTATGCTATACGAAGTTATGGATCCgtggggccgtatgtctgcagaggcaggcagacctctgtgactttgaggttacctgg*t*c. All ssODNs were ordered as Ultramers from IDT. *stands for a phosphonothioate bond. The gRNA/Cas9 protein complexes were transfected along with the ssODNs into N2A cells and validated by NGS for efficient incorporation of cleavage and insertion of loxP before the reagents were used in mouse sessions.

For CRISPR electroporation, C57BL/6J mice at 3-4 weeks of age (JAX Laboratories, Bar Harbor ME, USA) were superovulated by intraperitoneal injection of 5 IU pregnant mare serum gonadotropin, followed 47h later by intraperitoneal injection of 5 IU human chorionic gonadotropin (PMS from SIGMA, HGC from Millipore USA). Mouse zygotes were obtained by mating C57BL/6J stud males with superovulated C57BL/6J females at a 1:1 ratio. One-cell fertilized embryos were electroporated with an RNP containing 12 µg of Cas9 protein, 2 µg of each gRNA, and 100 pmol (~ 5 µg) of each ssODN complex. Electroporations and mouse transgenesis experiments were performed as described previously (1–3). Live births were genotyped during validation using NGS.

Immunostaining, imaging, and analysis of foot pads

Briefly, OCT-embedded tissues were sectioned at 50 µm thickness into PBS as a four well series. One series of free-floating sections per mouse was stained in mesh nets as follows: wash 3x5 minutes in TNT buffer (0.1M Tris-HCl pH 7.4; 0.15M NaCl; 0.05% Tween-20), incubate with blocking solution for 1-hour at room temp (0.1M PBS with 0.05% Tween-20 and 5% normal donkey serum), incubate with primary antibody cocktail overnight at 4˚C (Anti-PGP9.5, Millipore AB1761-I, 1:1000; Anti-CGRP, Bio-Rad 1720-9007, 1:1000). The next day sections were washed in TNT 3x10 minutes prior to incubation with secondary antibody cocktail for 2.5 hours at room temperature (Jackson Immunoresearch, Donkey Anti-Rabbit AlexaFluor488 1:500 and Donkey Anti-Goat AlexaFluor594 1:500). Sections were then washed again in TNT 3x10 min, stained with DAPI (Sigma), washed once more 3x5 min and mounted in glycerol medium for imaging. Multiple sections were imaged per animal on a Nikon spinning disk confocal microscope (µm/px = 0.650, overlap = 10%) and analysis was performed using FIJI to trace and measure the epithelial basement membrane and to count the number of intraepidermal nerve fiber crossings (4).

Immunostaining of 2xFLAG mouse tissues

Mice were perfused with PBS followed by 10% neutral buffered formalin prior to tissue post-fixation for 24-hours, dehydration, and paraffin processing. Paraffin embedded tissues were sectioned at 10 µm thickness and stained as follows. Briefly, tissue samples were permeabilized for 10-minutes and blocked for 1.5-hours with the buffer provided from Vectastain Elite ABC kit (Vector Laboratories). Primary antibody cocktail (1:500 Anti-FLAG M2 antibody, Cell Signaling #14793) was made in TNT buffer containing 2% donkey serum and incubated overnight at 4 °C. Tissue sections were washed in TNT followed by quenching of endogenous peroxidases with 0.3% hydrogen peroxide in PBS for 30-minutes. Sections were washed 3x again in TNT prior to incubation with kit-provided secondary antibody (ImPRESS) for 30-minutes. After the secondary antibody, tissue samples were again washed 3x in TNT and 2x in PBS. Tissues were incubated with DAB substrate solution stain development, then washed in water. After a final wash, samples were stained with hematoxylin, dehydrated through a reverse ethanol gradient and mounted with Permount mounting media.

Embedding and cryosectioning of non-decalcified bone

Following euthanasia, tibiae with intact periosteum were fixed in 10% NBF overnight, washed 3x30 minutes in PBS and, cryopreserved in fresh 30% sucrose at 4˚C for 16-hours. Tibiae were then moved to a new tube overnight in OCT mounting media to promote infiltration (Fisher Health Care 23–730-571) prior to placing in embedding in plastic cryomolds on a cold plate. Blocks were stored at -80˚C. During sectioning, tissues were adhered to adhesive tape windows (Leica 39475214) and cut at 5 µm on a cryostat (Leica) with low-profile gold-coated blades (C.L. Sturkey DT315G50). Sectioned tissues were transferred onto TruBond 380 adhesive slides (Electron Microscopy Sciences 63700B10) and secured by Norland Adhesive 63. A desktop UV transilluminator was used to cure the adhesive for 1-minute. Slides were imaged with a 10x objective on a Nikon spinning disk confocal microscope (µm/px = 0.650, overlap = 10%) or NanoZoomer (10x, Hamamatsu Photonics). Images were analyzed using BioQuant 2021 software with the program “Bone Cortical Basic.” Endocortical dynamic histomorphometry data were obtained using EcPm - Endocrotical Perimeter, sLS – Single Labeled Surface, and dLS – Double Labeled Surface functions.

1. Sentmanat MF, White JM, Kouranova E, Cui X. Highly reliable creation of floxed alleles by electroporating single-cell embryos. *BMC Biol.* 2022;20(1):31.

2. Pease S, Saunders TL (eds). *Advanced protocols for animal transgenesis*. Berlin, Heidelberg: Springer Berlin Heidelberg; 2011:

3. Manipulating the Mouse Embryo: A Laboratory Manual, Fourth Edition [Internet]https://www.cshlpress.com/default.tpl?cart=168994922732483571&fromlink=T&linkaction=full&linksortby=oop_title&--eqSKUdatarq=982. cited July 21, 2023

4. Ebenezer GJ et al. Denervation of skin in neuropathies: the sequence of axonal and Schwann cell changes in skin biopsies.. *Brain* 2007;130(Pt 10):2703–2714.

SUPPLEMENTAL TABLE 1

	Parameter	Control (n=11)	T1D – Mod (n=9)	T1D – Severe (n=10)	1-way ANOVA
Metabolism and Body Composition	Fed glucose (mg/dL)	155±19	289±63[a]	544±61[a,b]	p<0.001*
	Fasting glucose (mg/dL)	147±31	176±61[a]	476±68[a,b]	p<0.001*
	Serum insulin (pg/mL)	440±137	243±96[a]	122±27[a]	p<0.001*
	Body mass (g)	27.1±1.5	24.7±1.2[a]	22.3±2.4[a,b]	p<0.001*
	Lean mass (g)	18.9±1.0	17.0±1.2[a]	16.6±1.5[a,b]	p<0.001*
	Lean mass (%)	78.6±3.9	76.2±3.7	82.2±1.7[b]	p=0.002*
	Fat mass (g)	5.2±1.1	5.3±0.8	3.6±0.8[a,b]	p<0.001*
	Fat mass (%)	21.4±3.9	23.8±3.7	17.8±2.7[b]	p=0.002*
Bone Morphology (Tibia)	Length (mm)	17.8±0.2	17.6±0.4	17.4±0.4[a]	p=0.026*
	Ct.Total Area (mm^2)	1.22±0.94	1.14±0.09	1.11±0.08[a]	p=0.019*
	Ct.Bone Area (mm^2)	0.67±0.05	0.61±0.04[a]	0.52±0.05[a,b]	P<0.001*
	Ct.Marrow Area (mm^2)	0.56±0.06	0.53±0.06	0.59±0.07	p=0.061
	Ct.Thickness (mm)	0.198±0.008	0.190±0.006	0.157±0.018[a,b]	p<0.001*
	Ct.BMC (mgHA/ccm)	1097±15	1093±8	1078±14[a,b]	p=0.006*
	Tb.TV (mm3)	1.85±0.13	1.72±0.24	1.85±0.12	p=0.162
	Tb.BV (mm3)	0.363±0.072	0.323±0.080	0.297±0.036	p=0.092
	Tb.BVF (%)	19.7±4.4	19.1±5.2	16.1±2.2	p=0.213
	Tb.Thickness (mm)	0.064±0.004	0.060±0.004	0.054±0.003[a,b]	p<0.001*
	Tb.Number (1/mm)	4.89±0.53	5.12±0.68	5.22±0.40	p=0.400
	Tb.BMD (mgHA)	216±26	212±31	192±14	p=0.079
Bone Morphology and Strength (Femur)	Ct.Total Area (mm^2)	1.90±0.14	1.82±0.15	1.72±0.11[a]	p=0.028*
	Ct.Bone Area (mm^2)	0.94±0.07	0.88±0.11	0.74±0.10[a,b]	p<0.001*
	Ct.Marrow Area (mm^2)	0.96±0.11	0.94±0.12	0.99±0.10	p=0.566
	Ct.Thickness (mm)	0.240±0.023	0.231±0.042	0.188±0.032[a,b]	p=0.003*
	Stiffness (N/mm)	95±13	98±18	76±21[a,b]	p=0.018*
	Yield Load (N)	9.9±3.2	8.7±2.3	6.4±2.4[a]	p=0.021*
	Max Load (N)	17.8±1.5	16.3±2.7	13.6±3.3[a]	p=0.002*
	Post-Yield Displacement (mm)	0.457±0.108	0.391±0.115	0.495±0.368	p=0.625
	Work to Fx (N*mm)	7.2±1.6	5.6±1.9	5.1±3.3	p=0.146
	pMOI	0.471±0.072	0.421±0.083	0.343±0.064[a]	p=0.002*
	Ultimate Stress (N/mm^2)	142±18	137±16	131±25	p=0.467
Nerve density, function, and behavior	Rotarod (avg, ms)	49.0±12.0	48.1±12.7	42.5±6.0	p=0.343
	Inverted screen (avg, s)	118±8	120±0	106±31	p=0.221
	Hot plate first rxn (˚C)	38.5±1.9	38.8±1.8	41.2±2.1[a]	p=0.008*
	Sciatic amplitude (mV)	2.62±0.70	2.59±0.86	1.73±0.66[a,b]	p=0.019*
	Sciatic latency (ms)	1.86±0.17	1.97±0.20	2.12±0.22[a]	p=0.021*
	Sciatic NCV (m/s)	35.8±5.3	36.0±5.0	33.7±4.39	p=0.508
	Sural amplitude (mV)	3.17±0.83	2.90±1.00	2.12±0.78[a]	p=0.029*
	Sural latency (ms)	1.18±0.10	1.30±0.16	1.40±0.18[a]	p=0.008*
	CGRP+ axon density (#/mm epithelium)	10.7±5.9	11.7±5.9	8.77±5.9	p=0.546
	PGP9.5+ axon density (#/mm epithelium)	26.0±7.5	30.9±8.0	26.6±10.8	p=0.428

Supplemental Table 1. T1D severity outcomes. 1-way ANOVA (*p<0.05) with Tukey's multiple comparisons test ([a]p<0.05 vs control; [b]p<0.05 vs T1D-mod). Ct=cortical. BMC=bone mineral content. Tb=trabecular. BVF=bone volume fraction. BMD=bone mineral density. pMOI=polar moment of inertia. GRP=calcitonin gene related peptide. PGP9.5=protein gene product 9.5. Mean ± Standard Deviation.

SUPPLEMENTAL TABLE 2

	Parameter	**Sarm1$^{WT/WT}$ male** (n=10)	**Sarm1$^{KO/KO}$ male** (n=10)	**Sarm1$^{WT/WT}$ female** (n=14)	**Sarm1$^{KO/KO}$ female** (n=14)
Metabolism and Body Composition	Fasting glucose (mg/dL)	175±26	168±36	139±32	130±23
	Body mass (g)	20.3±1.9	20.9±1.4	17.8±1.0	17.4±1.6
	Lean mass (g)	15.9±1.4	16.7±1.1	14.3±0.8	13.7±1.0
	Lean mass (%)	82±1.9	83±2.0	80±2.9	82±1.2
	Fat mass (g)	2.6±0.5	2.5±0.3	2.5±0.5	2.1±0.2 [a]
	Fat mass (%)	13±1.7	12±1.4	14±2.4	13±0.92
Bone Morphology	Ct.Total Area (mm^2)	0.95±0.08	0.99±0.07	0.93±0.07	0.89±0.07
	Ct.Marrow Area (mm^2)	0.49±0.02	0.48±0.04	0.49±0.03	0.48±0.04
	Ct.Thickness (mm)	0.15±0.02	0.17±0.02	0.15±0.009	0.14±0.005
	Ct.BMC (mgHA/ccm)	1006±29	995±29	976±34	974±28
	Tb.BVF (%)	11.5±3.5	13.4±2.9	9.1±1.3	10±2.0
	Tb.Thickness (mm)	0.049±0.005	0.049±0.003	0.048±0.003	0.047±0.002
	Tb.Number	3.9±0.6	4.6±0.7 [a]	3.1±0.5	3.5±0.5 [a]
	Tb.BMD (mgHA)	141±21	159±20	122±7	131±12 [a]
Nerve function	Sciatic amplitude (mV)	3.9±0.7	3.9±1.1	3.6±0.9	4.1±0.7
	Sciatic latency (ms)	2.2±0.4	2.2±0.2	2.3±0.3	2.3±0.3
	Sciatic NCV (m/s)	26±8.8	29±5.2	24±4.5	28±7.0
	Sural amplitude (mV)	6.7±1.1	5.6±1.3	6.4±1.3	7.0±1.1
	Sural latency (ms)	1.3±0.1	1.4±0.2	1.4±0.1	1.4±0.2

Supplemental Table 2. Control wild type (WT) vs Sarm1^{KO} basal phenotyping. Unpaired t-test in male and female mice at 8-weeks of age. [a]$p<0.05$ vs WT. Ct=cortical. BMC=bone mineral content. Tb=trabecular. BVF=bone volume fraction. BMD=bone mineral density. Mean ± Standard Deviation.

SUPPLEMENTAL FIGURE 1

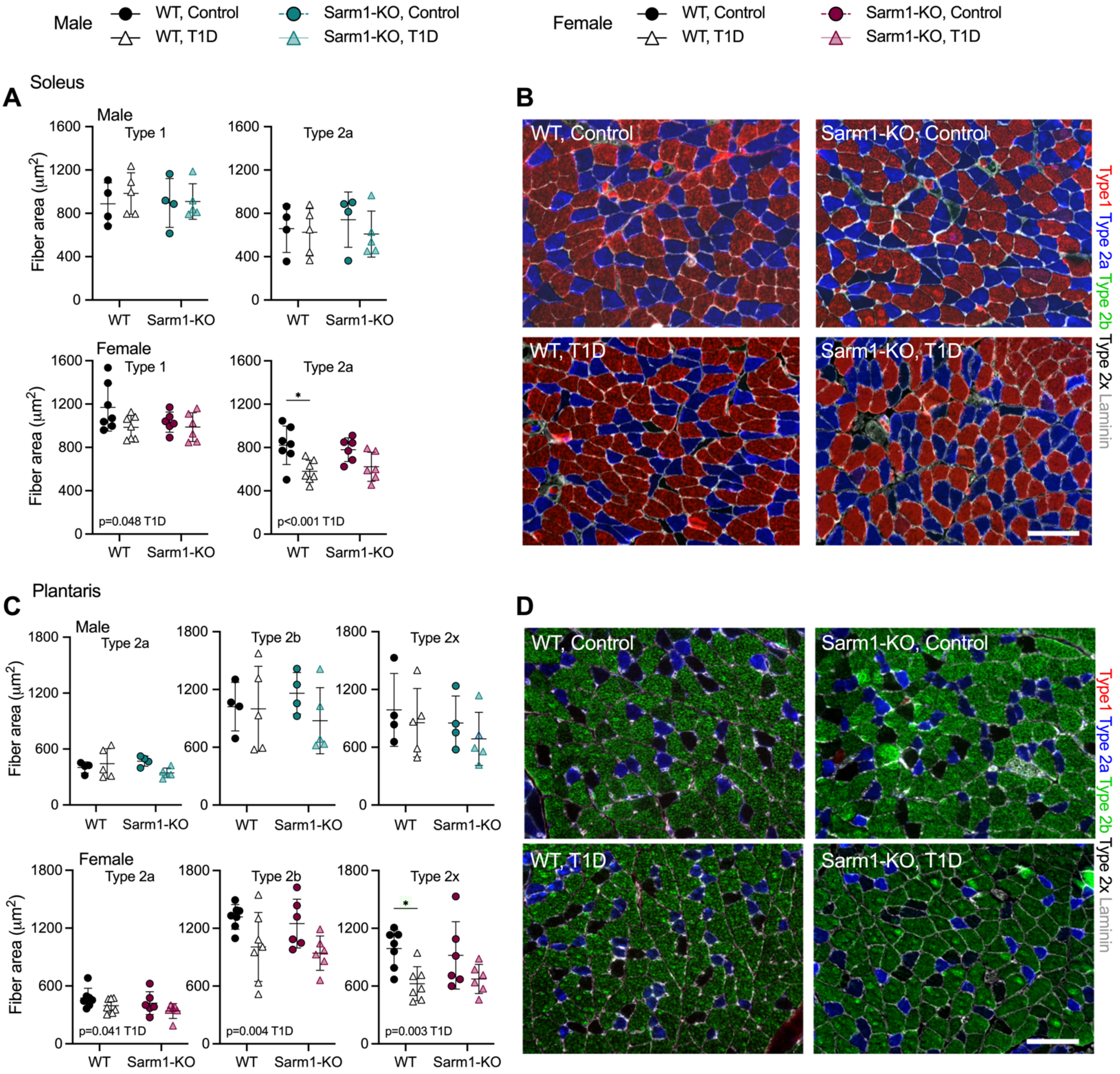

Supplemental Figure 1. STZ-induced T1D impairs muscle architecture of control and Sarm1^{KO} mice. Control and Sarm1^{KO} mice at 8-weeks of age were treated with STZ to induce T1D, controls received 0.9% saline. Muscle fiber cross-sectional area was evaluated at 15-weeks after induction of T1D. **(A and B)** Soleus muscle. (**C** and **D**) Plantaris muscle. Representative 20x images of type 1 (red), type 2a (blue), and type 2b (green) muscle fibers by immunostaining for myosin heavy chain isoforms with unstained fibers designated as type 2x (black). Laminin immunostaining (gray) labeled the fiber perimeter and facilitated quantification. (**A** and **C**) 2-way ANOVA for genotype x T1D. Graphed as Mean ± SD. n=4-7/group.

SUPPLEMENTAL FIGURE 2

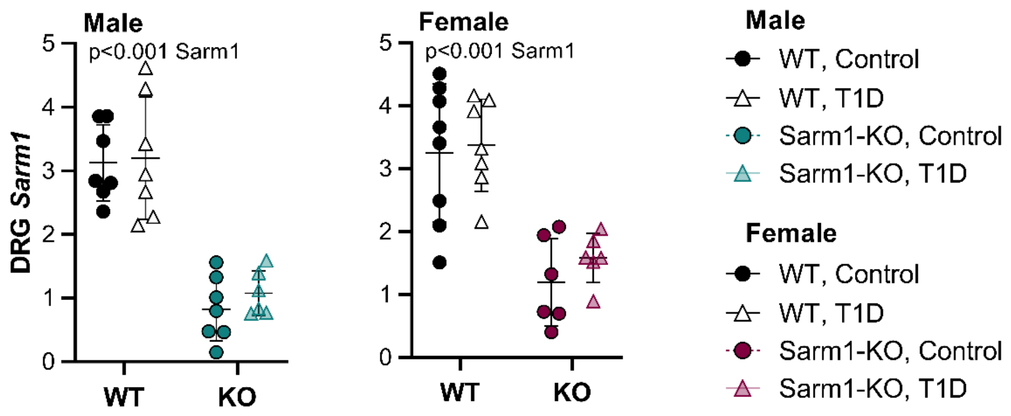

Supplemental Figure 2. *Sarm1* gene expression. Relative *Sarm1* gene expression by qPCR in the dorsal root ganglia (DRG) 3-weeks following the induction of T1D. Data points represent individual mice. Graphed as mean ± SD. 2-way ANOVA for genotype x T1D.

SUPPLEMENTAL FIGURE 3

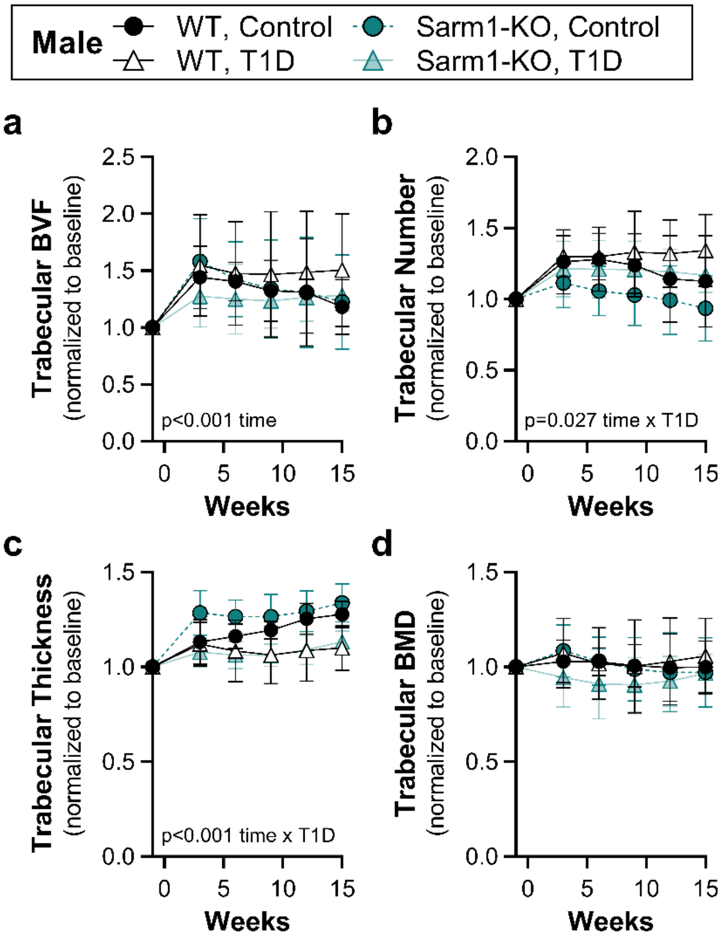

Supplemental Figure 3. Longitudinal analysis of male trabecular bone in WT and Sarm1^{KO} mice with T1D. WT control and Sarm1^{KO} mice at 8-weeks of age were treated with STZ to induce T1D, controls received 0.9% saline. Longitudinal in vivo microCT scans of the tibia were taken at the proximal metaphysis. Tibia trabecular bone volume fraction (BVF) **(a)**, trabecular number **(b)**, trabecular thickness **(c)**, and trabecular bone mineral density (BMD) **(d)**. 3-way ANOVA/mixed model for genotype x T1D x time. Each individual normalized to baseline. All data plotted as Mean ± Standard Deviation. n=4-5/group.

SUPPLEMENTAL FIGURE 4

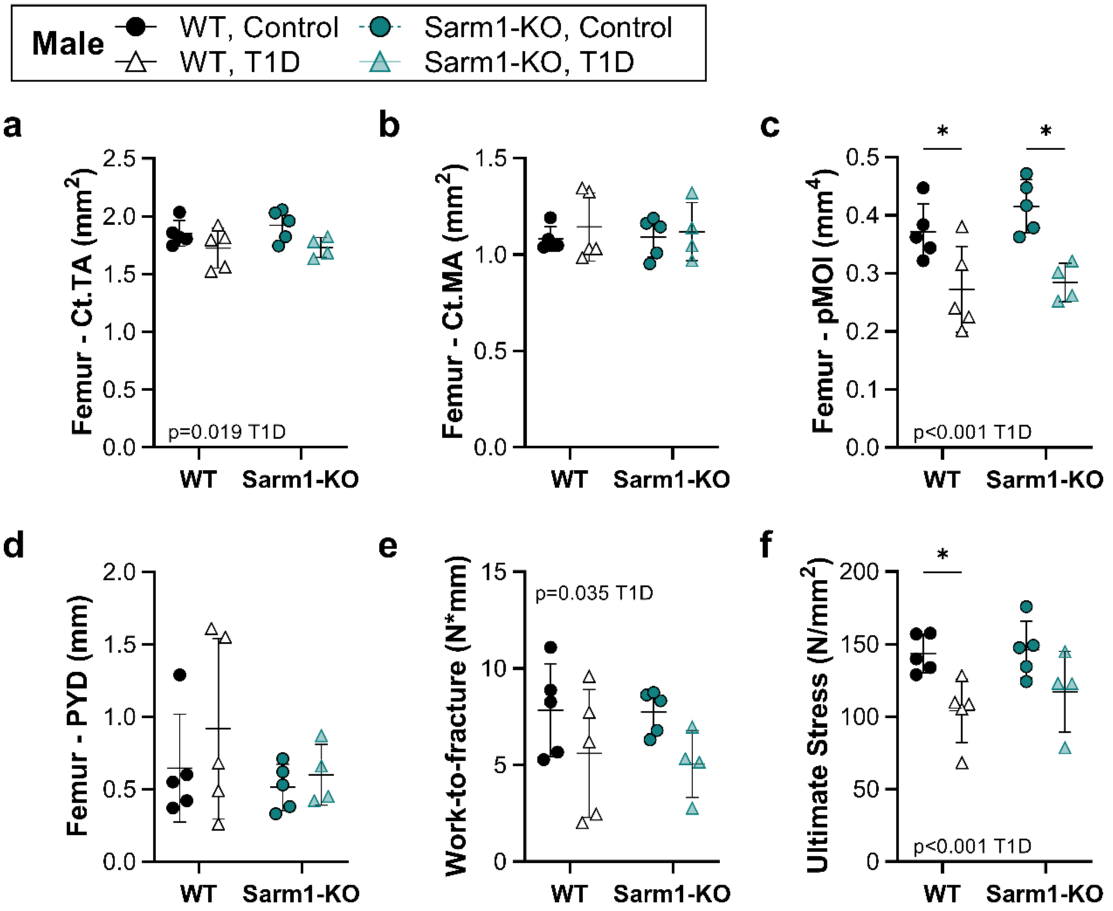

Supplemental Figure 4. MicroCT and three-point bending biomechanics of male femur in WT and Sarm1^{KO} mice with T1D. WT control and Sarm1^{KO} mice at 8-weeks of age were treated with STZ to induce T1D, controls received 0.9% saline. At the 15-week end point femur cortical properties at the 50% site were analyzed by *ex vivo* microCT prior to three-point bend biomechanical testing. **(a)** Femur cortical total area. **(b)** Femur cortical marrow area. **(c)** Femur polar moment of inertia. **(d)** Femur post-yield displacement. **(e)** Femur work to fracture. **(f)** Femur ultimate stress. 2-way ANOVA for genotype x T1D with Tukey's multiple comparisons test. All data plotted as Mean ± Standard Deviation. n=4-5/group.

SUPPLEMENTAL FIGURE 5

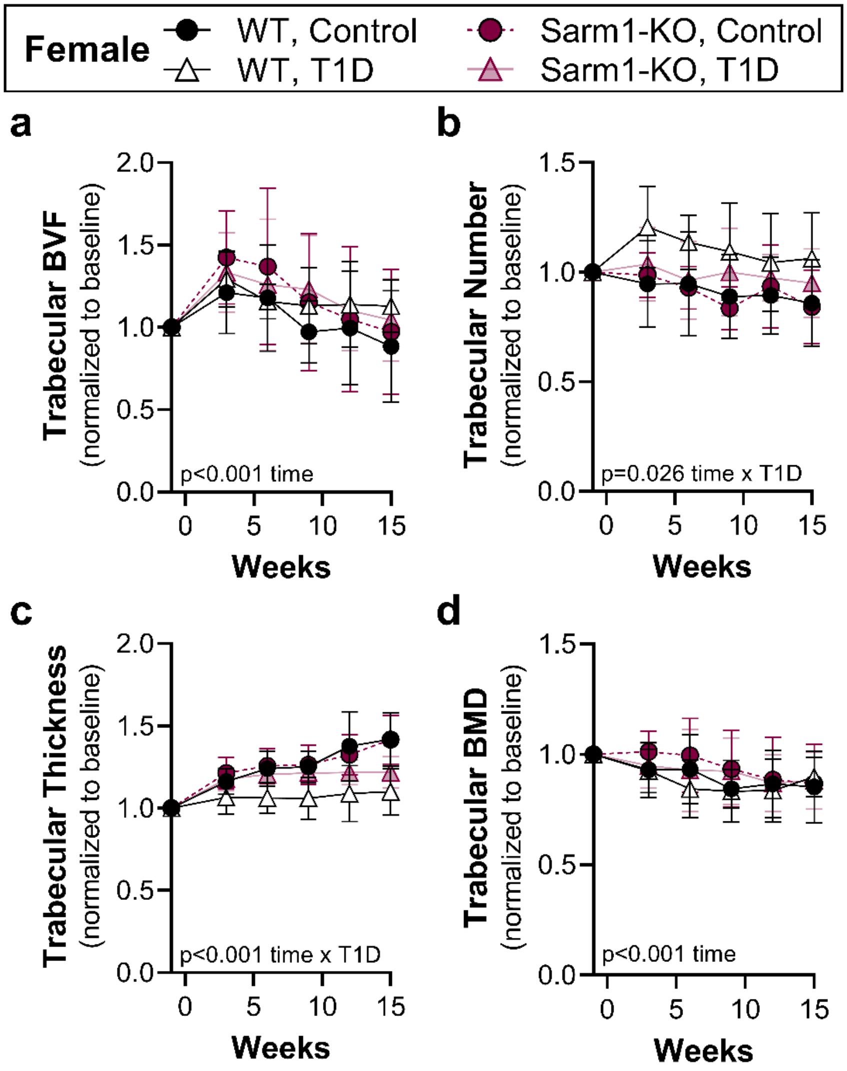

Supplemental Figure 5. Longitudinal analysis of female trabecular bone in WT and Sarm1^{KO} mice with T1D. WT control and Sarm1^{KO} mice at 8-weeks of age were treated with STZ to induce T1D, controls received 0.9% saline. Longitudinal in vivo microCT scans of the tibia were taken at the proximal metaphysis. Tibia trabecular bone volume fraction (BVF) **(a)**, trabecular number **(b)**, trabecular thickness **(c)**, and trabecular bone mineral density (BMD) **(d)**. 3-way ANOVA/mixed model for genotype x T1D x time. Each individual normalized to baseline. All data plotted as Mean ± Standard Deviation. n=6-7/group.

SUPPLEMENTAL FIGURE 6

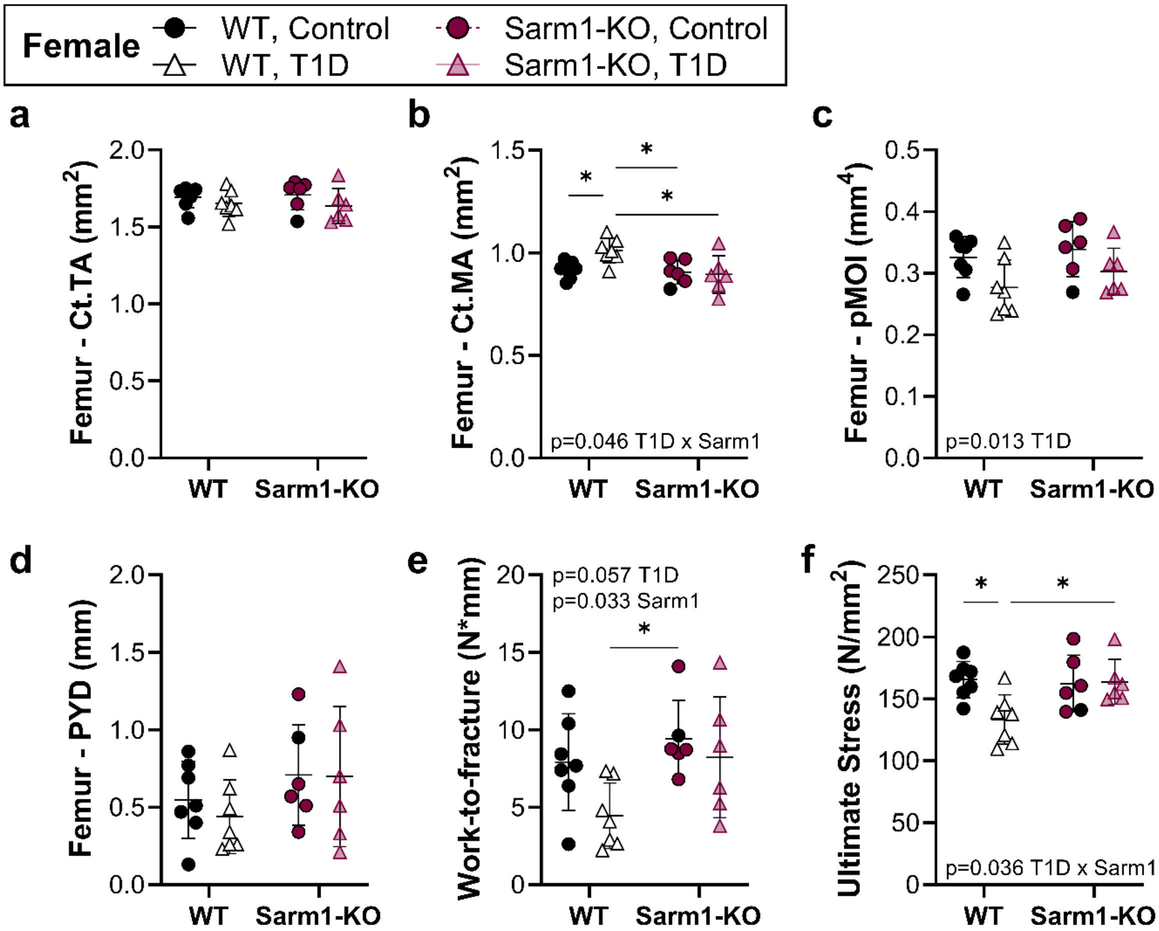

Supplemental Figure 6. MicroCT and three-point bending biomechanics of female femur in WT and Sarm1^{KO} mice with T1D. WT control and Sarm1^{KO} mice at 8-weeks of age were treated with STZ to induce T1D, controls received 0.9% saline. At the 15-week end point femur cortical properties at the 50% site were analyzed by *ex vivo* microCT prior to three-point bend biomechanical testing. **(a)** Femur cortical total area. **(b)** Femur cortical marrow area. **(c)** Femur polar moment of inertia. **(d)** Femur post-yield displacement. **(e)** Femur work to fracture. **(f)** Femur ultimate stress. 2-way ANOVA for genotype x T1D with Tukey's multiple comparisons test. All data plotted as Mean ± Standard Deviation. n=6-7/group.

SUPPLEMENTAL FIGURE 7

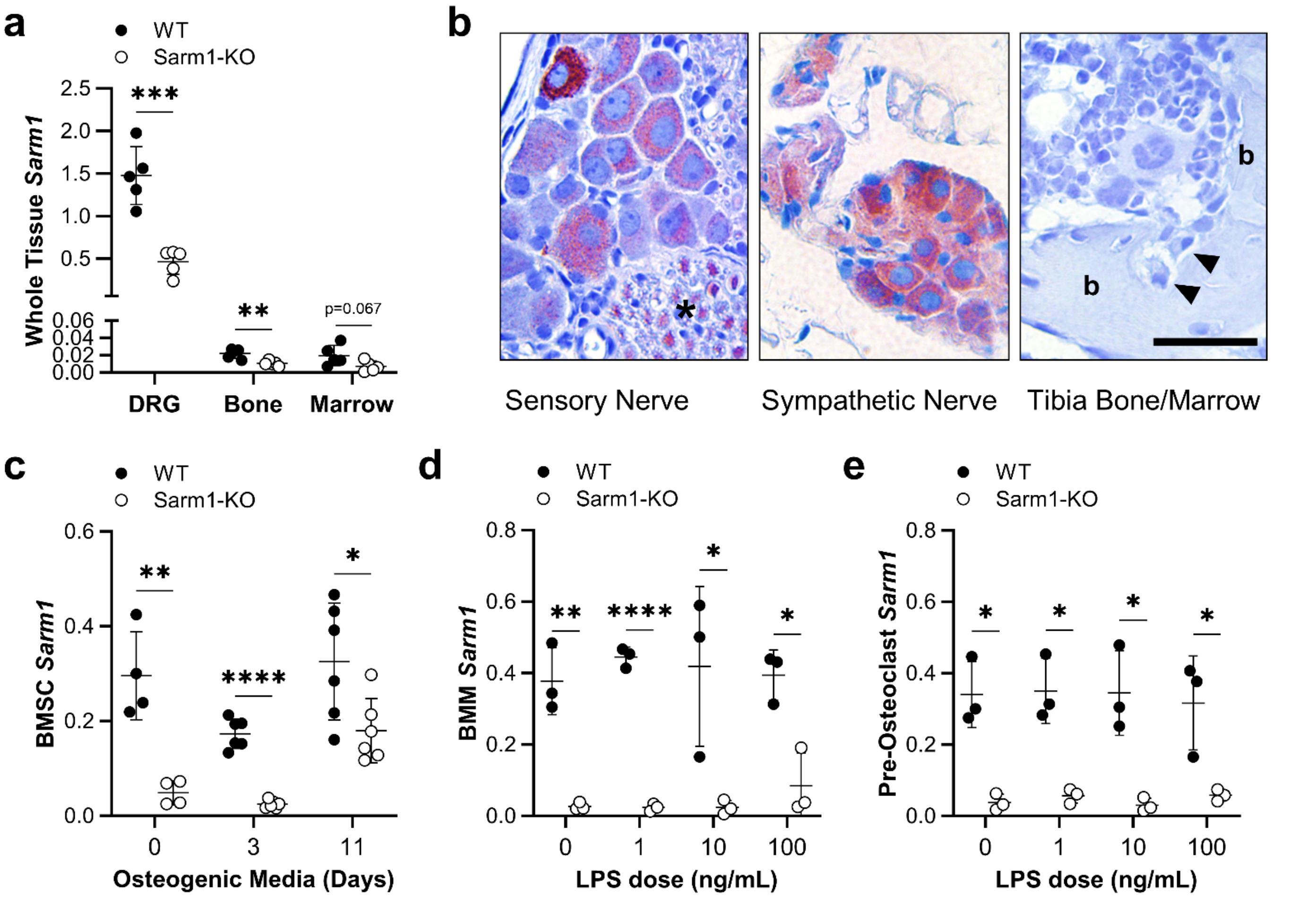

Supplemental Figure 7. *Sarm1* gene expression. (a) Relative *Sarm1* gene expression by qPCR in the neuroskeletal system including the dorsal root ganglia (DRG), bone (marrow removed), and bone marrow at baseline (8-week-old, mixed males and females). **(b)** Anti-FLAG immunohistochemistry from transgenic mice expressing FLAG-tagged SARM1 in DRG (sensory nerve cell bodies and axons*), aorticorenal ganglia (sympathetic nerve cell bodies), and tibia bone (b=bone). Arrowheads = osteoblasts. Scale = 50 µm. Brown = positive stain. **(c,d,e)** Relative *Sarm1* gene expression by qPCR in cultured cells. Data points represent individual wells of a representative study. **(c)** Bone marrow stromal cells (BMSCs) differentiated to osteoblasts for 0, 3, or 11 days. **(d)** Bone marrow macrophages (BMMs) treated for 4-hours with the indicated doses of LPS. **(e)** Pre-osteoclasts (BMMs differentiated for 3-days in osteoclastogenic media) treated for 4-hours with the indicated doses of LPS. Graphs presented as mean ± SD. *p<0.05, **p<0.01, ***p<0.001, ****p<0.0001

SUPPLEMENTAL TABLE 3

Supplemental Table 3. Pattern analysis results. List of 'rescued' genes with >|0.35| Log_2fold change in WT T1D that remain unchanged with Sarm1^{KO} by statistical pattern analysis.

Gene ID	Gene Name	Description	Log2FC (WT T1D/Con)	pvalue Gene x T1D	Cluster
12824	*Col2a1*	collagen, type II, alpha 1	-1.19	1.82E-02	1
212712	*Satb2*	special AT-rich sequence binding protein 2	-1.14	2.27E-02	1
12842	*Col1a1*	collagen, type I, alpha 1	-1.14	1.25E-02	1
18675	*Phex*	phosphate regulating endopeptidase homolog, X-linked	-1.05	2.78E-02	1
12843	*Col1a2*	collagen, type I, alpha 2	-1.01	2.38E-02	1
18187	*Nrp2*	neuropilin 2	-0.94	1.23E-03	1
268396	*Sh3pxd2b*	SH3 and PX domains 2B	-0.93	3.02E-03	1
12817	*Col13a1*	collagen, type XIII, alpha 1	-0.90	3.79E-02	3
19228	*Pth1r*	parathyroid hormone 1 receptor	-0.87	2.48E-02	3
12831	*Col5a1*	collagen, type V, alpha 1	-0.86	2.89E-02	1
93706	*Pcdhgc3*	protocadherin gamma subfamily C, 3	-0.81	9.28E-03	1
14086	*Fscn1*	fascin actin-bundling protein 1	-0.78	1.48E-02	1
15891	*Ibsp*	integrin binding sialoprotein	-0.76	1.53E-04	3
17534	*Mrc2*	mannose receptor, C type 2	-0.75	8.29E-03	1
12393	*Runx2*	runt related transcription factor 2	-0.75	7.37E-04	1
170643	*Kirrel*	kirre like nephrin family adhesion molecule 1	-0.74	1.01E-03	1
15530	*Hspg2*	perlecan (heparan sulfate proteoglycan 2)	-0.72	1.52E-02	1
110075	*Bmp3*	bone morphogenetic protein 3	-0.70	1.40E-02	1
14107	*Fat1*	FAT atypical cadherin 1	-0.68	2.10E-03	1
14182	*Fgfr1*	fibroblast growth factor receptor 1	-0.65	1.68E-02	1
235505	*Cd109*	CD109 antigen	-0.65	5.90E-03	3
69608	*Sec24d*	Sec24 related gene family, member D (S. cerevisiae)	-0.64	4.13E-02	1
72333	*Palld*	palladin, cytoskeletal associated protein	-0.64	2.40E-02	1
16779	*Lamb2*	laminin, beta 2	-0.64	2.95E-02	1
18830	*Pltp*	phospholipid transfer protein	-0.63	3.47E-03	1
18763	*Pkd1*	polycystin 1, transient receptor potential channel interacting	-0.61	4.23E-03	1
14183	*Fgfr2*	fibroblast growth factor receptor 2	-0.61	1.87E-05	1
16600	*Klf4*	Kruppel-like factor 4 (gut)	-0.61	2.87E-03	1
16880	*Lifr*	LIF receptor alpha	-0.60	1.50E-04	3
192187	*Stab1*	stabilin 1	-0.60	2.87E-03	3
14360	*Fyn*	Fyn proto-oncogene	-0.60	3.54E-02	1
12575	*Cdkn1a*	cyclin-dependent kinase inhibitor 1A (P21)	-0.59	1.38E-02	1
17387	*Mmp14*	matrix metallopeptidase 14 (membrane-inserted)	-0.59	1.06E-02	1
102693	*Phldb1*	pleckstrin homology like domain, family B, member 1	-0.58	1.80E-02	1
19223	*Ptgis*	prostaglandin I2 (prostacyclin) synthase	-0.57	1.89E-02	1
17470	*Cd200*	CD200 molecule	-0.57	2.31E-02	1
16973	*Lrp5*	low density lipoprotein receptor-related protein 5	-0.57	4.49E-03	1
215690	*Nav1*	neuron navigator 1	-0.57	2.49E-02	3
14961	*H2-Ab1*	histocompatibility 2, class II antigen A, beta 1	-0.57	3.76E-02	3
23917	*Impdh1*	inosine monophosphate dehydrogenase 1	-0.56	1.22E-03	1
212943	*Tent5a*	terminal nucleotidyltransferase 5A	-0.55	1.76E-02	1
69162	*Sec31a*	Sec31 homolog A (S. cerevisiae)	-0.55	9.89E-03	1
73137	*Prrc1*	proline-rich coiled-coil 1	-0.55	1.39E-02	1
70097	*Sash1*	SAM and SH3 domain containing 1	-0.54	3.96E-02	3
17909	*Myo10*	myosin X	-0.54	2.84E-02	3
69538	*Antxr1*	anthrax toxin receptor 1	-0.53	3.20E-02	3
74761	*Mxra8*	matrix-remodelling associated 8	-0.53	3.11E-02	3
69551	*2310022B05Rik*	RIKEN cDNA 2310022B05 gene	-0.53	3.17E-03	1
107338	*Gbf1*	golgi-specific brefeldin A-resistance factor 1	-0.52	9.33E-04	1
21413	*Tcf4*	transcription factor 4	-0.52	3.18E-03	3
13132	*Dab2*	disabled 2, mitogen-responsive phosphoprotein	-0.52	3.77E-02	3
13822	*Epb41l2*	erythrocyte membrane protein band 4.1 like 2	-0.51	4.17E-02	3
70361	*Lman1*	lectin, mannose-binding, 1	-0.51	1.88E-02	1
72007	*Fndc3b*	fibronectin type III domain containing 3B	-0.50	3.04E-02	1
223693	*Tmem184b*	transmembrane protein 184b	-0.49	2.05E-02	1
81910	*Rrbp1*	ribosome binding protein 1	-0.49	1.51E-02	3
227648	*Sec16a*	SEC16 homolog A, endoplasmic reticulum export factor	-0.48	4.21E-03	1
214968	*Sema6d*	sema domain, transmembrane domain (TM), and cytoplasmic domain, (semaphorin) 6D	-0.46	2.11E-02	3
224697	*Adamts10*	a disintegrin-like and metallopeptidase (reprolysin type) with thrombospondin type 1 motif, 10	-0.46	2.65E-02	3
268567	*Tmem229b*	transmembrane protein 229B	-0.46	3.04E-02	5

68137	*Kdelr1*	KDEL (Lys-Asp-Glu-Leu) endoplasmic reticulum protein retention receptor 1	-0.46	1.25E-02	1
68427	*Slc39a13*	solute carrier family 39 (metal ion transporter), member 13	-0.44	1.59E-02	3
20846	*Stat1*	signal transducer and activator of transcription 1	-0.44	3.88E-02	3
16971	*Lrp1*	low density lipoprotein receptor-related protein 1	-0.44	1.35E-02	1
228357	*Lrp4*	low density lipoprotein receptor-related protein 4	-0.42	1.22E-02	3
11933	*Atp1b3*	ATPase, Na+/K+ transporting, beta 3 polypeptide	-0.41	2.05E-02	1
14270	*Srgap2*	SLIT-ROBO Rho GTPase activating protein 2	-0.41	1.82E-02	3
105722	*Ano6*	anoctamin 6	-0.40	2.61E-02	1
194401	*Mical3*	microtubule associated monooxygenase, calponin and LIM domain containing 3	-0.40	4.15E-02	1
69719	*Cad*	carbamoyl-phosphate synthetase 2, aspartate transcarbamylase, and dihydroorotase	-0.40	2.31E-03	1
52398	*Septin11*	septin 11	-0.40	3.29E-02	1
14886	*Gtf2i*	general transcription factor II I	-0.39	7.11E-03	1
98952	*Fam102a*	family with sequence similarity 102, member A	-0.39	2.63E-02	1
192657	*Ell2*	elongation factor for RNA polymerase II 2	-0.39	4.08E-02	5
14104	*Fasn*	fatty acid synthase	-0.39	1.61E-02	1
14972	*H2-K1*	histocompatibility 2, K1, K region	-0.39	1.71E-02	3
21923	*Tnc*	tenascin C	-0.39	3.64E-02	3
70661	*Sik3*	SIK family kinase 3	-0.38	7.83E-03	1
219140	*Spata13*	spermatogenesis associated 13	-0.37	1.53E-03	5
100952	*Emilin1*	elastin microfibril interfacer 1	-0.37	3.02E-02	1
18822	*Plod1*	procollagen-lysine, 2-oxoglutarate 5-dioxygenase 1	-0.36	3.28E-02	1
338366	*Mia3*	MIA SH3 domain ER export factor 3	-0.36	1.37E-02	1
67972	*Atp2b1*	ATPase, Ca++ transporting, plasma membrane 1	-0.36	8.92E-03	3
68796	*Tmem214*	transmembrane protein 214	-0.35	2.24E-02	1
67074	*Mon2*	MON2 homolog, regulator of endosome to Golgi trafficking	-0.35	4.43E-03	1
20602	*Ncor2*	nuclear receptor co-repressor 2	-0.35	9.79E-03	1
19079	*Prkab1*	protein kinase, AMP-activated, beta 1 non-catalytic subunit	0.35	2.03E-02	2
72119	*Tpx2*	TPX2, microtubule-associated	0.35	8.88E-03	2
100383	*Bsdc1*	BSD domain containing 1	0.35	2.79E-02	2
17196	*Mbp*	myelin basic protein	0.36	2.20E-02	2
19941	*Rpl26*	ribosomal protein L26	0.36	3.77E-02	2
22241	*Ulk1*	unc-51 like kinase 1	0.36	3.98E-03	2
399510	*Map4k5*	mitogen-activated protein kinase kinase kinase kinase 5	0.36	1.43E-03	2
217869	*Eif5*	eukaryotic translation initiation factor 5	0.36	2.53E-02	2
12359	*Cat*	catalase	0.36	8.81E-04	2
71779	*Marchf8*	membrane associated ring-CH-type finger 8	0.36	2.32E-02	2
20115	*Rps7*	ribosomal protein S7	0.36	1.12E-02	2
12442	*Ccnb2*	cyclin B2	0.36	4.01E-02	2
27370	*Rps26*	ribosomal protein S26	0.36	1.93E-02	2
65246	*Xpo7*	exportin 7	0.36	3.35E-02	2
625249	*Gpx4*	glutathione peroxidase 4	0.36	2.96E-03	2
66949	*Trim59*	tripartite motif-containing 59	0.37	2.53E-02	2
12615	*Cenpa*	centromere protein A	0.37	1.38E-02	2
12215	*Bsg*	basigin	0.37	2.03E-03	2
74106	*Dcaf6*	DDB1 and CUL4 associated factor 6	0.37	9.48E-03	2
72401	*Slc43a1*	solute carrier family 43, member 1	0.37	3.87E-02	7
20491	*Sla*	src-like adaptor	0.37	1.67E-02	2
12785	*Cnbp*	cellular nucleic acid binding protein	0.37	1.83E-02	2
74737	*Pcf11*	PCF11 cleavage and polyadenylation factor subunit	0.37	1.21E-02	2
234797	*6430548M08Rik*	RIKEN cDNA 6430548M08 gene	0.37	4.06E-02	2
215387	*Ncaph*	non-SMC condensin I complex, subunit H	0.37	1.71E-02	2
230484	*Usp1*	ubiquitin specific peptidase 1	0.38	2.29E-03	2
214290	*Tut7*	terminal uridylyl transferase 7	0.38	1.84E-02	2
268449	*Rpl23a*	ribosomal protein L23A	0.38	3.67E-02	2
50849	*Rnf10*	ring finger protein 10	0.38	1.01E-02	2
218914	*Wapl*	WAPL cohesin release factor	0.38	2.05E-02	2
16551	*Kif11*	kinesin family member 11	0.38	9.45E-03	2
319171	*H2ac24*	H2A clustered histone 24	0.38	3.66E-02	2
107371	*Exoc6*	exocyst complex component 6	0.38	2.37E-02	2
12725	*Clcn3*	chloride channel, voltage-sensitive 3	0.38	4.01E-02	6
22329	*Vcam1*	vascular cell adhesion molecule 1	0.38	7.47E-03	2
13857	*Epor*	erythropoietin receptor	0.38	2.82E-02	2
94242	*Tinagl1*	tubulointerstitial nephritis antigen-like 1	0.38	3.29E-02	2
237890	*Slfn14*	schlafen 14	0.39	3.16E-02	2
67134	*Nop56*	NOP56 ribonucleoprotein	0.39	2.27E-02	2
66878	*Riok3*	RIO kinase 3	0.39	5.84E-03	2
268697	*Ccnb1*	cyclin B1	0.39	1.98E-02	2

17524	*Mpp1*	membrane protein, palmitoylated	0.39	3.96E-03	2
67579	*Cpeb4*	cytoplasmic polyadenylation element binding protein 4	0.40	1.33E-02	2
269261	*Rpl12*	ribosomal protein L12	0.40	2.33E-03	2
14793	*Cdca3*	cell division cycle associated 3	0.40	1.56E-02	2
68066	*Slc25a39*	solute carrier family 25, member 39	0.40	2.63E-02	2
67326	*1700037H04Rik*	RIKEN cDNA 1700037H04 gene	0.40	2.83E-02	2
72567	*Bclaf1*	BCL2-associated transcription factor 1	0.40	9.08E-03	2
66218	*Ndufb9*	NADH:ubiquinone oxidoreductase subunit B9	0.40	3.59E-03	2
56349	*Net1*	neuroepithelial cell transforming gene 1	0.40	4.76E-04	2
54217	*Rpl36*	ribosomal protein L36	0.40	3.96E-02	2
12531	*Cdc25b*	cell division cycle 25B	0.40	2.04E-02	2
114641	*Rpl31*	ribosomal protein L31	0.41	1.52E-02	2
76044	*Ncapg2*	non-SMC condensin II complex, subunit G2	0.41	1.73E-02	2
66477	*Atp5md*	ATP synthase membrane subunit DAPIT	0.41	9.58E-03	4
74334	*Ranbp10*	RAN binding protein 10	0.41	1.26E-02	2
665433	*H2ac23*	H2A clustered histone 23	0.41	2.27E-02	2
76808	*Rpl18a*	ribosomal protein L18A	0.41	4.29E-03	2
19824	*Trim10*	tripartite motif-containing 10	0.41	1.03E-02	2
14081	*Acsl1*	acyl-CoA synthetase long-chain family member 1	0.41	1.80E-02	6
56738	*Mocs1*	molybdenum cofactor synthesis 1	0.41	9.61E-03	2
27057	*Ncoa4*	nuclear receptor coactivator 4	0.41	1.22E-02	2
12534	*Cdk1*	cyclin-dependent kinase 1	0.42	1.13E-02	2
20084	*Rps18*	ribosomal protein S18	0.42	1.86E-02	2
67017	*Fam210b*	family with sequence similarity 210, member B	0.42	1.52E-02	2
216198	*Tcp11l2*	t-complex 11 (mouse) like 2	0.42	1.00E-02	2
56812	*Dnajb2*	DnaJ heat shock protein family (Hsp40) member B2	0.42	8.95E-03	2
19192	*Psme3*	proteaseome (prosome, macropain) activator subunit 3 (PA28 gamma, Ki)	0.42	8.48E-03	2
14630	*Gclm*	glutamate-cysteine ligase, modifier subunit	0.42	1.33E-02	2
218977	*Dlgap5*	DLG associated protein 5	0.42	1.95E-02	2
11363	*Acadl*	acyl-Coenzyme A dehydrogenase, long-chain	0.42	5.27E-03	2
11519	*Add2*	adducin 2 (beta)	0.43	3.64E-02	2
56199	*Abcb10*	ATP-binding cassette, sub-family B (MDR/TAP), member 10	0.43	2.37E-02	2
52502	*Carhsp1*	calcium regulated heat stable protein 1	0.43	5.60E-03	2
54141	*Spag5*	sperm associated antigen 5	0.43	1.68E-02	2
18022	*Nfe2*	nuclear factor, erythroid derived 2	0.43	4.64E-03	2
14211	*Smc2*	structural maintenance of chromosomes 2	0.43	1.12E-02	2
17254	*Slc3a2*	solute carrier family 3 (activators of dibasic and neutral amino acid transport), member 2	0.43	2.12E-03	2
13555	*E2f1*	E2F transcription factor 1	0.43	1.68E-02	2
12447	*Ccne1*	cyclin E1	0.43	1.91E-02	2
27388	*Ptdss2*	phosphatidylserine synthase 2	0.43	2.51E-03	2
67684	*Luc7l3*	LUC7-like 3 (S. cerevisiae)	0.44	9.63E-03	2
17067	*Ly6c1*	lymphocyte antigen 6 complex, locus C1	0.44	1.70E-02	4
56449	*Ybx3*	Y box protein 3	0.44	3.65E-03	2
13828	*Epb42*	erythrocyte membrane protein band 4.2	0.44	1.59E-02	2
67264	*Ndufb8*	NADH:ubiquinone oxidoreductase subunit B8	0.44	1.60E-03	4
67097	*Rps10*	ribosomal protein S10	0.44	4.24E-03	2
22275	*Urod*	uroporphyrinogen decarboxylase	0.44	2.73E-02	2
16571	*Kif4*	kinesin family member 4	0.44	3.05E-03	2
12234	*Btrc*	beta-transducin repeat containing protein	0.44	1.41E-03	2
23834	*Cdc6*	cell division cycle 6	0.44	1.20E-03	2
241525	*Ypel4*	yippee like 4	0.45	2.59E-02	2
12585	*Cdr2*	cerebellar degeneration-related 2	0.45	1.05E-02	2
81489	*Dnajb1*	DnaJ heat shock protein family (Hsp40) member B1	0.45	2.12E-03	2
232023	*Vopp1*	vesicular, overexpressed in cancer, prosurvival protein 1	0.45	9.33E-03	2
14629	*Gclc*	glutamate-cysteine ligase, catalytic subunit	0.45	8.18E-03	2
229474	*Fhdc1*	FH2 domain containing 1	0.45	1.67E-02	2
19951	*Rpl32*	ribosomal protein L32	0.45	4.03E-03	2
19944	*Rpl29*	ribosomal protein L29	0.45	1.03E-02	2
19245	*Ptp4a3*	protein tyrosine phosphatase 4a3	0.45	1.80E-03	2
15354	*Hmgb3*	high mobility group box 3	0.46	2.11E-02	2
27028	*Ermap*	erythroblast membrane-associated protein	0.46	9.63E-03	7
68166	*Spire1*	spire type actin nucleation factor 1	0.46	1.95E-02	2
66481	*Rps21*	ribosomal protein S21	0.46	4.45E-03	2
66447	*Mgst3*	microsomal glutathione S-transferase 3	0.46	9.84E-03	2
11991	*Hnrnpd*	heterogeneous nuclear ribonucleoprotein D	0.46	3.70E-02	2
67238	*Fam220a*	family with sequence similarity 220, member A	0.46	1.88E-02	2
14775	*Gpx1*	glutathione peroxidase 1	0.47	7.10E-03	2
67182	*Pdzk1ip1*	PDZK1 interacting protein 1	0.47	2.01E-02	2

21349	*Tal1*	T cell acute lymphocytic leukemia 1	0.47	3.03E-02	2
76425	*Gid8*	GID complex subunit 8	0.47	5.18E-03	2
13034	*Ctse*	cathepsin E	0.47	1.53E-02	2
225887	*Ndufs8*	NADH:ubiquinone oxidoreductase core subunit S8	0.47	1.68E-03	4
93739	*Gabarapl2*	gamma-aminobutyric acid (GABA) A receptor-associated protein-like 2	0.47	2.56E-03	2
16596	*Klf1*	Kruppel-like factor 1 (erythroid)	0.47	3.85E-02	2
231004	*Samd11*	sterile alpha motif domain containing 11	0.48	1.73E-02	2
69046	*Isca1*	iron-sulfur cluster assembly 1	0.48	6.05E-03	2
17151	*Ccndbp1*	cyclin D-type binding-protein 1	0.48	1.23E-02	6
13685	*Eif4ebp1*	eukaryotic translation initiation factor 4E binding protein 1	0.48	1.73E-02	2
20044	*Rps14*	ribosomal protein S14	0.48	7.82E-03	2
12349	*Car2*	carbonic anhydrase 2	0.48	1.47E-02	2
12177	*Bnip3l*	BCL2/adenovirus E1B interacting protein 3-like	0.48	5.48E-03	2
267019	*Rps15a*	ribosomal protein S15A	0.48	1.20E-03	2
68612	*Ube2c*	ubiquitin-conjugating enzyme E2C	0.48	5.55E-03	2
97165	*Hmgb2*	high mobility group box 2	0.48	6.21E-03	2
11733	*Ank1*	ankyrin 1, erythroid	0.49	2.51E-02	2
19044	*Ppox*	protoporphyrinogen oxidase	0.49	1.63E-02	2
66489	*Rpl35*	ribosomal protein L35	0.49	2.67E-03	2
70827	*Trak2*	trafficking protein, kinesin binding 2	0.49	9.41E-03	2
237898	*Usp32*	ubiquitin specific peptidase 32	0.49	2.90E-03	2
20454	*St3gal5*	ST3 beta-galactoside alpha-2,3-sialyltransferase 5	0.49	4.68E-03	2
56307	*Metap2*	methionine aminopeptidase 2	0.49	3.95E-03	2
56018	*Stard10*	START domain containing 10	0.50	1.83E-02	2
110052	*Dek*	DEK proto-oncogene (DNA binding)	0.50	4.42E-03	2
67671	*Rpl38*	ribosomal protein L38	0.50	2.06E-03	2
12892	*Cpox*	coproporphyrinogen oxidase	0.51	1.08E-02	2
17345	*Mki67*	antigen identified by monoclonal antibody Ki 67	0.51	1.43E-02	2
74645	*Tent5c*	terminal nucleotidyltransferase 5C	0.51	1.96E-02	2
20533	*Slc4a1*	solute carrier family 4 (anion exchanger), member 1	0.52	6.35E-03	2
76890	*Memo1*	mediator of cell motility 1	0.52	1.55E-02	2
211586	*Tfdp2*	transcription factor Dp 2	0.52	4.80E-03	2
192194	*Btnl10*	butyrophilin-like 10	0.53	1.42E-02	2
12864	*Cox6c*	cytochrome c oxidase subunit 6C	0.53	2.43E-03	2
11431	*Acp1*	acid phosphatase 1, soluble	0.54	4.39E-03	2
70026	*Tspo2*	translocator protein 2	0.54	1.20E-02	2
433375	*Creg1*	cellular repressor of E1A-stimulated genes 1	0.54	8.33E-05	2
269587	*Epb41*	erythrocyte membrane protein band 4.1	0.54	1.22E-02	2
21672	*Prdx2*	peroxiredoxin 2	0.54	7.43E-03	2
209837	*Slc38a5*	solute carrier family 38, member 5	0.54	7.51E-03	6
57255	*Cldn13*	claudin 13	0.55	2.37E-02	2
93966	*Hemgn*	hemogen	0.56	6.12E-03	2
72361	*Ces2g*	carboxylesterase 2G	0.56	9.06E-03	2
20617	*Snca*	synuclein, alpha	0.57	1.92E-02	2
108907	*Nusap1*	nucleolar and spindle associated protein 1	0.58	3.14E-04	2
15288	*Hmbs*	hydroxymethylbilane synthase	0.58	5.26E-03	2
20679	*Sox6*	SRY (sex determining region Y)-box 6	0.58	2.54E-03	2
15122	*Hba-a1*	hemoglobin alpha, adult chain 1	0.59	1.22E-02	2
232670	*Tspan33*	tetraspanin 33	0.59	1.16E-02	2
68193	*Rpl24*	ribosomal protein L24	0.59	1.51E-04	2
15368	*Hmox1*	heme oxygenase 1	0.59	3.89E-03	6
11983	*Atpif1*	ATPase inhibitory factor 1	0.59	6.88E-03	2
18670	*Abcb4*	ATP-binding cassette, sub-family B (MDR/TAP), member 4	0.59	2.13E-02	2
12144	*Blm*	Bloom syndrome, RecQ like helicase	0.60	1.00E-02	2
22042	*Tfrc*	transferrin receptor	0.61	1.92E-02	6
242705	*E2f2*	E2F transcription factor 2	0.62	1.66E-03	2
11826	*Aqp1*	aquaporin 1	0.62	1.36E-03	2
18712	*Pim1*	proviral integration site 1	0.62	3.66E-02	2
19652	*Rbm3*	RNA binding motif (RNP1, RRM) protein 3	0.62	2.89E-02	2
13830	*Stom*	stomatin	0.63	7.54E-05	2
57444	*Isg20*	interferon-stimulated protein	0.63	2.89E-03	2
21916	*Tmod1*	tropomodulin 1	0.65	3.01E-03	2
14934	*Gypa*	glycophorin A	0.66	1.63E-03	2
14151	*Fech*	ferrochelatase	0.66	3.44E-03	2
101488143	*Hbb-bt*	hemoglobin, beta adult t chain	0.66	1.63E-02	2
11656	*Alas2*	aminolevulinic acid synthase 2, erythroid	0.69	6.22E-03	2
56791	*Ube2l6*	ubiquitin-conjugating enzyme E2L 6	0.70	1.86E-03	2
107569	*Nt5c3*	5'-nucleotidase, cytosolic III	0.70	3.97E-04	2

103711	*Pnpo*	pyridoxine 5'-phosphate oxidase	0.70	6.51E-03	2
78892	*Crispld2*	cysteine-rich secretory protein LCCL domain containing 2	0.79	1.37E-02	7
100503605	*Hbb-bs*	hemoglobin, beta adult s chain	0.92	2.79E-04	2
19743	*Rhag*	Rhesus blood group-associated A glycoprotein	0.92	6.56E-04	2
12491	*Cd36*	CD36 molecule	0.98	7.86E-05	2
70564	*Prxl2a*	peroxiredoxin like 2A	1.01	4.83E-04	2
11770	*Fabp4*	fatty acid binding protein 4, adipocyte	1.31	4.50E-03	6
12346	*Car1*	carbonic anhydrase 1	1.33	7.16E-03	2

SUPPLEMENTAL TABLE 4

Supplemental Table 4. Pathway enrichment analysis results – based on genes selectively downregulated in WT but not Sarm1-KO T1D as listed in Supplemental Table 3. Results from ShinyGO version 0.77, analyses including GO:Biological Process, GO:Molecular Function, KEGG, and Reactome. FDR=false discovery rate.

Analysis	FDR	nGenes	Pathway Genes	Fold Enrichment	Pathway	Genes
GO:Biological Process	9.48E-11	36	1900	4.02	Tissue development	Mmp14 Col1a1 Slc39a13 Klf4 Ptgis Plod1 Atp2b1 Dab2 Col2a1 Cdkn1a Lrp5 Fasn Nrp2 Stat1 Col5a1 Sema6d Lrp4 Tnc Ibsp Bmp3 Ncor2 Col1a2 Fgfr2 Fgfr1 Pth1r Pkd1 Satb2 Runx2 Sh3pxd2b Cd109 Phldb1 Lamb2 Phex Palld Ano6 Fat1
GO:Biological Process	5.41E-09	17	383	9.04	Ossification	Mmp14 Col1a1 Atp2b1 Col2a1 Lrp5 Lrp4 Ibsp Bmp3 Col1a2 Fgfr1 Pth1r Satb2 Runx2 Sh3pxd2b Phex Col13a1 Ano6
GO:Biological Process	4.95E-08	14	286	10.10	Extracellular matrix organization	Mmp14 Col1a1 Col2a1 Adamts10 Col5a1 Emilin1 Ibsp Fscn1 Col1a2 Antxr1 Sh3pxd2b Phldb1 Lamb2 Col13a1
GO:Biological Process	4.95E-08	14	288	10.07	Extracellular structure organization	Mmp14 Col1a1 Col2a1 Adamts10 Col5a1 Emilin1 Ibsp Fscn1 Col1a2 Antxr1 Sh3pxd2b Phldb1 Lamb2 Col13a1
GO:Biological Process	4.95E-08	14	287	10.07	External encapsulating structure organization	Mmp14 Col1a1 Col2a1 Adamts10 Col5a1 Emilin1 Ibsp Fscn1 Col1a2 Antxr1 Sh3pxd2b Phldb1 Lamb2 Col13a1
GO:Biological Process	1.01E-06	16	508	6.44	Skeletal system development	Mmp14 Col1a1 Col2a1 Lrp5 Bmp3 Col1a2 Fgfr2 Fgfr1 Pth1r Pkd1 Satb2 Runx2 Sh3pxd2b Phex Col13a1 Ano6
GO:Biological Process	1.01E-06	23	1135	4.18	Circulatory system development	Mmp14 Col1a1 Klf4 Cad Sash1 Ptgis Col2a1 Cdkn1a Lrp5 Nrp2 Stat1 Col5a1 Emilin1 Ncor2 Col1a2 Fgfr1 Pkd1 Antxr1 Sh3pxd2b Stab1 Tcf4 Mia3 Gtf2i
GO:Biological Process	2.41E-06	18	717	5.16	Blood vessel development	Mmp14 Col1a1 Klf4 Sash1 Ptgis Lrp5 Nrp2 Stat1 Col5a1 Emilin1 Col1a2 Fgfr1 Pkd1 Antxr1 Stab1 Tcf4 Mia3 Gtf2i
GO:Biological Process	2.41E-06	12	274	8.99	Connective tissue development	Col1a1 Slc39a13 Col2a1 Lrp5 Col5a1 Bmp3 Fgfr1 Pth1r Pkd1 Satb2 Runx2 Sh3pxd2b
GO:Biological Process	3.69E-06	21	1041	4.19	Animal organ morphogenesis	Mmp14 Col1a1 Dab2 Col2a1 Lrp5 Nrp2 Col5a1 Lrp4 Tnc Ncor2 Col1a2 Fgfr2 Fgfr1 Pkd1 Satb2 Runx2 Sh3pxd2b Lamb2 Phex Col13a1 Fat1
GO:Biological Process	3.69E-06	18	747	4.94	Vasculature development	Mmp14 Col1a1 Klf4 Sash1 Ptgis Lrp5 Nrp2 Stat1 Col5a1 Emilin1 Col1a2 Fgfr1 Pkd1 Antxr1 Stab1 Tcf4 Mia3 Gtf2i
GO:Biological Process	1.17E-05	23	1362	3.56	Cell adhesion	Mmp14 Col1a1 Klf4 Myo10 Cd200 Srgap2 Col5a1 Tnc Mxra8 Emilin1 Ibsp Pkd1 Antxr1 Lrp1 Kirrel Stab1 Lamb2 Mia3 Palld Col13a1 Fat1 H2-Ab1 Pcdhgc3
GO:Biological Process	1.26E-05	23	1374	3.53	Biological adhesion	Mmp14 Col1a1 Klf4 Myo10 Cd200 Srgap2 Col5a1 Tnc Mxra8 Emilin1 Ibsp Pkd1 Antxr1 Lrp1 Kirrel Stab1 Lamb2 Mia3 Palld Col13a1 Fat1 H2-Ab1 Pcdhgc3
GO:Biological Process	3.83E-05	16	694	4.72	Response to growth factor	Col1a1 Klf4 Cad Fyn Dab2 Col2a1 Nrp2 Tnc Emilin1 Ibsp Col1a2 Fgfr2 Fgfr1 Runx2 Cd109 Tcf4
GO:Biological Process	4.16E-05	10	237	8.60	Skeletal system morphogenesis	Mmp14 Col1a1 Col2a1 Lrp5 Fgfr2 Pkd1 Satb2 Runx2 Sh3pxd2b Col13a1
GO:Biological Process	4.27E-05	6	53	22.39	Collagen fibril organization	Col1a1 Col2a1 Col5a1 Emilin1 Col1a2 Col13a1
GO:Biological Process	4.88E-05	5	31	32.96	Endochondral ossification	Mmp14 Col1a1 Col2a1 Runx2 Col13a1
GO:Biological Process	4.88E-05	5	31	32.96	Replacement ossification	Mmp14 Col1a1 Col2a1 Runx2 Col13a1
GO:Biological Process	4.88E-05	24	1636	3.08	Cell motility	Mmp14 Col1a1 Klf4 Nav1 Sash1 Pltp Fyn Dab2 Myo10 Lrp5 Gbf1 Nrp2 Srgap2 Col5a1 Sema6d Emilin1 Fscn1 Fgfr1 Satb2 Lrp1 Lamb2 Mia3 Palld Ano6
GO:Biological Process	4.88E-05	24	1636	3.08	Localization of cell	Mmp14 Col1a1 Klf4 Nav1 Sash1 Pltp Fyn Dab2 Myo10 Lrp5 Gbf1 Nrp2 Srgap2 Col5a1 Sema6d Emilin1 Fscn1 Fgfr1 Satb2 Lrp1 Lamb2 Mia3 Palld Ano6

GO:Biological Process	6.81E-05	18	937	3.90	Regulation of anatomical structure morphogenesis	Klf4 Sash1 Ptgis Fyn Epb41l2 Dab2 Myo10 Stat1 Sema6d Lrp4 Emilin1 Fgfr1 Runx2 Lrp1 Stab1 Phldb1 Tcf4 Gtf2i
GO:Biological Process	8.43E-05	15	666	4.61	Cellular response to growth factor stimulus	Col1a1 Klf4 Cad Fyn Dab2 Col2a1 Nrp2 Emilin1 Ibsp Col1a2 Fgfr2 Fgfr1 Runx2 Cd109 Tcf4
GO:Biological Process	0.000118413	4	16	49.44	Osteoblast development	Lrp5 Pth1r Satb2 Runx2
GO:Biological Process	0.000118656	19	1111	3.54	Tube development	Mmp14 Klf4 Sash1 Ptgis Col2a1 Cdkn1a Lrp5 Nrp2 Stat1 Tnc Emilin1 Ncor2 Fgfr1 Pkd1 Stab1 Tcf4 Mia3 Phex Gtf2i
GO:Biological Process	0.000123576	22	1474	3.10	Cell migration	Mmp14 Col1a1 Klf4 Nav1 Sash1 Fyn Dab2 Lrp5 Gbf1 Nrp2 Srgap2 Col5a1 Sema6d Emilin1 Fscn1 Fgfr1 Satb2 Lrp1 Lamb2 Mia3 Palld Ano6
GO:Biological Process	0.000131458	9	222	8.32	Bone development	Mmp14 Col1a1 Col2a1 Lrp5 Runx2 Sh3pxd2b Phex Col13a1 Ano6
GO:Biological Process	0.000231732	5	43	22.99	Extracellular matrix assembly	Emilin1 Col1a2 Antxr1 Phldb1 Lamb2
GO:Biological Process	0.000232181	24	1822	2.78	Locomotion	Mmp14 Col1a1 Klf4 Nav1 Sash1 Pltp Fyn Dab2 Myo10 Lrp5 Gbf1 Nrp2 Srgap2 Col5a1 Sema6d Emilin1 Fscn1 Fgfr1 Satb2 Lrp1 Lamb2 Mia3 Palld Ano6
GO:Biological Process	0.000381911	3	7	84.75	Protein localization to endoplasmic reticulum exit site	Gbf1 Sec16a Mia3
GO:Biological Process	0.000462201	18	1164	3.32	Epithelium development	Mmp14 Klf4 Dab2 Col2a1 Cdkn1a Lrp5 Fasn Stat1 Col5a1 Lrp4 Tnc Ncor2 Fgfr1 Pkd1 Cd109 Lamb2 Palld Fat1
GO:Biological Process	0.000473198	8	199	8.24	Cartilage development	Col1a1 Col2a1 Bmp3 Fgfr1 Pth1r Pkd1 Satb2 Runx2
GO:Biological Process	0.000494744	8	199	8.15	Osteoblast differentiation	Col1a1 Lrp5 Bmp3 Fgfr1 Pth1r Satb2 Runx2 Sh3pxd2b
GO:Biological Process	0.000604646	18	1144	3.23	Anatomical structure formation involved in morphogenesis	Mmp14 Col1a1 Klf4 Sash1 Ptgis Col2a1 Nrp2 Stat1 Col5a1 Emilin1 Fgfr2 Fgfr1 Stab1 Cd109 Phldb1 Tcf4 Mia3 Gtf2i
GO:Biological Process	0.000604646	6	98	12.62	Bone morphogenesis	Mmp14 Col1a1 Col2a1 Lrp5 Runx2 Col13a1
GO:Biological Process	0.000637734	12	527	4.65	Angiogenesis	Mmp14 Klf4 Sash1 Ptgis Nrp2 Stat1 Emilin1 Fgfr1 Stab1 Tcf4 Mia3 Gtf2i
GO:Biological Process	0.000637734	18	1169	3.20	Embryo development	Mmp14 Col1a1 Klf4 Col2a1 Lrp5 Nrp2 Col5a1 Lrp4 Ncor2 Fgfr2 Fgfr1 Pth1r Pkd1 Satb2 Runx2 Sec24d Phldb1 Col13a1
GO:Biological Process	0.000637734	7	155	9.35	Biomineral tissue development	Col1a1 Atp2b1 Ibsp Col1a2 Pth1r Phex Ano6
GO:Biological Process	0.000637734	6	101	12.36	Collagen metabolic process	Mmp14 Col1a1 Mrc2 Col5a1 Emilin1 Col1a2
GO:Biological Process	0.000637734	13	623	4.27	Blood vessel morphogenesis	Mmp14 Klf4 Sash1 Ptgis Lrp5 Nrp2 Stat1 Emilin1 Fgfr1 Stab1 Tcf4 Mia3 Gtf2i
GO:Biological Process	0.000637734	4	28	29.30	COPII-coated vesicle budding	Sec16a Sec31a Sec24d Mia3
GO:Biological Process	0.000679264	7	157	9.23	Biomineralization	Col1a1 Atp2b1 Ibsp Col1a2 Pth1r Phex Ano6
GO:Biological Process	0.000727146	5	60	16.76	Endochondral bone morphogenesis	Mmp14 Col1a1 Col2a1 Runx2 Col13a1
GO:Biological Process	0.000771737	5	61	16.48	Vesicle budding from membrane	Gbf1 Sec16a Sec31a Sec24d Mia3
GO:Biological Process	0.000836354	6	109	11.41	Negative regulation of angiogenesis	Klf4 Stat1 Emilin1 Stab1 Tcf4 Gtf2i
GO:Biological Process	0.000911995	6	111	11.19	Negative regulation of blood vessel morphogenesis	Klf4 Stat1 Emilin1 Stab1 Tcf4 Gtf2i
GO:Biological Process	0.000941371	6	112	11.09	Negative regulation of vasculature development	Klf4 Stat1 Emilin1 Stab1 Tcf4 Gtf2i
GO:Biological Process	0.000945491	4	33	25.52	Cellular response to vitamin	Col1a1 Atp2b1 Tnc Phex
GO:Biological Process	0.001231118	6	116	10.50	Endoplasmic reticulum to Golgi vesicle-mediated transport	Kdelr1 Sec16a Sec31a Sec24d Lman1 Mia3
GO:Biological Process	0.001231154	15	887	3.46	Tube morphogenesis	Mmp14 Klf4 Sash1 Ptgis Lrp5 Nrp2 Stat1 Tnc Emilin1 Fgfr1 Pkd1 Stab1 Tcf4 Mia3 Gtf2i
GO:Biological Process	0.001242521	6	116	10.41	Bone mineralization	Atp2b1 Ibsp Col1a2 Pth1r Phex Ano6
GO:Biological Process	0.001685443	11	511	4.47	Response to wounding	Col1a1 Klf4 Cdkn1a Col5a1 Tnc Fgfr2 Lrp1 Cd109 Lamb2 Mia3 Ano6

GO:Biological Process	0.001702589	3	13	45.64	Regulation of extracellular matrix assembly	Emilin1 Antxr1 Phldb1
GO:Biological Process	0.001768684	7	188	7.65	Gastrulation	Mmp14 Klf4 Lrp5 Col5a1 Fgfr2 Fgfr1 Phldb1
GO:Biological Process	0.00197275	20	1606	2.65	Response to endogenous stimulus	Mmp14 Col1a1 Klf4 Cad Fyn Atp2b1 Dab2 Col2a1 Cdkn1a Lrp5 Tnc Emilin1 Ncor2 Col1a2 Fgfr2 Fgfr1 Runx2 Cd109 Mia3 Phex
GO:Biological Process	0.002149671	8	265	6.16	Golgi vesicle transport	Kdelr1 Gbf1 Sec16a Mon2 Sec31a Sec24d Lman1 Mia3
GO:Biological Process	0.002149671	6	132	9.27	Embryonic skeletal system development	Mmp14 Col1a1 Col2a1 Fgfr2 Satb2 Runx2
GO:Biological Process	0.00245304	3	16	39.55	COPII-coated vesicle cargo loading	Sec31a Sec24d Mia3
GO:Biological Process	0.002612445	4	43	18.83	Regulation of extracellular matrix organization	Emilin1 Fscn1 Antxr1 Phldb1
GO:Biological Process	0.002820691	4	45	18.40	Cellular response to nutrient	Col1a1 Atp2b1 Tnc Phex
GO:Biological Process	0.003457388	3	18	34.90	Vesicle cargo loading	Sec31a Sec24d Mia3
GO:Biological Process	0.003599249	8	289	5.65	Regulation of angiogenesis	Klf4 Sash1 Ptgis Stat1 Emilin1 Stab1 Tcf4 Gtf2i
GO:Biological Process	0.003768261	18	1405	2.69	Regulation of multicellular organismal development	Mmp14 Klf4 Sash1 Ptgis Atp2b1 Stat1 Sema6d Lrp4 Emilin1 Fgfr1 Runx2 Lrp1 Stab1 Cd109 Phldb1 Tcf4 Gtf2i Ano6
GO:Biological Process	0.003846056	8	293	5.57	Regulation of vasculature development	Klf4 Sash1 Ptgis Stat1 Emilin1 Stab1 Tcf4 Gtf2i
GO:Biological Process	0.003875362	3	20	32.96	Negative regulation of cytosolic calcium ion concentration	Atp2b1 Lrp1 Gtf2i
GO:Biological Process	0.004524303	7	224	6.38	Mesenchymal cell differentiation	Col1a1 Dab2 Nrp2 Sema6d Fgfr2 Fgfr1 Phldb1
GO:Biological Process	0.004584931	7	227	6.35	Regeneration	Klf4 Cad Cdkn1a Tnc Lrp1 Lamb2 Lifr
GO:Biological Process	0.004807001	5	98	10.30	Embryonic skeletal system morphogenesis	Mmp14 Col2a1 Fgfr2 Satb2 Runx2
GO:Biological Process	0.005220759	5	104	10.09	Response to fibroblast growth factor	Col1a1 Tnc Fgfr2 Fgfr1 Runx2
GO:Biological Process	0.005334599	7	229	6.15	Cellular response to transforming growth factor beta stimulus	Col1a1 Fyn Dab2 Emilin1 Col1a2 Fgfr2 Cd109
GO:Biological Process	0.005351849	7	276	6.13	Skin development	Col1a1 Col5a1 Lrp4 Col1a2 Pkd1 Cd109 Palld
GO:Biological Process	0.005351849	4	56	14.92	Cellular response to retinoic acid	Col1a1 Klf4 Tnc Fgfr2
GO:Biological Process	0.005553388	3	22	28.25	Cellular response to vitamin D	Atp2b1 Tnc Phex
GO:Biological Process	0.005621766	7	233	6.04	Response to transforming growth factor beta	Col1a1 Fyn Dab2 Emilin1 Col1a2 Fgfr2 Cd109
GO:Biological Process	0.006150512	3	23	26.97	Positive regulation of extracellular matrix organization	Emilin1 Fscn1 Phldb1
GO:Biological Process	0.006150512	3	22	26.97	Podosome assembly	Fscn1 Sh3pxd2b Palld
GO:Biological Process	0.006199298	4	56	14.13	Axon regeneration	Klf4 Tnc Lrp1 Lamb2
GO:Biological Process	0.007877037	11	632	3.58	Embryonic morphogenesis	Mmp14 Klf4 Col2a1 Lrp5 Col5a1 Lrp4 Fgfr2 Fgfr1 Satb2 Runx2 Phldb1
GO:Biological Process	0.008580741	11	639	3.54	Tissue morphogenesis	Mmp14 Klf4 Dab2 Lrp5 Nrp2 Col5a1 Tnc Fgfr2 Fgfr1 Pkd1 Fat1
GO:Biological Process	0.008711817	12	765	3.29	Chordate embryonic development	Mmp14 Col1a1 Klf4 Col2a1 Ncor2 Fgfr2 Fgfr1 Pth1r Pkd1 Satb2 Runx2 Sec24d
GO:Biological Process	0.009593787	3	26	22.82	Establishment of monopolar cell polarity	Gbf1 Fscn1 Fat1
GO:Biological Process	0.009757752	4	67	12.36	Protein localization to endoplasmic reticulum	Kdelr1 Gbf1 Sec16a Mia3
GO:Biological Process	0.010121111	12	781	3.22	Embryo development ending in birth or egg hatching	Mmp14 Col1a1 Klf4 Col2a1 Ncor2 Fgfr2 Fgfr1 Pth1r Pkd1 Satb2 Runx2 Sec24d
GO:Biological Process	0.010121111	4	65	12.17	Neuron projection regeneration	Klf4 Tnc Lrp1 Lamb2
GO:Biological Process	0.010121111	8	351	4.63	Cell-substrate adhesion	Mmp14 Col1a1 Srgap2 Emilin1 Antxr1 Lrp1 Lamb2 Col13a1
GO:Biological Process	0.010121111	3	27	21.97	Establishment or maintenance of monopolar cell polarity	Gbf1 Fscn1 Fat1

GO:Biological Process	0.010935308	13	907	2.99	Enzyme linked receptor protein signaling pathway	Col1a1 Fyn Dab2 Nrp2 Emilin1 Bmp3 Col1a2 Fgfr2 Fgfr1 Runx2 Cd109 Tcf4 Lifr
GO:Biological Process	0.010935534	7	282	5.26	Response to alcohol	Klf4 Cad Fyn Atp2b1 Cdkn1a Tnc Fgfr2
GO:Biological Process	0.010935534	2	6	65.92	Osteoblast fate commitment	Runx2 Sh3pxd2b
GO:Biological Process	0.011074573	5	127	8.10	Odontogenesis	Col1a1 Lrp4 Tnc Runx2 Phex
GO:Biological Process	0.011084719	4	69	11.63	Cranial skeletal system development	Mmp14 Fgfr2 Runx2 Sh3pxd2b
GO:Biological Process	0.011290385	13	998	2.96	Negative regulation of developmental process	Klf4 Cdkn1a Lrp5 Stat1 Col5a1 Sema6d Lrp4 Emilin1 Ncor2 Fgfr1 Stab1 Tcf4 Gtf2i
GO:Biological Process	0.011607187	7	278	5.17	Mesenchyme development	Col1a1 Dab2 Nrp2 Sema6d Fgfr2 Fgfr1 Phldb1
GO:Biological Process	0.011837578	16	1323	2.55	Cellular response to endogenous stimulus	Col1a1 Klf4 Cad Fyn Atp2b1 Dab2 Col2a1 Tnc Emilin1 Ncor2 Col1a2 Fgfr2 Fgfr1 Runx2 Cd109 Phex
GO:Biological Process	0.012210353	12	805	3.11	Head development	Mmp14 Col1a1 Fyn Atp2b1 Col2a1 Nrp2 Srgap2 Sema6d Ncor2 Fgfr1 Satb2 Lrp1
GO:Biological Process	0.012736089	8	377	4.39	Wound healing	Col1a1 Cdkn1a Col5a1 Tnc Fgfr2 Cd109 Mia3 Ano6
GO:Biological Process	0.013702323	2	7	56.50	Amyloid-beta clearance by cellular catabolic process	Lrp4 Lrp1
GO:Biological Process	0.013702323	6	210	5.96	Lung development	Mmp14 Tnc Ncor2 Fgfr1 Pkd1 Phex
GO:Biological Process	0.013702323	2	7	56.50	Axon extension involved in regeneration	Lrp1 Lamb2
GO:Biological Process	0.013714283	7	284	4.96	Negative regulation of cell adhesion	Mmp14 Col1a1 Klf4 Tnc Lrp1 Mia3 H2-Ab1
GO:Biological Process	0.014320579	6	213	5.87	Respiratory tube development	Mmp14 Tnc Ncor2 Fgfr1 Pkd1 Phex
GO:Biological Process	0.014711743	10	604	3.48	Sensory organ development	Klf4 Atp2b1 Col2a1 Lrp5 Col5a1 Fgfr1 Sh3pxd2b Lamb2 H2-K1 Fat1
GO:Biological Process	0.014767438	10	644	3.48	Epithelial cell differentiation	Klf4 Dab2 Cdkn1a Fasn Ncor2 Fgfr1 Cd109 Lamb2 Palld Fat1
GO:Biological Process	0.015242369	3	35	17.98	Response to vitamin D	Atp2b1 Tnc Phex
GO:Biological Process	0.01549771	10	601	3.45	Heart development	Cad Col2a1 Cdkn1a Nrp2 Col5a1 Emilin1 Ncor2 Fgfr1 Pkd1 Sh3pxd2b
GO:Biological Process	0.016391825	2	8	49.44	Positive regulation of extracellular matrix assembly	Emilin1 Phldb1
GO:Biological Process	0.016391825	19	1765	2.22	Neurogenesis	Mmp14 Klf4 Nav1 Fyn Nrp2 Srgap2 Sema6d Lrp4 Tnc Mxra8 Fgfr1 Satb2 Runx2 Lrp1 Lamb2 Tcf4 Lifr Palld H2-K1
GO:Biological Process	0.016391825	11	710	3.15	Regulation of cell adhesion	Mmp14 Col1a1 Klf4 Myo10 Tnc Emilin1 Ibsp Pkd1 Lrp1 Mia3 H2-Ab1
GO:Biological Process	0.016391825	2	8	49.44	Sprouting of injured axon	Lrp1 Lamb2
GO:Biological Process	0.016391825	2	8	49.44	Mesenchymal-epithelial cell signaling	Tnc Fgfr1
GO:Biological Process	0.01682117	4	85	9.89	Endoplasmic reticulum organization	Sec16a Sec31a Lman1 Mia3
GO:Biological Process	0.017491549	11	728	3.12	Supramolecular fiber organization	Col1a1 Col2a1 Col5a1 Emilin1 Fscn1 Col1a2 Sh3pxd2b Kirrel Mical3 Col13a1 Fat1
GO:Biological Process	0.017569696	6	220	5.52	Morphogenesis of a branching structure	Mmp14 Lrp5 Tnc Fgfr1 Pkd1 Col13a1
GO:Biological Process	0.017569696	5	146	6.96	Epithelial to mesenchymal transition	Col1a1 Dab2 Fgfr2 Fgfr1 Phldb1
GO:Biological Process	0.018176788	16	1380	2.41	Positive regulation of developmental process	Mmp14 Col1a1 Klf4 Sash1 Ptgis Atp2b1 Dab2 Lrp5 Stat1 Fgfr1 Runx2 Lrp1 Sh3pxd2b Phldb1 Tcf4 Ano6
GO:Biological Process	0.018974676	11	739	3.08	Cell junction organization	Mmp14 Fyn Nrp2 Lrp4 Tnc Fscn1 Lrp1 Lamb2 Septin11 Palld Pcdhgc3
GO:Biological Process	0.01909448	17	1610	2.31	Regulation of cell differentiation	Mmp14 Col1a1 Klf4 Dab2 Lrp5 Stat1 Col5a1 Sema6d Lrp4 Ncor2 Fgfr1 Runx2 Lrp1 Sh3pxd2b Cd109 Phldb1 Tcf4
GO:Biological Process	0.01954602	2	9	43.95	Negative regulation of extracellular matrix organization	Emilin1 Antxr1
GO:Biological Process	0.019694215	4	87	9.31	Response to axon injury	Klf4 Tnc Lrp1 Lamb2
GO:Biological Process	0.021122041	6	238	5.27	Respiratory system development	Mmp14 Tnc Ncor2 Fgfr1 Pkd1 Phex

GO:Biological Process	0.021362811	5	156	6.55	Appendage morphogenesis	Col2a1 Lrp5 Lrp4 Fgfr1 Runx2
GO:Biological Process	0.021362811	5	156	6.55	Limb morphogenesis	Col2a1 Lrp5 Lrp4 Fgfr1 Runx2
GO:Biological Process	0.021362811	14	1112	2.56	Positive regulation of cellular component organization	Fyn Dab2 Lrp5 Lrp4 Emilin1 Fscn1 Fgfr1 Lrp1 Sh3pxd2b Kirrel Lman1 Phldb1 Palld Ano6
GO:Biological Process	0.022666633	13	1014	2.66	Central nervous system development	Fyn Atp2b1 Col2a1 Nrp2 Srgap2 Sema6d Mxra8 Ncor2 Fgfr1 Pkd1 Satb2 Lrp1 Lamb2
GO:Biological Process	0.024063393	9	534	3.46	Morphogenesis of an epithelium	Mmp14 Klf4 Dab2 Lrp5 Col5a1 Tnc Fgfr1 Pkd1 Fat1
GO:Biological Process	0.024072168	6	243	5.09	Epithelial cell development	Cdkn1a Fasn Fgfr1 Lamb2 Palld Fat1
GO:Biological Process	0.024184897	8	425	3.81	Ameboidal-type cell migration	Klf4 Sash1 Lrp5 Nrp2 Srgap2 Sema6d Fgfr1 Mia3
GO:Biological Process	0.024392084	18	1710	2.17	Cellular protein localization	Mmp14 Col1a1 Kdelr1 Fyn Epb41l2 Dab2 Cdkn1a Gbf1 Sec16a Lrp4 Atp1b3 Pkd1 Sec31a Sec24d Lrp1 Sh3pxd2b Mia3 Septin11
GO:Biological Process	0.024392084	4	97	8.60	Cellular response to fibroblast growth factor stimulus	Col1a1 Fgfr2 Fgfr1 Runx2
GO:Biological Process	0.02619367	18	1722	2.16	Cellular macromolecule localization	Mmp14 Col1a1 Kdelr1 Fyn Epb41l2 Dab2 Cdkn1a Gbf1 Sec16a Lrp4 Atp1b3 Pkd1 Sec31a Sec24d Lrp1 Sh3pxd2b Mia3 Septin11
GO:Biological Process	0.026699547	2	11	35.96	Skin morphogenesis	Col1a1 Col1a2
GO:Biological Process	0.027737274	3	45	13.48	Regulation of extrinsic apoptotic signaling pathway in absence of ligand	Fyn Col2a1 Fgfr1
GO:Biological Process	0.027737274	9	592	3.36	Cellular response to lipid	Col1a1 Klf4 Sash1 Atp2b1 Dab2 Tnc Ncor2 Fgfr2 Phex
GO:Biological Process	0.028831985	9	549	3.33	Positive regulation of cell migration	Mmp14 Col1a1 Sash1 Dab2 Sema6d Fgfr1 Lrp1 Mia3 Ano6
GO:Biological Process	0.029168667	3	47	13.18	Metal ion export	Atp2b1 Atp1b3 Ano6
GO:Biological Process	0.029220855	4	105	8.07	Response to vitamin	Col1a1 Atp2b1 Tnc Phex
GO:Biological Process	0.029994472	16	1456	2.26	Vesicle-mediated transport	Kdelr1 Mrc2 Dab2 Lrp5 Gbf1 Sec16a Lrp4 Mon2 Sec31a Sec24d Lrp1 Lman1 Stab1 Mical3 Mia3 Ano6
GO:Biological Process	0.030083033	12	918	2.68	Regulation of cell migration	Mmp14 Col1a1 Klf4 Sash1 Dab2 Srgap2 Sema6d Emilin1 Fgfr1 Lrp1 Mia3 Ano6
GO:Biological Process	0.030083033	2	12	32.96	Basement membrane assembly	Phldb1 Lamb2
GO:Biological Process	0.031007147	11	821	2.82	Cell-cell adhesion	Klf4 Myo10 Cd200 Pkd1 Kirrel Mia3 Palld Col13a1 Fat1 H2-Ab1 Pcdhgc3
GO:Biological Process	0.031007147	5	180	5.82	Neuron migration	Nav1 Fyn Nrp2 Srgap2 Satb2
GO:Biological Process	0.031007147	16	1470	2.25	Neuron differentiation	Klf4 Fyn Nrp2 Srgap2 Sema6d Lrp4 Tnc Fgfr1 Satb2 Runx2 Lrp1 Lamb2 Tcf4 Lifr Palld H2-K1
GO:Biological Process	0.031007147	4	107	7.83	Chondrocyte differentiation	Col2a1 Fgfr1 Pth1r Runx2
GO:Biological Process	0.031007147	3	47	12.62	Embryonic cranial skeleton morphogenesis	Mmp14 Fgfr2 Runx2
GO:Biological Process	0.031150349	6	306	4.71	Epidermis development	Klf4 Plod1 Lrp4 Fgfr1 Cd109 Palld
GO:Biological Process	0.031519915	4	108	7.76	Response to retinoic acid	Col1a1 Klf4 Tnc Fgfr2
GO:Biological Process	0.031990161	13	1056	2.51	Cell morphogenesis	Fyn Epb41l2 Myo10 Nrp2 Srgap2 Sema6d Lrp4 Antxr1 Lrp1 Lamb2 Lifr Palld Fat1
GO:Biological Process	0.032579241	17	1627	2.16	Generation of neurons	Klf4 Nav1 Fyn Nrp2 Srgap2 Sema6d Lrp4 Tnc Fgfr1 Satb2 Runx2 Lrp1 Lamb2 Tcf4 Lifr Palld H2-K1
GO:Biological Process	0.032952077	13	1064	2.50	Neuron projection development	Klf4 Fyn Nrp2 Srgap2 Sema6d Lrp4 Tnc Fgfr1 Lrp1 Lamb2 Lifr Palld H2-K1
GO:Biological Process	0.033506499	9	573	3.21	Positive regulation of cell motility	Mmp14 Col1a1 Sash1 Dab2 Sema6d Fgfr1 Lrp1 Mia3 Ano6
GO:Biological Process	0.035343624	3	54	11.87	Collagen biosynthetic process	Col1a1 Col5a1 Emilin1
GO:Biological Process	0.03601798	8	478	3.48	Embryonic organ development	Mmp14 Col2a1 Fgfr2 Fgfr1 Pkd1 Satb2 Runx2 Col13a1

GO:Biological Process	0.036687027	2	14	28.25	Vesicle coating	Gbf1 Sec16a
GO:Biological Process	0.036687027	2	14	28.25	Negative regulation of keratinocyte proliferation	Fgfr2 Cd109
GO:Biological Process	0.036687027	3	55	11.63	Forebrain generation of neurons	Nrp2 Fgfr1 Satb2
GO:Biological Process	0.036687027	2	14	28.25	Positive regulation of podosome assembly	Fscn1 Palld
GO:Biological Process	0.037294101	16	1531	2.18	Plasma membrane bounded cell projection organization	Klf4 Fyn Myo10 Nrp2 Srgap2 Sema6d Lrp4 Tnc Fscn1 Fgfr1 Lrp1 Lamb2 Lifr Palld H2-K1 Ano6
GO:Biological Process	0.037294101	4	115	7.26	Formation of primary germ layer	Mmp14 Col5a1 Fgfr2 Fgfr1
GO:Biological Process	0.037294101	5	188	5.43	Appendage development	Col2a1 Lrp5 Lrp4 Fgfr1 Runx2
GO:Biological Process	0.037294101	5	188	5.43	Limb development	Col2a1 Lrp5 Lrp4 Fgfr1 Runx2
GO:Biological Process	0.037580695	9	590	3.12	Positive regulation of locomotion	Mmp14 Col1a1 Sash1 Dab2 Sema6d Fgfr1 Lrp1 Mia3 Ano6
GO:Biological Process	0.037580695	9	590	3.12	Positive regulation of cellular component movement	Mmp14 Col1a1 Sash1 Dab2 Sema6d Fgfr1 Lrp1 Mia3 Ano6
GO:Biological Process	0.03817237	12	978	2.55	Cell activation	Mmp14 Kdelr1 Impdh1 Fyn Cd200 Cdkn1a Gbf1 Emilin1 Fgfr1 Runx2 Tcf4 H2-Ab1
GO:Biological Process	0.03817237	8	472	3.40	Synapse organization	Fyn Nrp2 Lrp4 Tnc Lamb2 Septin11 Palld Pcdhgc3
GO:Biological Process	0.03840631	12	970	2.55	Regulation of cell motility	Mmp14 Col1a1 Klf4 Sash1 Dab2 Srgap2 Sema6d Emilin1 Fgfr1 Lrp1 Mia3 Ano6
GO:Biological Process	0.038695562	13	1124	2.43	Regulation of protein phosphorylation	Sash1 Fyn Dab2 Cdkn1a Prrc1 Lrp5 Lrp4 Emilin1 Bmp3 Fgfr1 Pkd1 Kirrel Cd109
GO:Biological Process	0.039126692	3	56	10.99	Export across plasma membrane	Atp2b1 Atp1b3 Ano6
GO:Biological Process	0.039126692	2	16	26.37	Golgi to endosome transport	Gbf1 Mon2
GO:Biological Process	0.039126692	14	1263	2.32	Regulation of phosphorylation	Klf4 Sash1 Fyn Dab2 Cdkn1a Prrc1 Lrp5 Lrp4 Emilin1 Bmp3 Fgfr1 Pkd1 Kirrel Cd109
GO:Biological Process	0.039126692	3	56	10.99	Positive regulation of ossification	Atp2b1 Runx2 Ano6
GO:Biological Process	0.039637797	10	726	2.85	Developmental growth	Cdkn1a Nrp2 Sema6d Lrp4 Tnc Fgfr2 Fgfr1 Lrp1 Sh3pxd2b Lamb2
GO:Biological Process	0.040701554	3	55	10.79	Neuromuscular junction development	Lrp4 Tnc Lamb2
GO:Biological Process	0.040701554	8	488	3.34	Axon development	Klf4 Nrp2 Sema6d Lrp4 Tnc Lrp1 Lamb2 Palld
GO:Biological Process	0.041614327	6	285	4.28	Regulation of cellular response to growth factor stimulus	Dab2 Emilin1 Fgfr1 Runx2 Cd109 Tcf4
GO:Biological Process	0.042327206	2	16	24.72	Negative regulation of cell-substrate junction organization	Mmp14 Lrp1
GO:Biological Process	0.042327206	2	16	24.72	Negative regulation of focal adhesion assembly	Mmp14 Lrp1
GO:Biological Process	0.042327206	2	17	24.72	Negative regulation of androgen receptor signaling pathway	Dab2 Ncor2
GO:Biological Process	0.042667081	7	397	3.68	Eye development	Klf4 Atp2b1 Lrp5 Col5a1 Sh3pxd2b Lamb2 Fat1
GO:Biological Process	0.042675547	5	199	5.12	Regulation of protein localization to membrane	Mmp14 Fyn Dab2 Lrp4 Lrp1
GO:Biological Process	0.042675547	16	1570	2.12	Cell projection organization	Klf4 Fyn Myo10 Nrp2 Srgap2 Sema6d Lrp4 Tnc Fscn1 Fgfr1 Lrp1 Lamb2 Lifr Palld H2-K1 Ano6
GO:Biological Process	0.043299464	16	1588	2.12	Protein phosphorylation	Cad Sash1 Fyn Dab2 Cdkn1a Prrc1 Lrp5 Lrp4 Emilin1 Bmp3 Fgfr2 Fgfr1 Pkd1 Sik3 Kirrel Cd109
GO:Biological Process	0.043599508	7	400	3.65	Visual system development	Klf4 Atp2b1 Lrp5 Col5a1 Sh3pxd2b Lamb2 Fat1
GO:Biological Process	0.043933402	11	905	2.61	Response to hormone	Mmp14 Col1a1 Cad Fyn Atp2b1 Dab2 Cdkn1a Lrp5 Tnc Ncor2 Phex
GO:Biological Process	0.045694938	7	405	3.61	Sensory system development	Klf4 Atp2b1 Lrp5 Col5a1 Sh3pxd2b Lamb2 Fat1
GO:Biological Process	0.04619041	5	203	4.99	Morphogenesis of a branching epithelium	Mmp14 Lrp5 Tnc Fgfr1 Pkd1

GO:Biological Process	0.0469404	5	203	4.97	Negative regulation of cell projection organization	Fyn Sema6d Lrp4 Lrp1 H2-K1
GO:Biological Process	0.0470566 2	4	124	6.48	Regulation of ossification	Atp2b1 Lrp4 Runx2 Ano6
GO:Biological Process	0.0477336 2	3	61	9.89	Negative regulation of cell-substrate adhesion	Mmp14 Col1a1 Lrp1
GO:Biological Process	0.0491463 84	2	18	21.97	Regulation of chemokine (C-X-C motif) ligand 2 production	Klf4 Lrp1
GO:Biological Process	0.0491463 84	3	63	9.73	Glial cell migration	Mmp14 Srgap2 Lrp1
GO:Biological Process	0.0491463 84	12	1019	2.43	Regulation of locomotion	Mmp14 Col1a1 Klf4 Sash1 Dab2 Srgap2 Sema6d Emilin1 Fgfr1 Lrp1 Mia3 Ano6
GO:Biological Process	0.0491463 84	2	18	21.97	Regulation of podosome assembly	Fscn1 Palld
GO:Biological Process	0.0491463 84	2	18	21.97	Chemokine (C-X-C motif) ligand 2 production	Klf4 Lrp1
GO:Molecular Function	5.60E-05	4	12	65.92	Platelet-derived growth factor binding	Col1a1 Col2a1 Col5a1 Col1a2
GO:Molecular Function	5.60E-05	8	136	12.56	Extracellular matrix structural constituent	Col1a1 Col2a1 Col5a1 Tnc Emilin1 Col1a2 Lamb2 Col13a1
GO:Molecular Function	5.77E-05	8	147	11.55	Growth factor binding	Col1a1 Col2a1 Col5a1 Col1a2 Fgfr2 Fgfr1 Cd109 Lifr
GO:Molecular Function	0.0027108 26	4	35	22.60	Extracellular matrix structural constituent conferring tensile strength	Col1a1 Col5a1 Col1a2 Col13a1
GO:Molecular Function	0.0068706 26	8	299	5.61	Molecular adaptor activity	Klf4 Sash1 Dab2 Fscn1 Col1a2 Atp1b3 Sh3pxd2b Septin11
GO:Molecular Function	0.0099008 79	18	1457	2.58	Protein-containing complex binding	Mmp14 Pltp Fyn Epb41l2 Mrc2 Myo10 Cdkn1a Emilin1 Ibsp Ncor2 Fscn1 Fgfr1 Antxr1 Lrp1 Stab1 Lamb2 H2-K1 H2-Ab1
GO:Molecular Function	0.0132013 25	2	5	79.10	Fibroblast growth factor-activated receptor activity	Fgfr2 Fgfr1
GO:Molecular Function	0.0132013 25	10	612	3.83	Structural molecule activity	Col1a1 Epb41l2 Col2a1 Col5a1 Tnc Emilin1 Col1a2 Sec31a Lamb2 Col13a1
GO:Molecular Function	0.0165064 05	4	72	11.63	Cargo receptor activity	Dab2 Lrp1 Stab1 Mia3
GO:Molecular Function	0.0383131 37	3	39	15.21	Proteoglycan binding	Col2a1 Col5a1 Tnc
GO:Molecular Function	0.0469875 03	2	11	35.96	CD8 receptor binding	Fyn H2-K1
KEGG	3.85E-05	7	88	16.10	ECM-receptor interaction	Col1a1 Col2a1 Tnc Hspg2 Ibsp Col1a2 Lamb2
KEGG	0.0006399 81	6	108	11.99	Protein digestion and absorption	Col1a1 Col2a1 Col5a1 Col1a2 Atp1b3 Col13a1
KEGG	0.0006399 81	6	108	11.63	Parathyroid hormone synthesis, secretion and action	Mmp14 Cdkn1a Lrp5 Fgfr1 Pth1r Runx2
KEGG	0.0014625 1	7	200	7.10	Focal adhesion	Col1a1 Fyn Col2a1 Tnc Ibsp Col1a2 Lamb2
KEGG	0.0012685 94	9	349	5.51	Human papillomavirus infection	Col1a1 Col2a1 Cdkn1a Stat1 Tnc Ibsp Col1a2 Lamb2 H2-K1
KEGG	0.0012685 94	9	357	5.41	PI3K-Akt signaling pathway	Col1a1 Col2a1 Cdkn1a Tnc Ibsp Col1a2 Fgfr2 Fgfr1 Lamb2
Reactome	1.55E-05	5	27	36.62	MET activates PTK2 signaling	Col1a1 Col2a1 Col5a1 Col1a2 Lamb2
Reactome	1.55E-05	6	55	22.82	Collagen degradation	Mmp14 Col1a1 Col2a1 Col5a1 Col1a2 Col13a1
Reactome	1.55E-05	7	77	18.21	Integrin cell surface interactions	Col1a1 Col2a1 Col5a1 Tnc Ibsp Col1a2 Col13a1
Reactome	1.55E-05	11	284	8.00	Extracellular matrix organization	Mmp14 Col1a1 Plod1 Col2a1 Col5a1 Tnc Emilin1 Ibsp Col1a2 Lamb2 Col13a1
Reactome	4.35E-05	6	64	18.54	Collagen biosynthesis and modifying enzymes	Col1a1 Plod1 Col2a1 Col5a1 Col1a2 Col13a1
Reactome	5.65E-05	5	38	26.02	MET promotes cell motility	Col1a1 Col2a1 Col5a1 Col1a2 Lamb2
Reactome	7.14E-05	5	41	24.12	Collagen chain trimerization	Col1a1 Col2a1 Col5a1 Col1a2 Col13a1
Reactome	0.0001296 01	5	48	20.60	ECM proteoglycans	Col1a1 Col2a1 Col5a1 Tnc Col1a2
Reactome	0.0001296 01	6	85	13.96	Collagen formation	Col1a1 Plod1 Col2a1 Col5a1 Col1a2 Col13a1
Reactome	0.0002272 25	7	151	9.55	ER to Golgi Anterograde Transport	Kdelr1 Gbf1 Sec16a Sec31a Sec24d Lman1 Mia3
Reactome	0.0004764 85	3	11	53.93	Platelet Adhesion to exposed collagen	Col1a1 Fyn Col1a2

Reactome	0.000564071	4	36	21.97	Non-integrin membrane-ECM interactions	Col1a1 Col2a1 Col5a1 Col1a2
Reactome	0.000564071	5	74	13.73	Signaling by MET	Col1a1 Col2a1 Col5a1 Col1a2 Lamb2
Reactome	0.000564071	6	129	9.89	Degradation of the extracellular matrix	Mmp14 Col1a1 Col2a1 Col5a1 Col1a2 Col13a1
Reactome	0.000564071	7	181	8.00	Transport to the Golgi and subsequent modification	Kdelr1 Gbf1 Sec16a Sec31a Sec24d Lman1 Mia3
Reactome	0.00298901	5	190	9.51	Immunoregulatory interactions between a Lymphoid and a non-Lymphoid cell	Col1a1 Col2a1 Cd200 Col1a2 H2-K1
Reactome	0.003236188	4	58	13.64	Assembly of collagen fibrils and other multimeric structures	Col1a1 Col2a1 Col5a1 Col1a2
Reactome	0.004270959	7	270	5.47	Asparagine N-linked glycosylation	Kdelr1 Gbf1 Sec16a Sec31a Sec24d Lman1 Mia3
Reactome	0.005053373	4	68	11.81	COPII-mediated vesicle transport	Sec16a Sec31a Sec24d Lman1
Reactome	0.006636097	3	33	19.14	Cargo concentration in the ER	Sec24d Lman1 Mia3
Reactome	0.00797938	3	36	17.45	GPVI-mediated activation cascade	Col1a1 Fyn Col1a2
Reactome	0.00797938	10	663	3.42	Vesicle-mediated transport	Kdelr1 Dab2 Gbf1 Sec16a Sec31a Sec24d Lrp1 Lman1 Stab1 Mia3
Reactome	0.008183559	8	397	4.12	Signaling by Receptor Tyrosine Kinases	Col1a1 Fyn Col2a1 Nrp2 Col5a1 Col1a2 Fgfr1 Lamb2
Reactome	0.009352435	3	45	16.03	Antigen Presentation Folding assembly and peptide loading of class I MHC	Sec31a Sec24d H2-K1
Reactome	0.009712469	3	38	15.61	NCAM signaling for neurite out-growth	Fyn Col2a1 Col5a1
Reactome	0.011163037	2	10	39.55	Nephrin family interactions	Fyn Kirrel
Reactome	0.01177209	10	725	3.16	Adaptive Immune System	Col1a1 Fyn Mrc2 Col2a1 Cd200 Col1a2 Sec31a Sec24d H2-K1 H2-Ab1
Reactome	0.01510334	2	12	32.96	GP1b-IX-V activation signalling	Col1a1 Col1a2
Reactome	0.017853059	4	112	7.53	Post-translational protein phosphorylation	Tnc Mxra8 Lamb2 Mia3
Reactome	0.019593741	2	15	26.37	Crosslinking of collagen fibrils	Col1a1 Col1a2
Reactome	0.019593741	2	15	26.37	Anchoring fibril formation	Col1a1 Col1a2
Reactome	0.019593741	2	15	26.37	Nucleobase biosynthesis	Impdh1 Cad
Reactome	0.019593741	4	118	7.13	Reg. of Insulin-like Growth Factor IGF transport and uptake by Insulin-like Grow	Tnc Mxra8 Lamb2 Mia3
Reactome	0.019593741	4	182	7.06	Cell surface interactions at the vascular wall	Col1a1 Fyn Col1a2 Atp1b3
Reactome	0.023645275	7	532	3.63	Developmental Biology	Fyn Col2a1 Cdkn1a Col5a1 Sema6d Fgfr1 Tcf4
Reactome	0.036289253	2	21	18.83	NCAM1 interactions	Col2a1 Col5a1
Reactome	0.045906023	8	571	2.89	Membrane Trafficking	Kdelr1 Dab2 Gbf1 Sec16a Sec31a Sec24d Lman1 Mia3

SUPPLEMENTAL TABLE 5

Supplemental Table 5. Pathway enrichment analysis results – based on genes selectively <u>upregulated</u> in WT but not Sarm1-KO T1D as listed in Supplemental Table 3. Results from ShinyGO version 0.77, analyses including GO:Biological Process, GO:Molecular Function, KEGG, and Reactome. FDR=false discovery rate.

Analysis	Enrichment FDR	nGenes	Pathway Genes	Fold Enrichment	Pathway	Genes
GO:Biological Process	3.67E-10	17	139	11.49392	Erythrocyte homeostasis	Hmox1 Slc4a1 Epb42 Rhag Tspo2 Rps14 Alas2 Fam210b Tal1 Ank1 Ncapg2 Sox6 Klf1 Atpif1 Hmgb2 Hba-a1 Trim10
GO:Biological Process	6.83E-10	16	128	11.76977	Erythrocyte differentiation	Slc4a1 Epb42 Rhag Tspo2 Rps14 Alas2 Fam210b Tal1 Ank1 Ncapg2 Sox6 Klf1 Atpif1 Hmgb2 Hba-a1 Trim10
GO:Biological Process	1.93E-09	14	101	13.55073	Cytoplasmic translation	Cpeb4 Eif5 Rps26 Cnbp Eif4ebp1 Rps21 Rpl18a Rpl29 Rpl38 Rpl32 Rpl36 Rpl26 Rpl31 Rpl24
GO:Biological Process	2.69E-09	10	37	25.54204	Porphyrin-containing compound metabolic process	Hmox1 Slc25a39 Cpox Fech Alas2 Urod Ank1 Hmbs Atpif1 Ppox
GO:Biological Process	2.69E-09	9	26	31.82931	Porphyrin-containing compound biosynthetic process	Slc25a39 Cpox Fech Alas2 Urod Ank1 Hmbs Atpif1 Ppox
GO:Biological Process	2.69E-09	17	176	9.141362	Myeloid cell homeostasis	Hmox1 Slc4a1 Epb42 Rhag Tspo2 Rps14 Alas2 Fam210b Tal1 Ank1 Ncapg2 Sox6 Klf1 Atpif1 Hmgb2 Hba-a1 Trim10
GO:Biological Process	6.42E-09	9	29	28.53663	Tetrapyrrole biosynthetic process	Slc25a39 Cpox Fech Alas2 Urod Ank1 Hmbs Atpif1 Ppox
GO:Biological Process	1.11E-08	9	31	26.69555	Heme metabolic process	Hmox1 Slc25a39 Cpox Fech Alas2 Urod Hmbs Atpif1 Ppox
GO:Biological Process	1.12E-08	10	46	20.89803	Tetrapyrrole metabolic process	Hmox1 Slc25a39 Cpox Fech Alas2 Urod Ank1 Hmbs Atpif1 Ppox
GO:Biological Process	1.46E-08	8	22	33.43686	Heme biosynthetic process	Slc25a39 Cpox Fech Alas2 Urod Hmbs Atpif1 Ppox
GO:Biological Process	6.12E-08	9	39	21.77795	Erythrocyte development	Slc4a1 Epb42 Rhag Alas2 Fam210b Tal1 Ank1 Sox6 Hba-a1
GO:Biological Process	1.74E-07	6	11	50.15528	Protoporphyrinogen IX metabolic process	Cpox Fech Alas2 Urod Hmbs Ppox
GO:Biological Process	3.19E-07	19	322	5.617607	Homeostasis of number of cells	Prdx2 Hmox1 Slc4a1 Epb42 Rhag Tspo2 Rps14 Alas2 Fam210b Tal1 Ank1 Ccnb2 Ncapg2 Sox6 Klf1 Atpif1 Hmgb2 Hba-a1 Trim10
GO:Biological Process	1.00E-06	16	240	6.396616	Response to toxic substance	Cd36 Prdx2 Prxl2a Cpox Pim1 Fech Mgst3 Cat Wapl Ccnb1 Mbp Hbb-bs Gpx1 Hba-a1 Hbb-bt Gpx4
GO:Biological Process	1.08E-06	20	402	4.891029	Myeloid cell differentiation	Slc4a1 Hmgb3 Prxl2a Tfrc Epb42 Rhag Tspo2 Rps14 Alas2 Fam210b Car2 Tal1 Ank1 Ncapg2 Sox6 Klf1 Atpif1 Hmgb2 Hba-a1 Trim10
GO:Biological Process	1.72E-06	41	1552	2.609	Organonitrogen compound biosynthetic process	Hnrnpd Rps18 Rps15a Pnpo Slc25a39 Acsl1 Cpeb4 Eif5 Cpox Fech Rps14 Alas2 Rps26 Ptdss2 Snca Trak2 Gclm Urod Cnbp Rbm3 Eif4ebp1 Ank1 Hmbs Gclc Rpl12 Rps21 Rpl18a Rpl29 Atpif1 St3gal5 Rpl38 Rpl32 Rpl36 Rpl23a Rpl26 Rps7 Ppox Rpl35 Mocs1 Rpl31 Rpl24
GO:Biological Process	2.06E-06	25	660	3.774686	Peptide biosynthetic process	Hnrnpd Rps18 Rps15a Cpeb4 Eif5 Rps14 Rps26 Gclm Cnbp Rbm3 Eif4ebp1 Gclc Rpl12 Rps21 Rpl18a Rpl29 Rpl38 Rpl32 Rpl36 Rpl23a Rpl26 Rps7 Rpl35 Rpl31 Rpl24

GO:Biological Process	2.18E-06	11	108	10.0145	Cellular detoxification	Cd36 Prdx2 Prxl2a Pim1 Mgst3 Cat Hbb-bs Gpx1 Hba-a1 Hbb-bt Gpx4
GO:Biological Process	2.71E-06	5	9	51.08408	Protoporphyrinogen IX biosynthetic process	Cpox Alas2 Urod Hmbs Ppox
GO:Biological Process	2.71E-06	27	776	3.462603	Amide biosynthetic process	Hnrnpd Rps18 Rps15a Acsl1 Cpeb4 Eif5 Rps14 Rps26 Snca Gclm Cnbp Rbm3 Eif4ebp1 Gclc Rpl12 Rps21 Rpl18a Rpl29 Rpl38 Rpl32 Rpl36 Rpl23a Rpl26 Rps7 Rpl35 Rpl31 Rpl24
GO:Biological Process	3.80E-06	11	115	9.365415	Cellular response to toxic substance	Cd36 Prdx2 Prxl2a Pim1 Mgst3 Cat Hbb-bs Gpx1 Hba-a1 Hbb-bt Gpx4
GO:Biological Process	4.66E-06	9	68	12.73173	Pigment metabolic process	Hmox1 Slc25a39 Cpox Fech Alas2 Urod Hmbs Atpif1 Ppox
GO:Biological Process	4.71E-06	23	578	3.831306	Cell division	Ube2c Spag5 Ccne1 Kif11 Cdc6 Cdk1 Cdca3 Spire1 Cat Nusap1 Cdc25b Tpx2 Smc2 Tal1 Epb41 Cenpa Blm Ccnb2 Kif4 Ncaph Wapl Ccnb1 Ncapg2
GO:Biological Process	5.43E-06	10	94	10.33161	Cellular oxidant detoxification	Cd36 Prdx2 Prxl2a Mgst3 Cat Hbb-bs Gpx1 Hba-a1 Hbb-bt Gpx4
GO:Biological Process	5.43E-06	7	43	20.11436	Chromosome condensation	Cdk1 Nusap1 Smc2 Ncaph Wapl Ccnb1 Ncapg2
GO:Biological Process	5.93E-06	11	123	8.795347	Detoxification	Cd36 Prdx2 Prxl2a Pim1 Mgst3 Cat Hbb-bs Gpx1 Hba-a1 Hbb-bt Gpx4
GO:Biological Process	6.86E-06	27	818	3.258119	Peptide metabolic process	Hnrnpd Rps18 Rps15a Cpeb4 Eif5 Rps14 Rps26 Gclm Cnbp Rbm3 Eif4ebp1 Gclc Rpl12 Rps21 Rpl18a Rpl29 Rpl38 Rpl32 Rpl36 Rpl23a Rpl26 Rps7 Rpl35 Gpx1 Rpl31 Gpx4 Rpl24
GO:Biological Process	7.45E-06	6	22	26.27181	Gas transport	Aqp1 Rhag Car2 Hbb-bs Hba-a1 Hbb-bt
GO:Biological Process	7.45E-06	6	22	26.27181	Hydrogen peroxide catabolic process	Prdx2 Snca Cat Hbb-bs Gpx1 Hbb-bt
GO:Biological Process	7.45E-06	8	54	14.42374	Pigment biosynthetic process	Slc25a39 Cpox Fech Alas2 Urod Hmbs Atpif1 Ppox
GO:Biological Process	1.13E-05	23	640	3.59063	Translation	Hnrnpd Rps18 Rps15a Cpeb4 Eif5 Rps14 Rps26 Cnbp Rbm3 Eif4ebp1 Rpl12 Rps21 Rpl18a Rpl29 Rpl38 Rpl32 Rpl36 Rpl23a Rpl26 Rps7 Rpl35 Rpl31 Rpl24
GO:Biological Process	1.45E-05	9	79	10.74756	Myeloid cell development	Slc4a1 Epb42 Rhag Alas2 Fam210b Tal1 Ank1 Sox6 Hba-a1
GO:Biological Process	2.91E-05	22	601	3.518139	Response to inorganic substance	Hnrnpd Cd36 Aqp1 Prdx2 Hmox1 Epor Slc4a1 Net1 Cpox Tfrc Bsg Fech Snca Cat Car2 Vcam1 Blm Gclc Ccnb1 Fabp4 Gpx1 Slfn14
GO:Biological Process	6.02E-05	23	663	3.248665	Mitotic cell cycle process	Ube2c Spag5 Ccne1 Kif11 Cdc6 Cdk1 Nusap1 Cdc25b Tpx2 E2f1 Smc2 Cenpa Blm Mki67 Eif4ebp1 Ccnb2 Kif4 Ncaph Ccnb1 Ncapg2 Rpl26 Psme3 Rpl24
GO:Biological Process	7.41E-05	29	1060	2.718236	Cellular amide metabolic process	Hnrnpd Rps18 Rps15a Acsl1 Cpeb4 Eif5 Rps14 Rps26 Snca Gclm Cnbp Rbm3 Eif4ebp1 Gclc Rpl12 Rps21 Rpl18a Rpl29 Rpl38 Rpl32 Rpl36 Rpl23a Rpl26 Rps7 Rpl35 Gpx1 Rpl31 Gpx4 Rpl24
GO:Biological Process	0.000138	25	810	2.906174	Mitotic cell cycle	Ube2c Spag5 Ccne1 Kif11 Cdc6 Cdk1 Nusap1 Cdc25b Tpx2 E2f1 Smc2 Tal1 Cenpa Blm Mki67 Eif4ebp1 Ccnb2 Kif4 Ncaph Wapl Ccnb1 Ncapg2 Rpl26 Psme3 Rpl24
GO:Biological Process	0.000147	17	426	3.917727	Nuclear division	Ube2c Spag5 Ccne1 Kif11 Cdc6 Spire1 Nusap1 Cdc25b Tpx2 Smc2 Mki67 Ccnb2 Kif4 Ncaph Ccnb1 Ncapg2 Rpl24

GO:Biological Process	0.000451	13	270	4.597568	Mitotic nuclear division	Ube2c Spag5 Kif11 Cdc6 Nusap1 Tpx2 Smc2 Mki67 Kif4 Ncaph Ccnb1 Ncapg2 Rpl24
GO:Biological Process	0.000472	25	889	2.698103	Regulation of cell cycle	Ube2c Spag5 Ccne1 Rps15a Kif11 Cdc6 E2f2 Cdk1 Ccndbp1 Pim1 Btrc Nusap1 Cdc25b Tpx2 E2f1 Tal1 Blm Mki67 Eif4ebp1 Ccnb2 Tfdp2 Wapl Ccnb1 Rpl26 Psme3
GO:Biological Process	0.000585	17	475	3.512748	Organelle fission	Ube2c Spag5 Ccne1 Kif11 Cdc6 Spire1 Nusap1 Cdc25b Tpx2 Smc2 Mki67 Ccnb2 Kif4 Ncaph Ccnb1 Ncapg2 Rpl24
GO:Biological Process	0.000617	38	1787	2.094815	Homeostatic process	Ube2c Cd36 Clcn3 Aqp1 Prdx2 Hmox1 Epor Slc4a1 Acsl1 Tfrc Bsg Epb42 Rhag Tspo2 Rps14 Alas2 Snca Fam210b Car2 Vcam1 Gclm Tal1 Prkab1 Ank1 Ccnb2 Gclc Mbp Ncapg2 Abcb4 Sox6 Klf1 Atpif1 Hmgb2 Ncoa4 Fabp4 Gpx1 Hba-a1 Trim10
GO:Biological Process	0.000718	6	51	11.99365	Hydrogen peroxide metabolic process	Prdx2 Snca Cat Hbb-bs Gpx1 Hbb-bt
GO:Biological Process	0.000718	25	948	2.618205	Hematopoietic or lymphoid organ development	Prdx2 Slc4a1 Hmgb3 Prxl2a Tfrc Epb42 Rhag Tspo2 Pim1 Rps14 Alas2 Fam210b Car2 Tal1 Add2 Blm Ank1 Ccnb2 Ncapg2 Sox6 Klf1 Atpif1 Hmgb2 Hba-a1 Trim10
GO:Biological Process	0.000921	24	904	2.642913	Hemopoiesis	Prdx2 Slc4a1 Hmgb3 Prxl2a Tfrc Epb42 Rhag Tspo2 Pim1 Rps14 Alas2 Fam210b Car2 Tal1 Add2 Blm Ank1 Ncapg2 Sox6 Klf1 Atpif1 Hmgb2 Hba-a1 Trim10
GO:Biological Process	0.00094	15	388	3.71771	Response to metal ion	Hnrnpd Aqp1 Hmox1 Cpox Tfrc Bsg Fech Snca Cat Car2 Vcam1 Gclc Ccnb1 Fabp4 Slfn14
GO:Biological Process	0.00166	34	1648	2.103867	Cell cycle	Ube2c Spag5 Ccne1 Rps15a Kif11 Cdc6 E2f2 Cdk1 Cdca3 Ccndbp1 Pim1 Spire1 Btrc Nusap1 Cdc25b Tpx2 E2f1 Smc2 Tal1 Epb41 Cenpa Blm Mki67 Eif4ebp1 Ccnb2 Tfdp2 Kif4 Ncaph Wapl Ccnb1 Ncapg2 Rpl26 Psme3 Rpl24
GO:Biological Process	0.001681	6	63	10.21682	Mitochondrial ATP synthesis coupled electron transport	Cdk1 Ndufb9 Ndufb8 Snca Ccnb1 Ndufs8
GO:Biological Process	0.001683	7	89	8.045743	Iron ion homeostasis	Hmox1 Tfrc Epb42 Rhag Alas2 Ank1 Ncoa4
GO:Biological Process	0.001723	10	185	5.108408	Sister chromatid segregation	Ube2c Spag5 Cdc6 Nusap1 Smc2 Kif4 Ncaph Wapl Ccnb1 Ncapg2
GO:Biological Process	0.001723	6	66	10.03106	ATP synthesis coupled electron transport	Cdk1 Ndufb9 Ndufb8 Snca Ccnb1 Ndufs8
GO:Biological Process	0.001723	25	1009	2.461225	Immune system development	Prdx2 Slc4a1 Hmgb3 Prxl2a Tfrc Epb42 Rhag Tspo2 Pim1 Rps14 Alas2 Fam210b Car2 Tal1 Add2 Blm Ank1 Ccnb2 Ncapg2 Sox6 Klf1 Atpif1 Hmgb2 Hba-a1 Trim10
GO:Biological Process	0.001847	5	36	13.52226	Response to iron ion	Hmox1 Cpox Tfrc Snca Ccnb1
GO:Biological Process	0.002095	5	35	13.13591	Response to arsenic-containing substance	Hmox1 Slc4a1 Cpox Fech Gclc
GO:Biological Process	0.002431	9	157	5.444488	Mitotic sister chromatid segregation	Ube2c Spag5 Cdc6 Nusap1 Smc2 Kif4 Ncaph Ccnb1 Ncapg2
GO:Biological Process	0.002431	3	8	39.40772	Carbon dioxide transport	Aqp1 Rhag Car2
GO:Biological Process	0.002458	10	262	4.839545	DNA conformation change	Hmgb3 Cdk1 Nusap1 Smc2 Blm Ncaph Wapl Ccnb1 Ncapg2 Hmgb2
GO:Biological Process	0.003161	12	288	3.940772	Ribosome biogenesis	Riok3 Rps14 Nop56 Rps21 Isg20 Rps10 Rpl38 Rpl23a Rpl26 Rps7 Rpl35 Rpl24
GO:Biological Process	0.00369	7	96	6.921069	Negative regulation of extrinsic apoptotic signaling pathway	Prdx2 Hmox1 Gclm Gclc Hmgb2 Gpx1 Psme3

GO:Biological Process	0.004603	6	69	8.234449	Ribosomal small subunit biogenesis	Riok3 Rps14 Rps21 Rps10 Rpl38 Rps7
GO:Biological Process	0.005562	11	270	3.982145	Nuclear chromosome segregation	Ube2c Spag5 Ccne1 Cdc6 Nusap1 Smc2 Kif4 Ncaph Wapl Ccnb1 Ncapg2
GO:Biological Process	0.005562	7	118	6.436595	Oxidative phosphorylation	Cox6c Cdk1 Ndufb9 Ndufb8 Snca Ccnb1 Ndufs8
GO:Biological Process	0.006393	7	175	6.249121	DNA packaging	Cdk1 Nusap1 Smc2 Ncaph Wapl Ccnb1 Ncapg2
GO:Biological Process	0.006393	25	1079	2.238348	Cell cycle process	Ube2c Spag5 Ccne1 Kif11 Cdc6 Cdk1 Spire1 Nusap1 Cdc25b Tpx2 E2f1 Smc2 Cenpa Blm Mki67 Eif4ebp1 Ccnb2 Kif4 Ncaph Wapl Ccnb1 Ncapg2 Rpl26 Psme3 Rpl24
GO:Biological Process	0.006393	14	413	3.202286	Ribonucleoprotein complex biogenesis	Luc7l3 Eif5 Riok3 Rps14 Nop56 Rps21 Isg20 Rps10 Rpl38 Rpl23a Rpl26 Rps7 Rpl35 Rpl24
GO:Biological Process	0.007663	21	897	2.422809	Chromosome organization	Hnrnpd Ube2c Spag5 Ccne1 Hmgb3 Cdc6 Cdk1 Dek Nusap1 Smc2 Tal1 Cenpa Blm Mki67 Kif4 Ncaph Wapl Ccnb1 Ncapg2 Hmgb2 Gpx4
GO:Biological Process	0.007703	12	329	3.525291	Chromosome segregation	Ube2c Spag5 Ccne1 Cdc6 Nusap1 Smc2 Mki67 Kif4 Ncaph Wapl Ccnb1 Ncapg2
GO:Biological Process	0.007703	6	87	7.356108	Respiratory electron transport chain	Cdk1 Ndufb9 Ndufb8 Snca Ccnb1 Ndufs8
GO:Biological Process	0.008419	13	370	3.27498	Mitotic cell cycle phase transition	Ube2c Ccne1 Cdc6 Cdk1 Cdc25b E2f1 Blm Eif4ebp1 Ccnb2 Ccnb1 Rpl26 Psme3 Rpl24
GO:Biological Process	0.00845	8	171	5.073178	Aerobic respiration	Cox6c Cdk1 Ndufb9 Ndufb8 Snca Cat Ccnb1 Ndufs8
GO:Biological Process	0.008653	3	12	25.07764	Cellular response to iron ion	Hmox1 Tfrc Ccnb1
GO:Biological Process	0.009755	14	442	3.043307	Response to oxidative stress	Cd36 Aqp1 Prdx2 Hmox1 Epor Slc4a1 Net1 Snca Cat Gclm Gclc Ndufs8 Gpx1 Gpx4
GO:Biological Process	0.011569	6	84	6.728148	Regulation of cyclin-dependent protein serine/threonine kinase activity	Ccne1 Cdc6 Pim1 Blm Ccnb2 Ccnb1
GO:Biological Process	0.012995	7	138	5.454741	Electron transport chain	Cox6c Cdk1 Ndufb9 Ndufb8 Snca Ccnb1 Ndufs8
GO:Biological Process	0.013594	3	13	21.21954	Mitotic chromosome condensation	Nusap1 Smc2 Ncaph
GO:Biological Process	0.013594	3	13	21.21954	Response to insecticide	Cpox Fech Ccnb1
GO:Biological Process	0.013594	3	14	21.21954	One-carbon compound transport	Aqp1 Rhag Car2
GO:Biological Process	0.013894	6	88	6.415211	Regulation of cyclin-dependent protein kinase activity	Ccne1 Cdc6 Pim1 Blm Ccnb2 Ccnb1
GO:Biological Process	0.013894	2	3	61.3009	Positive regulation of mitochondrial ATP synthesis coupled electron transport	Cdk1 Ccnb1
GO:Biological Process	0.015458	7	136	5.233004	Transition metal ion homeostasis	Hmox1 Tfrc Epb42 Rhag Alas2 Ank1 Ncoa4
GO:Biological Process	0.015713	11	313	3.417111	Positive regulation of cell cycle	Ube2c Spag5 Rps15a Cdc6 Cdk1 Pim1 Nusap1 Cdc25b Tal1 Eif4ebp1 Ccnb1
GO:Biological Process	0.015811	5	63	7.926841	Ribosome assembly	Rps14 Rps10 Rpl38 Rpl23a Rpl24
GO:Biological Process	0.015907	3	14	19.70386	Oxygen transport	Hbb-bs Hba-a1 Hbb-bt
GO:Biological Process	0.016636	9	222	3.997885	Reactive oxygen species metabolic process	Cd36 Clcn3 Prdx2 Snca Cat Hbb-bs Atpif1 Gpx1 Hbb-bt
GO:Biological Process	0.016974	12	361	3.152618	Non-membrane-bounded organelle assembly	Spag5 Kif11 Rps14 Tpx2 Tmod1 Cenpa Ccnb2 Kif4 Rps10 Rpl38 Rpl23a Rpl24
GO:Biological Process	0.018254	13	416	2.951525	Regulation of mitotic cell cycle	Ube2c Cdc6 Cdk1 Nusap1 Cdc25b E2f1 Tal1 Blm Mki67 Eif4ebp1 Ccnb1 Rpl26 Psme3
GO:Biological Process	0.018617	3	16	18.39027	Erythrocyte maturation	Epb42 Fam210b Tal1
GO:Biological Process	0.018617	5	63	7.536996	Positive regulation of fibroblast proliferation	Aqp1 Cdc6 E2f1 Wapl Ccnb1

GO:Biological Process	0.020837	28	1457	1.927124	Protein-containing complex assembly	Ube2c Tspan33 Cd36 Cdk1 Luc7l3 Eif5 Ndufb9 Tfrc Riok3 Spire1 Rps14 Ndufb8 Snca Tpx2 Tmod1 Tal1 Epb41 Cenpa Add2 Blm Eif4ebp1 Ccnb1 Rps10 Rpl38 Rpl23a Ndufs8 Gpx4 Rpl24
GO:Biological Process	0.021117	4	38	10.21682	Female meiotic nuclear division	Spire1 Cdc25b Ccnb2 Ncaph
GO:Biological Process	0.021563	9	224	3.813651	Response to reactive oxygen species	Cd36 Aqp1 Prdx2 Hmox1 Epor Slc4a1 Net1 Cat Gpx1
GO:Biological Process	0.021702	3	17	17.24088	Hemoglobin metabolic process	Epb42 Alas2 Cat
GO:Biological Process	0.021934	2	4	45.97568	Response to thyroxine	Gclm Gclc
GO:Biological Process	0.021934	2	4	45.97568	L-alanine transmembrane transport	Slc3a2 Slc38a5
GO:Biological Process	0.021934	2	4	45.97568	Regulation of mitochondrial ATP synthesis coupled electron transport	Cdk1 Ccnb1
GO:Biological Process	0.021934	2	4	45.97568	Lens fiber cell apoptotic process	E2f2 E2f1
GO:Biological Process	0.021934	22	1043	2.127161	Response to organic cyclic compound	Hnrnpd Cd36 Aqp1 Slc3a2 Acsl1 Bsg Pim1 Riok3 Fech Btrc Snca Cat Fam210b Car2 Gclm Blm Eif4ebp1 Gclc Ccnb1 Abcb4 Hmgb2 Gpx4
GO:Biological Process	0.021934	5	69	7.073181	Ribosomal large subunit biogenesis	Rpl38 Rpl23a Rpl26 Rpl35 Rpl24
GO:Biological Process	0.021934	2	4	45.97568	Response to human chorionic gonadotropin	Gclm Gclc
GO:Biological Process	0.021934	5	69	7.073181	Response to fatty acid	Cd36 Acsl1 Cat E2f1 Ccnb1
GO:Biological Process	0.022112	9	228	3.744625	Cellular response to inorganic substance	Hnrnpd Aqp1 Hmox1 Tfrc Snca Blm Ccnb1 Fabp4 Slfn14
GO:Biological Process	0.02764	32	1798	1.78114	Response to oxygen-containing compound	Hnrnpd Cd36 Aqp1 Prdx2 Hmox1 Epor Slc4a1 Cdc6 Acsl1 Cdk1 Cpeb4 Net1 Bsg Pim1 Fech Snca Cat E2f1 Fam210b Car2 Vcam1 Gclm Blm Eif4ebp1 Gclc Ccnb1 Abcb4 Klf1 Hmgb2 Gpx1 Gpx4 Ly6c1
GO:Biological Process	0.027764	3	20	15.32523	Ribosomal small subunit assembly	Rps14 Rps10 Rpl38
GO:Biological Process	0.030267	9	261	3.567078	ATP metabolic process	Slc4a1 Cox6c Cdk1 Ndufb9 Ndufb8 Snca Ccnb1 Atpif1 Ndufs8
GO:Biological Process	0.030516	8	215	3.933748	Cellular respiration	Cox6c Cdk1 Ndufb9 Ndufb8 Snca Cat Ccnb1 Ndufs8
GO:Biological Process	0.032081	2	5	36.78054	Leucine import across plasma membrane	Slc3a2 Slc43a1
GO:Biological Process	0.032081	2	5	36.78054	L-leucine import across plasma membrane	Slc3a2 Slc43a1
GO:Biological Process	0.032081	2	5	36.78054	Response to L-phenylalanine derivative	Gclm Gclc
GO:Biological Process	0.032081	2	5	36.78054	G2/MI transition of meiotic cell cycle	Cdc25b Ccnb2
GO:Biological Process	0.032081	13	452	2.704452	Cell cycle phase transition	Ube2c Ccne1 Cdc6 Cdk1 Cdc25b E2f1 Blm Eif4ebp1 Ccnb2 Ccnb1 Rpl26 Psme3 Rpl24
GO:Biological Process	0.032081	2	5	36.78054	Regulation of chromosome condensation	Wapl Ccnb1
GO:Biological Process	0.032081	2	6	36.78054	Visceral serous pericardium development	Epor Vcam1
GO:Biological Process	0.035946	24	1229	1.947778	Cellular response to oxygen-containing compound	Hnrnpd Cd36 Aqp1 Prdx2 Epor Cdc6 Cpeb4 Net1 Pim1 Fech E2f1 Fam210b Car2 Vcam1 Gclm Blm Eif4ebp1 Gclc Ccnb1 Abcb4 Klf1 Hmgb2 Gpx1 Ly6c1
GO:Biological Process	0.036406	7	154	4.262645	Cellular response to decreased oxygen levels	Aqp1 Hmox1 Cpeb4 Bnip3l E2f1 Eif4ebp1 Ccnb1
GO:Biological Process	0.036461	6	114	4.970343	Positive regulation of mitotic cell cycle	Ube2c Cdk1 Cdc25b Tal1 Eif4ebp1 Ccnb1
GO:Biological Process	0.036991	15	590	2.432575	Apoptotic signaling pathway	Prdx2 Hmox1 E2f2 Bnip3l E2f1 Gclm Ybx3 Gclc Bclaf1 Atpif1 Hmgb2 Rpl26 Rps7 Gpx1 Psme3

GO:Biological Process	0.039382	7	158	4.179607	Regulation of extrinsic apoptotic signaling pathway	Prdx2 Hmox1 Gclm Gclc Hmgb2 Gpx1 Psme3
GO:Biological Process	0.039382	7	158	4.179607	Response to hydrogen peroxide	Aqp1 Hmox1 Epor Slc4a1 Net1 Cat Gpx1
GO:Biological Process	0.043695	3	28	12.53882	Mitochondrial electron transport, NADH to ubiquinone	Ndufb9 Ndufb8 Ndufs8
GO:Biological Process	0.043695	15	596	2.382159	Regulation of cell cycle process	Ube2c Spag5 Kif11 Cdc6 Cdk1 Nusap1 Cdc25b Tpx2 E2f1 Blm Mki67 Wapl Ccnb1 Rpl26 Psme3
GO:Biological Process	0.043695	5	83	5.819706	Regulation of chromosome segregation	Ube2c Cdc6 Mki67 Wapl Ccnb1
GO:Biological Process	0.043828	2	6	30.65045	Meiotic chromosome condensation	Smc2 Ncaph
GO:Biological Process	0.047799	9	257	3.258119	Regulation of mitotic cell cycle phase transition	Ube2c Cdc6 Cdk1 Cdc25b E2f1 Blm Ccnb1 Rpl26 Psme3
GO:Biological Process	0.048013	3	24	11.99365	Mitochondrial depolarization	Gclm Gclc Atpif1
GO:Molecular Function	3.26E-11	18	172	10.88898	Structural constituent of ribosome	Rps18 Rps15a Rps14 Rps26 Rpl12 Rps21 Rpl18a Rpl29 Rps10 Rpl38 Rpl32 Rpl36 Rpl23a Rpl26 Rps7 Rpl35 Rpl31 Rpl24
GO:Molecular Function	2.13E-08	26	612	4.624246	Structural molecule activity	Hmox1 Rps18 Rps15a Cldn13 Cpox Rps14 Rps26 Tinagl1 Epb41 Add2 Rpl12 Rps21 Mbp Isca1 Rpl18a Rpl29 Rps10 Rpl38 Rpl32 Rpl36 Rpl23a Rpl26 Rps7 Rpl35 Rpl31 Rpl24
GO:Molecular Function	1.17E-05	8	53	14.71222	Peroxidase activity	Prdx2 Mgst3 Cat Hbb-bs Gpx1 Hba-a1 Hbb-bt Gpx4
GO:Molecular Function	1.63E-05	8	57	13.62242	Oxidoreductase activity, acting on peroxide as acceptor	Prdx2 Mgst3 Cat Hbb-bs Gpx1 Hba-a1 Hbb-bt Gpx4
GO:Molecular Function	2.40E-05	9	85	10.34453	Antioxidant activity	Prdx2 Prxl2a Mgst3 Cat Hbb-bs Gpx1 Hba-a1 Hbb-bt Gpx4
GO:Molecular Function	0.004206	12	291	4.056677	MRNA binding	Hnrnpd Carhsp1 Cpeb4 Luc7l3 Rps14 Rps26 Cnbp Ybx3 Pcf11 Rpl26 Rps7 Rpl35
GO:Molecular Function	0.007256	3	9	30.65045	Hemoglobin binding	Slc4a1 Hbb-bs Hbb-bt
GO:Molecular Function	0.007256	3	9	30.65045	Haptoglobin binding	Hbb-bs Hba-a1 Hbb-bt
GO:Molecular Function	0.011757	5	51	9.014838	Hsp70 protein binding	Dnajb1 Cdk1 Tfrc Snca Dnajb2
GO:Molecular Function	0.011757	19	814	2.524676	Oxidoreductase activity	Prdx2 Hmox1 Cox6c Pnpo Cpox Ndufb8 Snca Acadl Mgst3 Cat Vcam1 Gclm Hbb-bs Ndufs8 Ppox Gpx1 Hba-a1 Hbb-bt Gpx4
GO:Molecular Function	0.011757	25	1163	2.170712	RNA binding	Hnrnpd Carhsp1 Rps18 Slc3a2 Hmgb3 Cpeb4 Luc7l3 Eif5 Tfrc Rps14 Rps26 Nop56 Cnbp Ybx3 Rbm3 Rpl12 Isg20 Pcf11 Tent5c Rps10 Rpl23a Rpl26 Rps7 Rpl35 Rpl24
GO:Molecular Function	0.01595	3	13	21.21954	Oxygen carrier activity	Hbb-bs Hba-a1 Hbb-bt
GO:Molecular Function	0.030819	5	71	6.966011	Iron-sulfur cluster binding	Fech Kif4 Isca1 Ndufs8 Mocs1
GO:Molecular Function	0.030819	5	71	6.966011	Metal cluster binding	Fech Kif4 Isca1 Ndufs8 Mocs1
GO:Molecular Function	0.041203	3	20	14.51863	Glutathione peroxidase activity	Mgst3 Gpx1 Gpx4
GO:Molecular Function	0.041231	2	5	36.78054	Hemoglobin alpha binding	Hbb-bs Hbb-bt
GO:Molecular Function	0.046572	7	158	4.234602	Ribonucleoprotein complex binding	Cpeb4 Pim1 Ybx3 Rbm3 Rps21 Rpl35 Slfn14
KEGG	2.97E-13	18	130	13.13591	Ribosome	Rps18 Rps15a Rps14 Rps26 Rpl12 Rps21 Rpl18a Rpl29 Rps10 Rpl38 Rpl32 Rpl36 Rpl23a Rpl26 Rps7 Rpl35 Rpl31 Rpl24
KEGG	9.55E-10	18	231	7.957328	Coronavirus disease	Rps18 Rps15a Rps14 Rps26 Rpl12 Rps21 Rpl18a Rpl29 Rps10 Rpl38 Rpl32 Rpl36 Rpl23a Rpl26 Rps7 Rpl35 Rpl31 Rpl24

KEGG	2.48E-07	8	40	19.88137	Ferroptosis	Hmox1 Slc3a2 Acsl1 Tfrc Gclm Gclc Ncoa4 Gpx4
KEGG	7.68E-07	7	43	22.19515	Porphyrin and chlorophyll metabolism	Hmox1 Cpox Fech Alas2 Urod Hmbs Ppox
KEGG	7.08E-05	10	154	6.761129	Biosynthesis of cofactors	Pnpo Cpox Fech Alas2 Gclm Urod Hmbs Gclc Ppox Mocs1
KEGG	0.000186	9	125	6.783296	Cell cycle	Ccne1 Cdc6 E2f2 Cdk1 Cdc25b E2f1 Ccnb2 Tfdp2 Ccnb1
KEGG	0.000491	6	55	10.40959	Malaria	Cd36 Vcam1 Gypa Hbb-bs Hba-a1 Hbb-bt
KEGG	0.007495	8	174	4.540807	Cellular senescence	Ccne1 E2f2 Cdk1 Btrc E2f1 Eif4ebp1 Ccnb2 Ccnb1
KEGG	0.01255	5	72	6.966011	Glutathione metabolism	Mgst3 Gclm Gclc Gpx1 Gpx4
KEGG	0.019239	6	120	5.015528	Oocyte meiosis	Ccne1 Cdk1 Cpeb4 Btrc Ccnb2 Ccnb1
KEGG	0.020812	8	220	3.696537	Chemical carcinogenesis	Hmox1 Cox6c Ndufb9 Ndufb8 Mgst3 Cat Acp1 Ndufs8
KEGG	0.029272	5	92	5.408903	Progesterone-mediated oocyte maturation	Cdk1 Cpeb4 Cdc25b Ccnb2 Ccnb1
KEGG	0.046553	27	1597	1.713379	Metabolic pathways	Hmox1 Cox6c Pnpo Acsl1 Ndufb9 Cpox Fech Ndufb8 Alas2 Ptdss2 Acadl Mgst3 Cat Car1 Car2 Gclm Urod Nt5c3 Hmbs Gclc Acp1 St3gal5 Ndufs8 Ppox Gpx1 Mocs1 Gpx4
Reactome	1.19E-16	20	122	16.27458	Eukaryotic Translation Initiation	Rps18 Rps15a Eif5 Rps14 Rps26 Eif4ebp1 Rpl12 Rps21 Rpl18a Rpl29 Rps10 Rpl38 Rpl32 Rpl36 Rpl23a Rpl26 Rps7 Rpl35 Rpl31 Rpl24
Reactome	1.19E-16	20	122	16.27458	Cap-dependent Translation Initiation	Rps18 Rps15a Eif5 Rps14 Rps26 Eif4ebp1 Rpl12 Rps21 Rpl18a Rpl29 Rps10 Rpl38 Rpl32 Rpl36 Rpl23a Rpl26 Rps7 Rpl35 Rpl31 Rpl24
Reactome	2.33E-16	18	96	19.02442	SRP-dependent cotranslational protein targeting to membrane	Rps18 Rps15a Rps14 Rps26 Rpl12 Rps21 Rpl18a Rpl29 Rps10 Rpl38 Rpl32 Rpl36 Rpl23a Rpl26 Rps7 Rpl35 Rpl31 Rpl24
Reactome	2.33E-16	18	97	18.80823	Nonsense Mediated Decay NMD independent of the Exon Junction Complex EJC	Rps18 Rps15a Rps14 Rps26 Rpl12 Rps21 Rpl18a Rpl29 Rps10 Rpl38 Rpl32 Rpl36 Rpl23a Rpl26 Rps7 Rpl35 Rpl31 Rpl24
Reactome	2.90E-16	19	115	16.48185	GTP hydrolysis and joining of the 60S ribosomal subunit	Rps18 Rps15a Eif5 Rps14 Rps26 Rpl12 Rps21 Rpl18a Rpl29 Rps10 Rpl38 Rpl32 Rpl36 Rpl23a Rpl26 Rps7 Rpl35 Rpl31 Rpl24
Reactome	6.69E-16	18	104	17.42236	Formation of a pool of free 40S subunits	Rps18 Rps15a Rps14 Rps26 Rpl12 Rps21 Rpl18a Rpl29 Rps10 Rpl38 Rpl32 Rpl36 Rpl23a Rpl26 Rps7 Rpl35 Rpl31 Rpl24
Reactome	3.76E-15	18	114	15.76309	L13a-mediated translational silencing of Ceruloplasmin expression	Rps18 Rps15a Rps14 Rps26 Rpl12 Rps21 Rpl18a Rpl29 Rps10 Rpl38 Rpl32 Rpl36 Rpl23a Rpl26 Rps7 Rpl35 Rpl31 Rpl24
Reactome	4.16E-15	18	118	15.46845	Nonsense-Mediated Decay NMD	Rps18 Rps15a Rps14 Rps26 Rpl12 Rps21 Rpl18a Rpl29 Rps10 Rpl38 Rpl32 Rpl36 Rpl23a Rpl26 Rps7 Rpl35 Rpl31 Rpl24
Reactome	7.67E-14	20	181	10.81781	Major pathway of rRNA processing in the nucleolus and cytosol	Rps18 Rps15a Riok3 Rps14 Rps26 Nop56 Rpl12 Rps21 Rpl18a Rpl29 Rps10 Rpl38 Rpl32 Rpl36 Rpl23a Rpl26 Rps7 Rpl35 Rpl31 Rpl24
Reactome	7.67E-14	20	181	10.81781	RRNA processing	Rps18 Rps15a Riok3 Rps14 Rps26 Nop56 Rpl12 Rps21 Rpl18a Rpl29 Rps10 Rpl38 Rpl32 Rpl36 Rpl23a Rpl26 Rps7 Rpl35 Rpl31 Rpl24

Reactome	7.67E-14	20	181	10.81781	RRNA processing in the nucleus and cytosol	Rps18 Rps15a Riok3 Rps14 Rps26 Nop56 Rpl12 Rps21 Rpl18a Rpl29 Rps10 Rpl38 Rpl32 Rpl36 Rpl23a Rpl26 Rps7 Rpl35 Rpl31 Rpl24
Reactome	5.64E-12	8	13	56.58545	Erythrocytes take up carbon dioxide and release oxygen	Aqp1 Slc4a1 Rhag Car1 Car2 Hbb-bs Hba-a1 Hbb-bt
Reactome	5.64E-12	8	13	56.58545	O2/CO2 exchange in erythrocytes	Aqp1 Slc4a1 Rhag Car1 Car2 Hbb-bs Hba-a1 Hbb-bt
Reactome	1.04E-11	20	232	8.283906	Translation	Rps18 Rps15a Eif5 Rps14 Rps26 Eif4ebp1 Rpl12 Rps21 Rpl18a Rpl29 Rps10 Rpl38 Rpl32 Rpl36 Rpl23a Rpl26 Rps7 Rpl35 Rpl31 Rpl24
Reactome	4.04E-09	7	16	40.22872	Metabolism of porphyrins	Hmox1 Cpox Fech Alas2 Urod Hmbs Ppox
Reactome	1.51E-08	6	11	50.15528	Heme biosynthesis	Cpox Fech Alas2 Urod Hmbs Ppox
Reactome	1.65E-07	23	539	4.154973	Metabolism of RNA	Hnrnpd Rps18 Rps15a Riok3 Rps14 Rps26 Nop56 Tut7 Rpl12 Rps21 Pcf11 Rpl18a Rpl29 Rps10 Rpl38 Rpl32 Rpl36 Rpl23a Rpl26 Rps7 Rpl35 Rpl31 Rpl24
Reactome	3.54E-06	8	60	12.90545	Ribosomal scanning and start codon recognition	Rps18 Rps15a Eif5 Rps14 Rps26 Rps21 Rps10 Rps7
Reactome	3.87E-06	8	61	12.68295	Activation of the mRNA upon binding of the cap-binding complex and eIFs and subs	Rps18 Rps15a Rps14 Rps26 Eif4ebp1 Rps21 Rps10 Rps7
Reactome	1.97E-05	7	53	12.87319	Formation of the ternary complex and subsequently the 43S complex	Rps18 Rps15a Rps14 Rps26 Rps21 Rps10 Rps7
Reactome	2.11E-05	3	3	91.95135	Erythrocytes take up oxygen and release carbon dioxide	Hbb-bs Hba-a1 Hbb-bt
Reactome	2.11E-05	18	512	3.813651	Cell Cycle Mitotic	Ube2c Ccne1 Cdc6 E2f2 Cdk1 Cdc25b Tpx2 E2f1 Smc2 Cenpa Ccnb2 Ncaph Wapl Ccnb1 Ncapg2 Psme3 H2ac23 H2ac24
Reactome	4.27E-05	7	60	11.29227	Translation initiation complex formation	Rps18 Rps15a Rps14 Rps26 Rps21 Rps10 Rps7
Reactome	7.67E-05	3	4	68.96351	G2/M DNA replication checkpoint	Cdk1 Ccnb2 Ccnb1
Reactome	0.000238	4	76	24.52036	Condensation of Prophase Chromosomes	Smc2 Ncapg2 H2ac23 H2ac24
Reactome	0.001026	18	632	2.819633	Transport of small molecules	Aqp1 Hmox1 Slc4a1 Slc3a2 Tfrc Bsg Rhag Stom Slc43a1 Car1 Car2 Add2 Slc38a5 Abcb10 Abcb4 Hbb-bs Hba-a1 Hbb-bt
Reactome	0.001599	4	24	15.32523	Cyclin A/B1/B2 associated events during G2/M transition	Cdk1 Cdc25b Ccnb2 Ccnb1
Reactome	0.002493	3	11	25.07764	CDC6 association with the ORC:origin complex	Cdc6 E2f2 E2f1
Reactome	0.002674	11	348	3.678054	M Phase	Ube2c Cdk1 Smc2 Cenpa Ccnb2 Ncaph Wapl Ccnb1 Ncapg2 H2ac23 H2ac24
Reactome	0.004581	4	93	11.49392	Binding and Uptake of Ligands by Scavenger Receptors	Cd36 Hbb-bs Hba-a1 Hbb-bt
Reactome	0.008903	3	17	16.22671	Chaperone Mediated Autophagy	Prkab1 Hbb-bs Hbb-bt
Reactome	0.010287	3	78	15.32523	Scavenging of heme from plasma	Hbb-bs Hba-a1 Hbb-bt
Reactome	0.011886	2	5	36.78054	Phosphorylation of Emi1	Cdk1 Ccnb1
Reactome	0.011886	2	5	36.78054	Condensation of Prometaphase Chromosomes	Smc2 Ncaph
Reactome	0.011886	2	66	36.78054	NoRC negatively regulates rRNA expression	H2ac23 H2ac24
Reactome	0.016789	11	429	2.87348	Cellular responses to external stimuli	Ube2c Ccne1 Prdx2 Dnajb1 Cat Ulk1 Prkab1 Gabarapl2 Gpx1 H2ac23 H2ac24
Reactome	0.016794	2	6	30.65045	Synthesis of 15-eicosatetraenoic acid derivatives	Gpx1 Gpx4
Reactome	0.018234	3	24	11.99365	APC/C:Cdc20 mediated degradation of Cyclin B	Ube2c Cdk1 Ccnb1
Reactome	0.019791	2	7	26.27181	TP53 Regulates Transcription of Genes Involved in G2 Cell Cycle Arrest	Cdk1 Ccnb1
Reactome	0.019791	2	68	26.27181	RNA Polymerase I Promoter Opening	H2ac23 H2ac24

Reactome	0.019791	2	60	26.27181	Packaging Of Telomere Ends	H2ac23 H2ac24
Reactome	0.019791	6	128	4.485432	DNA Replication	Ube2c Ccne1 Cdc6 E2f2 E2f1 Psme3
Reactome	0.02274	2	8	22.98784	Glutathione synthesis and recycling	Gclm Gclc
Reactome	0.02274	2	69	22.98784	Negative epigenetic reg. of rRNA expression	H2ac23 H2ac24
Reactome	0.02274	2	61	22.98784	Recognition and association of DNA glycosylase with site containing an affected	H2ac23 H2ac24
Reactome	0.02274	3	27	10.21682	Reg. of APC/C activators between G1/S and early anaphase	Ube2c Cdk1 Ccnb1
Reactome	0.02274	3	28	10.21682	CDK-mediated phosphorylation and removal of Cdc6	Ube2c Ccne1 Cdc6
Reactome	0.02274	3	28	10.21682	Glutathione conjugation	Mgst3 Gclm Gclc
Reactome	0.02274	29	1685	1.710448	Metabolism of proteins	Ube2c Rps18 Rps15a Cdk1 Eif5 Rps14 Rps26 Dcaf6 Blm Eif4ebp1 Ank1 Rpl12 Rps21 Rpl18a Rpl29 Rps10 St3gal5 Rpl38 Rpl32 Rpl36 Rpl23a Rpl26 Rps7 Rpl35 Rpl31 Psme3 H2ac23 H2ac24 Rpl24
Reactome	0.02337	5	95	4.997356	S Phase	Ube2c Ccne1 Cdc6 Cdc25b Wapl
Reactome	0.023573	3	28	9.851931	Microautophagy	Prkab1 Hbb-bs Hbb-bt
Reactome	0.024735	3	32	9.512209	Detoxification of Reactive Oxygen Species	Prdx2 Cat Gpx1
Reactome	0.024735	3	79	9.512209	Deposition of new CENPA-containing nucleosomes at the centromere	Cenpa H2ac23 H2ac24
Reactome	0.024735	3	79	9.512209	Nucleosome assembly	Cenpa H2ac23 H2ac24
Reactome	0.025195	2	70	20.43363	Activated PKN1 stimulates transcription of AR androgen receptor regulated genes	H2ac23 H2ac24
Reactome	0.025195	3	32	9.195135	APC/C:Cdc20 mediated degradation of mitotic proteins	Ube2c Cdk1 Ccnb1
Reactome	0.025195	3	31	9.195135	The role of GTSE1 in G2/M progression after G2 checkpoint	Cdk1 Ccnb2 Ccnb1
Reactome	0.025195	6	145	3.969123	Mitotic G2-G2/M phases	Cdk1 Cdc25b Tpx2 E2f1 Ccnb2 Ccnb1
Reactome	0.025925	3	94	8.898518	Positive epigenetic reg. of rRNA expression	Dek H2ac23 H2ac24
Reactome	0.025925	3	94	8.898518	B-WICH complex positively regulates rRNA expression	Dek H2ac23 H2ac24
Reactome	0.025925	3	89	8.898518	DNA Damage/Telomere Stress Induced Senescence	Ccne1 H2ac23 H2ac24
Reactome	0.025925	7	197	3.423721	Mitotic Prometaphase	Cdk1 Smc2 Cenpa Ccnb2 Ncaph Wapl Ccnb1
Reactome	0.02765	3	32	8.620439	Amino acid transport across the plasma membrane	Slc3a2 Slc43a1 Slc38a5
Reactome	0.02765	28	1696	1.666432	Metabolism	Cd36 Hmox1 Slc3a2 Pnpo Acsl1 Ndufb9 Cpox Bsg Fech Ndufb8 Alas2 Ptdss2 Acadl Mgst3 Car1 Car2 Gclm Urod Nt5c3 Stard10 Hmbs Gclc Isca1 Fabp4 Ppox Gpx1 Mocs1 Gpx4
Reactome	0.02937	7	236	3.317832	Cell Cycle Checkpoints	Ube2c Ccne1 Cdc6 Cdk1 Cenpa Ccnb2 Ccnb1
Reactome	0.029953	5	106	4.420738	Autophagy	Ulk1 Prkab1 Gabarapl2 Hbb-bs Hbb-bt
Reactome	0.030613	2	64	16.71843	Cleavage of the damaged purine	H2ac23 H2ac24
Reactome	0.030613	2	64	16.71843	Depurination	H2ac23 H2ac24
Reactome	0.030613	2	11	16.71843	Chk1/Chk2Cds1 mediated inactivation of Cyclin B:Cdk1 complex	Cdk1 Ccnb1
Reactome	0.030613	3	34	8.113355	TP53 Regulates Transcription of Cell Cycle Genes	Ccne1 Cdk1 Ccnb1
Reactome	0.030613	4	70	5.489633	Assembly of the pre-replicative complex	Cdc6 E2f2 E2f1 Psme3
Reactome	0.035853	2	12	15.32523	Reversible hydration of carbon dioxide	Car1 Car2
Reactome	0.036568	4	135	5.180358	Mitotic Prophase	Smc2 Ncapg2 H2ac23 H2ac24

Reactome	0.041956	3	101	7.073181	Senescence-Associated Secretory Phenotype SASP	Ube2c H2ac23 H2ac24
Reactome	0.043134	4	112	4.904072	G2/M Checkpoints	Cdc6 Cdk1 Ccnb2 Ccnb1
Reactome	0.045805	5	124	3.863502	Resolution of Sister Chromatid Cohesion	Cdk1 Cenpa Ccnb2 Wapl Ccnb1
Reactome	0.048166	4	81	4.715454	Mitotic G1-G1/S phases	Ccne1 Cdc6 E2f2 E2f1
Reactome	0.048538	4	83	4.655765	APC/C-mediated degradation of cell cycle proteins	Ube2c Cdk1 Ccnb1 Psme3
Reactome	0.048538	4	83	4.655765	Reg. of mitotic cell cycle	Ube2c Cdk1 Ccnb1 Psme3
Reactome	0.048538	8	351	2.674948	Cellular responses to stress	Ube2c Ccne1 Prdx2 Dnajb1 Cat Gpx1 H2ac23 H2ac24

SUPPLEMENTAL TABLE 6

Supplemental Table 6. RNAseq subanalysis. List of 'dispensible' genes for bone protection with >|0.35| Log_2fold change in both WT and Sarm1[KO] mice with T1D.

Gene ID	Gene Name	Description	Log2FC (WT T1D/WT Control)	Log2FC (KO T1D/KO Control)	pvalue diabetes
16612	*Klk1*	kallikrein 1	-1.92	-0.73	2.36E-06
108956	*Apol7c*	apolipoprotein L 7c	-1.64	-0.69	4.11E-03
326623	*Tnfsf15*	tumor necrosis factor (ligand) superfamily, member 15	-1.62	-1.51	1.97E-05
15945	*Cxcl10*	chemokine (C-X-C motif) ligand 10	-1.59	-0.59	1.30E-03
20296	*Ccl2*	chemokine (C-C motif) ligand 2	-1.56	-1.41	1.91E-05
246728	*Oas2*	2'-5' oligoadenylate synthetase 2	-1.32	-0.53	3.37E-05
23960	*Oas1g*	2'-5' oligoadenylate synthetase 1G	-1.24	-0.87	1.00E-03
214575	*Tdrd5*	tudor domain containing 5	-1.19	-0.84	4.05E-03
93873	*Pcdhb2*	protocadherin beta 2	-1.14	-1.13	5.89E-03
319998	*Tmem198*	transmembrane protein 198	-1.14	-0.47	1.74E-03
623121	*Ifi213*	interferon activated gene 213	-1.10	-0.55	8.19E-05
15959	*Ifit3*	interferon-induced protein with tetratricopeptide repeats 3	-1.09	-0.42	7.98E-04
245240	*9930111J21Rik2*	RIKEN cDNA 9930111J21 gene 2	-1.08	-0.51	3.21E-03
211612	*Ptchd1*	patched domain containing 1	-1.00	-0.66	7.58E-07
22174	*Tyro3*	TYRO3 protein tyrosine kinase 3	-1.00	-0.47	8.84E-04
11435	*Chrna1*	cholinergic receptor, nicotinic, alpha polypeptide 1 (muscle)	-0.96	-0.56	1.70E-03
246730	*Oas1a*	2'-5' oligoadenylate synthetase 1A	-0.92	-0.46	1.63E-04
11488	*Adam11*	a disintegrin and metallopeptidase domain 11	-0.92	-0.59	3.56E-04
22065	*Trpc3*	transient receptor potential cation channel, subfamily C, member 3	-0.91	-1.07	4.03E-03
574428	*Zmynd15*	zinc finger, MYND-type containing 15	-0.91	-0.48	4.86E-04
67564	*Tmem35a*	transmembrane protein 35A	-0.90	-0.54	1.46E-03
219132	*Phf11d*	PHD finger protein 11D	-0.89	-0.40	6.82E-03
637515	*Nlrp1b*	NLR family, pyrin domain containing 1B	-0.89	-0.54	3.15E-04
20682	*Sox9*	SRY (sex determining region Y)-box 9	-0.86	-0.73	8.97E-03
75973	*Ccdc162*	coiled-coil domain containing 162	-0.86	-0.40	2.91E-03
100702	*Gbp6*	guanylate binding protein 6	-0.84	-0.39	2.58E-03
66102	*Cxcl16*	chemokine (C-X-C motif) ligand 16	-0.83	-0.41	1.48E-03
271305	*Phf21b*	PHD finger protein 21B	-0.81	-0.63	5.54E-03
105246320	*Gm26637*	None	-0.81	-0.53	1.79E-04
17472	*Gbp4*	guanylate binding protein 4	-0.81	-0.43	1.50E-04
320844	*Amigo3*	adhesion molecule with Ig like domain 3	-0.80	-0.36	2.39E-03
231602	*P2rx2*	purinergic receptor P2X, ligand-gated ion channel, 2	-0.78	-0.79	1.17E-03
20250	*Scd2*	stearoyl-Coenzyme A desaturase 2	-0.78	-0.36	3.83E-04
102641031	*LOC102641031*	None	-0.76	-1.17	5.72E-03
12774	*Ccr5*	chemokine (C-C motif) receptor 5	-0.76	-0.42	2.22E-04
76933	*Ifi27l2a*	interferon, alpha-inducible protein 27 like 2A	-0.73	-0.46	4.68E-04
235472	*Prtg*	protogenin	-0.70	-0.57	8.17E-03
195434	*Utp14b*	UTP14B small subunit processome component	-0.68	-0.67	6.26E-04
327978	*Slfn5*	schlafen 5	-0.68	-0.43	6.98E-06
232035	*Ccser1*	coiled-coil serine rich 1	-0.68	-0.57	2.63E-03
211134	*Lzts1*	leucine zipper, putative tumor suppressor 1	-0.68	-0.42	3.20E-03
12504	*Cd4*	CD4 antigen	-0.67	-0.49	3.19E-05
432552	*Fam71b*	golgi associated RAB2 interactor 3	-0.64	-0.55	1.90E-04
18782	*Pla2g2d*	phospholipase A2, group IID	-0.64	-0.61	2.46E-03
14608	*Gpr83*	G protein-coupled receptor 83	-0.62	-0.59	9.13E-03

12212	*Chic1*	cysteine-rich hydrophobic domain 1	-0.62	-0.48	8.44E-03
240888	*Gpr161*	G protein-coupled receptor 161	-0.61	-0.58	7.56E-04
73707	*Gucy2g*	guanylate cyclase 2g	-0.61	-0.79	9.19E-03
12263	*C2*	complement component 2 (within H-2S)	-0.60	-0.37	1.66E-03
100039968	*Tmem35b*	transmembrane protein 35B	-0.59	-0.40	1.78E-04
105245342	*Gm40814*	None	-0.59	-0.50	8.36E-03
73340	*Nptxr*	neuronal pentraxin receptor	-0.58	-0.77	4.96E-03
449000	*Zfp960*	zinc finger protein 960	-0.57	-0.77	4.71E-03
240328	*F830016B08Rik*	RIKEN cDNA F830016B08 gene	-0.57	-0.41	9.71E-03
93887	*Pcdhb16*	protocadherin beta 16	-0.55	-0.47	2.76E-03
15957	*Ifit1*	interferon-induced protein with tetratricopeptide repeats 1	-0.55	-0.46	2.29E-03
215090	*Maneal*	mannosidase, endo-alpha-like	-0.54	-1.08	5.18E-03
214403	*Gm4788*	complement factor H-related 4	-0.54	-0.35	2.16E-03
93881	*Pcdhb10*	protocadherin beta 10	-0.52	-0.44	6.32E-03
208634	*Tspan10*	tetraspanin 10	-0.52	-1.01	2.27E-03
19225	*Ptgs2*	prostaglandin-endoperoxide synthase 2	-0.52	-0.82	6.92E-03
22612	*Yes1*	YES proto-oncogene 1, Src family tyrosine kinase	-0.51	-0.36	5.46E-04
218441	*Zfyve16*	zinc finger, FYVE domain containing 16	-0.50	-0.37	2.06E-04
234311	*Ddx60*	DExD/H box helicase 60	-0.48	-0.41	1.76E-03
93880	*Pcdhb9*	protocadherin beta 9	-0.46	-0.37	4.88E-03
105244832	*Gm40368*	None	-0.46	-0.39	1.22E-03
74443	*P4htm*	prolyl 4-hydroxylase, transmembrane (endoplasmic reticulum)	-0.46	-0.51	9.45E-03
69479	*1700029J07Rik*	RIKEN cDNA 1700029J07 gene	-0.45	-0.51	9.38E-03
80901	*Cxcr6*	chemokine (C-X-C motif) receptor 6	-0.45	-0.54	6.35E-03
18126	*Nos2*	nitric oxide synthase 2, inducible	-0.44	-0.50	3.72E-03
20474	*Six4*	sine oculis-related homeobox 4	-0.44	-0.58	9.14E-03
105245389	*Gm40853*	None	-0.44	-1.54	6.50E-03
21687	*Tek*	TEK receptor tyrosine kinase	-0.44	-0.47	9.22E-06
235386	*Hykk*	hydroxylysine kinase 1	-0.42	-0.83	3.29E-03
214547	*She*	src homology 2 domain-containing transforming protein E	-0.41	-0.36	4.83E-04
11480	*Acvr2a*	activin receptor IIA	-0.39	-0.51	1.08E-04
22715	*Zfp57*	zinc finger protein 57	-0.39	-0.75	7.56E-03
208748	*Prrg3*	proline rich Gla (G-carboxyglutamic acid) 3 (transmembrane)	-0.38	-0.75	5.77E-03
23792	*Adam23*	a disintegrin and metallopeptidase domain 23	-0.37	-0.59	8.50E-03
620078	*C130026I21Rik*	RIKEN cDNA C130026I21 gene	-0.36	-0.78	5.88E-03
194655	*Klf11*	Kruppel-like factor 11	0.59	0.38	2.55E-03
235631	*Prss50*	protease, serine 50	0.66	0.60	9.12E-03
21334	*Tac2*	tachykinin 2	0.73	0.57	4.90E-03
271047	*Serpina3b*	serine (or cysteine) peptidase inhibitor, clade A, member 3B	1.03	0.55	5.59E-03
11537	*Cfd*	complement factor D (adipsin)	1.22	1.04	1.38E-03
57875	*Angptl4*	angiopoietin-like 4	1.32	0.61	1.00E-04
278180	*Vsig4*	V-set and immunoglobulin domain containing 4	1.41	0.60	5.56E-03
71753	*Tmprss6*	transmembrane serine protease 6	1.60	1.47	2.90E-03
12346	*Car1*	carbonic anhydrase 1	1.79	0.46	9.20E-03
11517	*Adcyap1r1*	adenylate cyclase activating polypeptide 1 receptor 1	1.79	1.37	1.71E-04
72973	*Fbxo47*	F-box protein 47	2.28	0.45	8.59E-03
319189	*Hist2h2bb*	H2B clustered histone 18	2.42	0.57	3.62E-03

SUPPLEMENTAL TABLE 7

Supplemental Table 7. Pathway enrichment analysis results – based on genes downregulated in both WT and Sarm1-KO T1D as listed in Supplemental Table 6. Results from ShinyGO version 0.77, analyses including GO:Biological Process, GO:Molecular Function, KEGG, and Reactome. FDR=false discovery rate.

Analysis	Enrichment FDR	nGenes	Pathway Genes	Fold Enrichment	Pathway	Genes
GO:Biological Process	1.99E-06	24	1546	4.112945	Defense response	Yes1 Cxcl16 Nos2 Cd4 C2 Tyro3 Ptgs2 Oas2 Ifit1 Cxcl10 Ccl2 Ddx60 Pla2g2d Cxcr6 Oas1a Oas1g 9930111J21Rik2 Nlrp1b Ifi213 Ifit3 Ccr5 Gbp4 F830016B08Rik Gbp6
GO:Biological Process	7.67E-06	16	742	5.817995	Innate immune response	Yes1 Cxcl16 Nos2 C2 Tyro3 Oas2 Ifit1 Cxcl10 Ccl2 Oas1a Oas1g Nlrp1b Ifi213 Ifit3 Gbp4 Gbp6
GO:Biological Process	7.67E-06	18	1012	4.87806	Defense response to other organism	Yes1 Cxcl16 Nos2 Cd4 C2 Tyro3 Oas2 Ifit1 Cxcl10 Ccl2 Ddx60 Oas1a Oas1g Nlrp1b Ifi213 Ifit3 Gbp4 Gbp6
GO:Biological Process	7.67E-06	23	1619	3.748687	Immune response	Tek Yes1 Cxcl16 Nos2 Cd4 C2 Tyro3 Oas2 Ifit1 Cxcl10 Ccl2 Ddx60 Pla2g2d Cxcr6 Tnfsf15 Oas1a Oas1g Nlrp1b Ifi213 Ifit3 Ccr5 Gbp4 Gbp6
GO:Biological Process	9.09E-06	18	922	4.754706	Response to cytokine	Sox9 Cxcl16 Nos2 Cd4 Adam23 Ptgs2 Oas2 Ifit1 Cxcl10 Ccl2 Cxcr6 9930111J21Rik2 Ifi213 Ifit3 Ccr5 Gbp4 F830016B08Rik Gbp6
GO:Biological Process	1.67E-05	6	48	29.33755	Cellular response to interferon-beta	Ifit1 9930111J21Rik2 Ifi213 Ifit3 F830016B08Rik Gbp6
GO:Biological Process	2.67E-05	6	59	24.62259	Response to interferon-beta	Ifit1 9930111J21Rik2 Ifi213 Ifit3 F830016B08Rik Gbp6
GO:Biological Process	2.67E-05	16	811	4.800226	Cellular response to cytokine stimulus	Sox9 Nos2 Cd4 Adam23 Oas2 Ifit1 Cxcl10 Ccl2 Cxcr6 9930111J21Rik2 Ifi213 Ifit3 Ccr5 Gbp4 F830016B08Rik Gbp6
GO:Biological Process	2.67E-05	20	1400	3.807967	Response to other organism	Yes1 Cxcl16 Nos2 Cd4 C2 Tyro3 Ptgs2 Oas2 Ifit1 Cxcl10 Ccl2 Ddx60 Oas1a Oas1g Nlrp1b Ifi213 Ifit3 Ifi27l2a Gbp4 Gbp6
GO:Biological Process	2.67E-05	20	1403	3.798526	Response to external biotic stimulus	Yes1 Cxcl16 Nos2 Cd4 C2 Tyro3 Ptgs2 Oas2 Ifit1 Cxcl10 Ccl2 Ddx60 Oas1a Oas1g Nlrp1b Ifi213 Ifit3 Ifi27l2a Gbp4 Gbp6
GO:Biological Process	2.67E-05	21	1524	3.647791	Biological proc. involved in interspecies interaction between organisms	Yes1 Cxcl16 Nos2 Cd4 C2 Tyro3 Ptgs2 Oas2 Ifit1 Cxcl10 Ccl2 Ddx60 Oas1a Oas1g Nlrp1b Ifi213 Ifit3 Ifi27l2a Ccr5 Gbp4 Gbp6
GO:Biological Process	8.32E-05	9	254	9.111442	Defense response to symbiont	Oas2 Ifit1 Cxcl10 Ddx60 Oas1a Oas1g Nlrp1b Ifit3 Gbp4
GO:Biological Process	9.19E-05	10	330	7.609629	Response to virus	Oas2 Ifit1 Cxcl10 Ddx60 Oas1a Oas1g Nlrp1b Ifit3 Ifi27l2a Gbp4
GO:Biological Process	0.002131696	4	36	25.53453	Response to ATP	Trpc3 P2rx2 Ptgs2 Ccl2
GO:Biological Process	0.002860985	13	821	3.87489	Cell-cell adhesion	Sox9 Tek Cd4 Tyro3 Amigo3 Ccl2 Prtg Pla2g2d Pcdhb10 Pcdhb16 Pcdhb9 Pcdhb2 Ccr5
GO:Biological Process	0.003010249	3	15	49.24517	Reg. of ribonuclease activity	Oas2 Oas1a Oas1g
GO:Biological Process	0.006450352	11	745	4.137347	Response to bacterium	Cxcl16 Nos2 Cd4 Ptgs2 Oas2 Ifit1 Cxcl10 Ccl2 Ifit3 Gbp4 Gbp6
GO:Biological Process	0.009276117	4	64	16.71351	Killing of cells of other organism	Nos2 Cxcl10 Ccl2 Ccr5
GO:Biological Process	0.010646401	3	50	31.33784	Sex determination	Sox9 Six4 Zfp57
GO:Biological Process	0.016199768	4	69	14.1422	Response to fatty acid	Sox9 Scd2 Ptgs2 Ccl2
GO:Biological Process	0.022487246	2	6	76.6036	Pos. reg. of apoptotic cell clearance	C2 Ccl2
GO:Biological Process	0.023469679	5	142	8.511512	Response to interferon-gamma	Cxcl16 Nos2 Ccl2 Gbp4 Gbp6
GO:Biological Process	0.023469679	5	142	8.448927	Response to organophosphorus	Tek Trpc3 P2rx2 Ptgs2 Ccl2
GO:Biological Process	0.023469679	5	148	8.387256	Reg. of viral life cycle	Cd4 Tyro3 Oas2 Oas1a Oas1g

GO:Biological Process	0.023944362	4	81	11.93822	Chemokine-mediated signaling pathway	Cxcl10 Ccl2 Cxcr6 Ccr5
GO:Biological Process	0.023944362	12	967	3.162534	Response to lipid	Sox9 Tek Yes1 Cxcl16 Nos2 Scd2 Gpr83 Ptgs2 Cxcl10 Ccl2 Ccr5 Gbp6
GO:Biological Process	0.024229608	15	1374	2.674292	Biological adhesion	Sox9 Tek Cd4 Adam23 Tyro3 Amigo3 Ccl2 Prtg Pla2g2d Pcdhb10 Pcdhb16 Pcdhb9 Pcdhb2 Ccr5 Gbp6
GO:Biological Process	0.03035627	2	8	57.4527	Pos. reg. of fever generation	Ptgs2 Ccr5
GO:Biological Process	0.03035627	5	168	7.609629	Homophilic cell adhesion via plasma membrane adhesion molecules	Prtg Pcdhb10 Pcdhb16 Pcdhb9 Pcdhb2
GO:Biological Process	0.030921748	5	165	7.510157	Reg. of viral proc.	Cd4 Tyro3 Oas2 Oas1a Oas1g
GO:Biological Process	0.031622045	4	92	10.44595	Response to chemokine	Cxcl10 Ccl2 Cxcr6 Ccr5
GO:Biological Process	0.031622045	4	92	10.44595	Cellular response to chemokine	Cxcl10 Ccl2 Cxcr6 Ccr5
GO:Biological Process	0.033899178	2	9	51.06907	Modulation by virus of host cellular proc.	Cd4 Ccr5
GO:Biological Process	0.033899178	3	40	17.23581	Neuron cellular homeostasis	Chrna1 Tyro3 P2rx2
GO:Biological Process	0.033899178	5	167	7.136982	Response to purine-containing compound	Tek Trpc3 P2rx2 Ptgs2 Ccl2
GO:Biological Process	0.033899178	6	274	5.62802	Cell-cell adhesion via plasma-membrane adhesion molecules	Amigo3 Prtg Pcdhb10 Pcdhb16 Pcdhb9 Pcdhb2
GO:Biological Process	0.03811686	2	11	45.96216	Antiviral innate immune response	Cxcl10 Nlrp1b
GO:Biological Process	0.03811686	2	10	45.96216	Reg. of fever generation	Ptgs2 Ccr5
GO:Biological Process	0.044038239	3	48	15.32072	Reg. of acute inflammatory response	Ptgs2 Pla2g2d Ccr5
GO:Biological Process	0.044038239	4	108	9.012189	Response to retinoic acid	Sox9 Tek Yes1 Ccl2
GO:Biological Process	0.046201218	14	1362	2.517489	Cell adhesion	Sox9 Tek Cd4 Adam23 Tyro3 Amigo3 Ccl2 Prtg Pla2g2d Pcdhb10 Pcdhb16 Pcdhb9 Pcdhb2 Ccr5
GO:Biological Process	0.047648899	2	13	38.3018	Pos. reg. of killing of cells of other organism	Nos2 Ccr5
GO:Biological Process	0.047648899	3	48	14.36318	Apoptotic cell clearance	C2 Tyro3 Ccl2
GO:Biological Process	0.047648899	3	53	14.36318	Neg. reg. of viral genome replication	Oas2 Oas1a Oas1g
GO:Molecular Function	0.002349242	3	11	68.94324	2 -5 -oligoadenylate synthetase activity	Oas2 Oas1a Oas1g
GO:Molecular Function	0.018962591	3	25	28.72635	Coreceptor activity	Cd4 Cxcr6 Acvr2a
GO:Molecular Function	0.027169617	3	36	20.27742	Adenylyltransferase activity	Oas2 Oas1a Oas1g
GO:Molecular Function	0.027169617	4	80	11.78517	Double-stranded RNA binding	Oas2 Ddx60 Oas1a Oas1g
GO:Molecular Function	0.037884111	3	45	16.81543	Chemokine activity	Cxcl16 Cxcl10 Ccl2
KEGG	0.010482752	5	159	8.448927	Hepatitis C	Oas2 Ifit1 Cxcl10 Oas1a Oas1g
KEGG	0.010794039	5	169	7.816694	Influenza A	Oas2 Cxcl10 Ccl2 Oas1a Oas1g
KEGG	0.010794039	5	202	7.510157	NOD-like receptor signaling pathway	Oas2 Ccl2 Oas1a Oas1g Nlrp1b
KEGG	0.001236357	8	289	7.38348	Cytokine-cytokine receptor interaction	Cxcl16 Cd4 Cxcl10 Ccl2 Cxcr6 Tnfsf15 Acvr2a Ccr5
KEGG	0.010482752	6	231	6.629158	Coronavirus disease	C2 Oas2 Cxcl10 Ccl2 Oas1a Oas1g
KEGG	0.019643227	5	189	6.313484	Chemokine signaling pathway	Cxcl16 Cxcl10 Ccl2 Cxcr6 Ccr5
Reactome	0.004598409	4	55	20.89189	Chemokine receptors bind chemokines	Cxcl16 Cxcl10 Cxcr6 Ccr5

SUPPLEMENTAL TABLE 8

Supplemental Table 8. Studies associating gene variants near Sarm1 with bone mineral density. Results from the Open Targets database version 22.10.

Study ID	Reported Trait	PMID	Author	Date	Study N Initial	Index Variant ID	Index Variant RSID	P-Value
GCST90025982	Heel bone mineral density T score	PMID:34226706	Barton AR	7/5/2021	445855 – UK Biobank	17_28367840_G_A	rs704	8.80E-21
GCST007066	Heel bone mineral density	PMID:30595370	Kichaev G	12/27/2018	446000 – UK Biobank	17_28367840_G_A	rs704	2.00E-20
GCST006979	Heel bone mineral density	PMID:30598549	Morris JA	12/31/2018	426824 – UK Biobank	17_28367840_G_A	rs704	7.10E-14
GCST006433	Heel bone mineral density	PMID:30048462	Kim SK	7/26/2018	394929 – UK Biobank	17_28367840_G_A	rs704	4.00E-16
GCST008603	Total body bone mineral density (MTAG)	PMID:30690781	Pei YF	2/28/2019	66628 - Genetic Factors for Osteoporosis (GEFOS) Consortium	17_28349109_C_T	rs13469	1.00E-08
